# Supplementary material for: An apical membrane complex for triggering rhoptry exocytosis and invasion in Toxoplasma
Source: EMBO J. 2022 Oct 17;41(22):e111158. doi: 10.15252/embj.2022111158 (PMC9670195; doi:10.15252/embj.2022111158)
Supplement: Supplementary file 1 — Expanded View Figures PDF [file EMBJ-41-e111158-s011.pdf]

Expanded View Figures

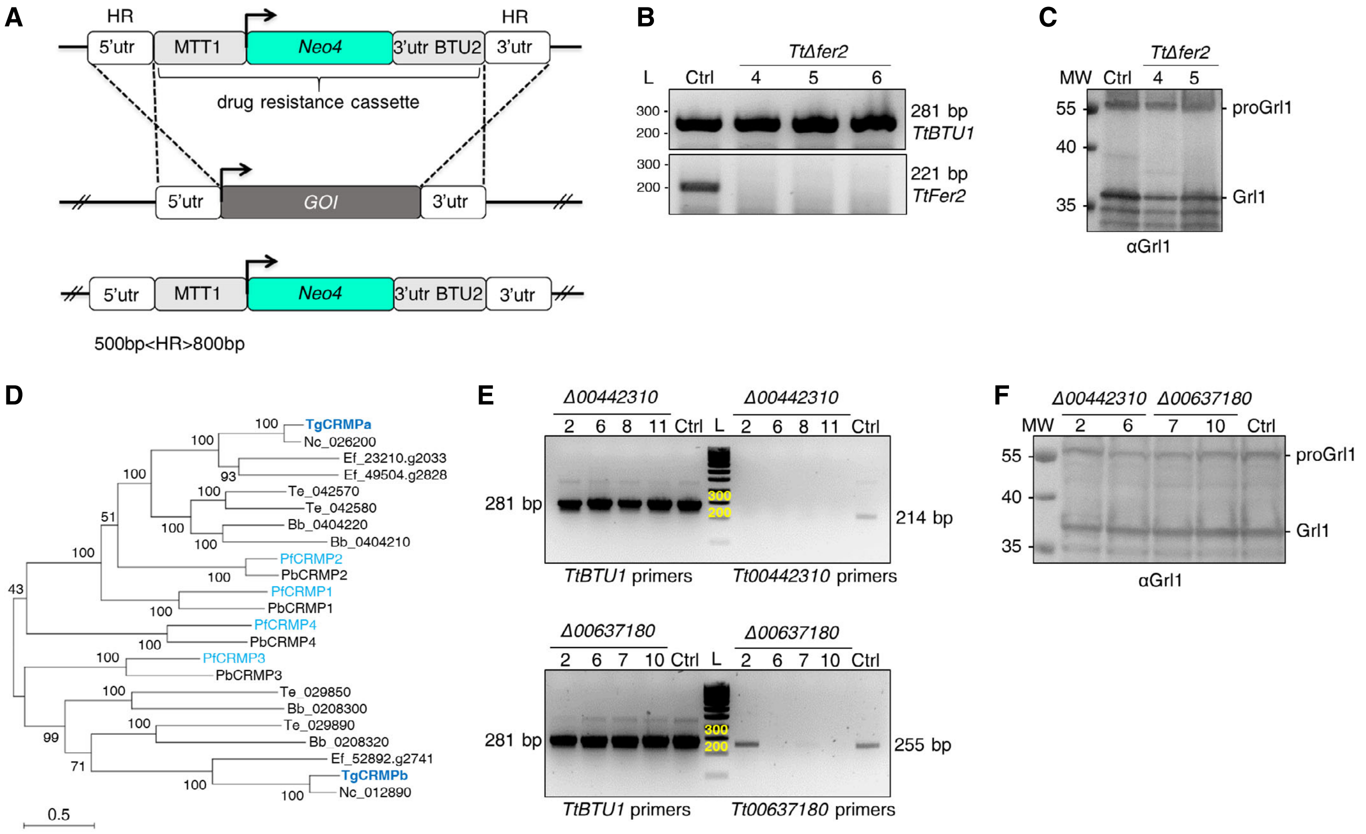

Figure EV1. *Tetrahymena* TtFer2, Tt00442310, and Tt00637180 are essential for mucocyst secretion (related to Fig 1).

A Strategy for the macronuclear knockout of *Tetrahymena thermophila* genes of interest (GOI). A linearized construct carrying fragments (HR) homologous to the 5′ and 3′-untranslated regions (UTR) of the GOI and flanking the drug resistance cassette were used to replace the GOI at the endogenous locus. The $CdCl_2$-inducible MTT1 promoter drives the expression of a paromomycin resistance gene (Neo4) used for selecting positive transformants.

B Disruption of the macronuclear copies of *TtFer2* (ferlin 2; TTHERM_00886960) was assessed by RT–PCR. cDNA from wild-type (Ctrl) and three clones of putative knockout cells (*Δfer2)* were PCR amplified with primers specific for *TtBTU1* (β-tubulin 1; upper panel) and *TtFer2* (lower panel). The 221 bp products corresponding to transcripts from *Fer2* are absent in the *Δfer2* clones, indicating that all the wild-type copies of *TtFer2* were efficiently replaced with the Neo4 cassette. All samples showed wild-type levels of *BTU1* transcripts. L: DNA ladder (bp). Primers are listed in Table EV1.

C Western blot of whole-cell lysates from wild-type (Ctrl) and *Δfer2* cells. In both, wild-type and mutant extracts, anti-Grl1 antibodies recognized the ~ 60 kDa precursor of the granule protein 1, proGrl1, and the processed form of Grl1, between 35 and 40 kDa, indicating non-significant defects in proteolytic maturation. MW: molecular weight standards.

D Phylogeny depicting the relationships between Apicomplexa CRMPs. The maximum-likelihood phylogenetic tree was obtained with the protein sequences of *CRMP* genes retrieved for the apicomplexans *Toxoplasma gondii* (TgCRMP), *Plasmodium falciparum* (PfCRMP), *Plasmodium berghei* (PbCRMP), *Neospora caninum* (Nc), *Eimeria falciformis* (Ef), *Theileria equi* (Te), and *Babesia bigemina* (Bb). *Toxoplasma* and *P. falciparum* CRMPs are highlighted in bold blue and light blue, respectively. Numbers at each node correspond to the bootstrap values. The scale bar represents the branch length.

E Disruption of the macronuclear copies of TTHERM_00442310 and TTHERM_00637180 was assessed by RT–PCR as in (B). Four clones for each putative knockout cell were tested. The 214 and 255 bp fragments corresponding to transcripts for TTHERM_00442310 and TTHERM_00637180, respectively, are absent in all *Δ00442310* clones, and nearly undetectable in clones 6, 7, and 10 for *Δ00637180*, indicating the achievement of full knockout. Clones 2 and 6 for *Δ00442310* and clones 7 and 10 for *Δ00637180* were selected for further analysis. All samples show wild-type levels of *BTU1* transcripts. L: DNA ladder (bp). Primers are listed in Table EV1.

F Western blot of whole-cell lysates from wild-type (Ctrl), *Δ00442310*, and *Δ00637180* cells. In both wild-type and mutant extracts, anti-Grl1 antibodies recognized processed Grl1 between 35 and 40 kDa and the precursor proGrl1 at ~ 60 kDa, indicating non-significant defects in proteolytic maturation. MW: molecular weight standards.

Source data are available online for this figure.

© 2022 The Authors

Figure EV2. TgCRMPa- and TgCRMPb-depleted tachyzoites have normal rhoptries, and show no defects in replication, stimulated egress, and attachment (related to Fig 2).

A Strategy for tagging genes of interest (GOI) in *Toxoplasma*. To generate C-terminal HA_3-fusions of TgCRMPa and TgCRMPb, a DNA fragment was amplified from a donor vector containing the HA_3 tag and the drug resistance cassette (CAT). Primers to amplify the DNA fragment were designed to contain ~ 30-bp-long stretches (HR) homologous to the GOI regions flanking the insertion site for the epitope tag. Upon CRISPR-cas9 cut (scissors), the PCR-amplified DNA fragment efficiently recombines into the targeted endogenous locus. The arrows indicate the binding sites of the primers used in (B).

B Integration of the HA_3 tag and CAT cassette at the C-terminus of TgCRMPa (upper panel) and TgCRMPb (lower panel) was tested by PCR. Genomic DNA from the untagged line and a clonal population for each of the putative HA_3-tagged lines was amplified with primers binding to the 3′ C-terminus and 3′UTR of each TgCRMP gene, in pairwise combination with primers binding the HA_3 and CAT sequences, respectively. The fragments corresponding to the HA_3 tag (5′) and the resistance cassette (3′) were correctly amplified in the putative tagged lines, indicating that they were efficiently integrated at the TgCRMPs loci. As expected, the wild-type fragment of each gene (wt) was detected only in the untagged line. L: DNA ladder (bp). Primers are listed in Table EV1.

C Strategies for the inducible depletion (iKD) of genes of interest (GOI) in *Toxoplasma*. The iKD lines for TgCRMPs were generated starting from the HA_3-tagged lines previously produced. In order to conditionally deplete the proteins, the endogenous promoter of each gene was replaced with an ATc-regulatable TetOSag4 promoter, preceded by the DHFR resistance cassette. The DNA fragment containing the cassette and the promoter was PCR amplified from a donor vector with primers containing ~ 30-bp-long homology regions (HR) specific for each gene and introduced upstream the starting codon via CRISPR-cas9 technology (scissors) and homologous recombination. The arrows indicate the binding sites of the primers used in (D) and Fig EV3G.

D Integration of the TetOSag4 promoter in TgCRMPa-HA_3 (upper panel) and TgCRMPb-HA_3 (lower panel) lines were tested by PCR. Integration of the DHFR resistance cassette was successfully PCR-amplified only for TgCRMPb-HA_3 (lower panel) line. Genomic DNA from untagged parasites and putative TgCRMPa_iKD and TgCRMPb_iKD clonal populations was amplified with primers binding to the 5′UTR and 5′ N-terminus of the GOI, flanking the DHFR-TetOSag4 insert, and used also in pairwise combination with primers binding the DHFR cassette and the TetOSag4 promoter, respectively. The fragments corresponding to the DHFR integration (5′) and TetOSag4 integration (3′) were detected exclusively in the putative iKD lines, while the wild-type fragment (wt) was amplified only in the untagged line. L: DNA ladder (bp). Primers are listed in Table EV1.

E Immunofluorescence images of untagged, TgCRMPa-HA_3, and TgCRMPb-HA_3 lines and TgCRMPs-depleted (iKD) intracellular tachyzoites. Parasites were stained with anti-HA and anti-ARM (ARO) Abs to visualize TgCRMPs and rhoptries, respectively. The nuclei (DNA) are stained with Hoechst. TgCRMPs pattern mirrors that of Fig 2B. Rhoptries show a wild-type appearance in the TgCRMPs-depleted parasites. Shown are single focal planes.

F Confocal immunofluorescence images of TgCRMPb-depleted (iKD) intracellular tachyzoites. Parasites were stained with anti-HA and with anti-GAMA and anti-PLP1 Abs to visualize TgCRMPb and micronemes, respectively. The nuclei (DNA) are stained with Hoechst. Shown are single focal planes.

G Extent of co-localization between TgCRMPb-HA_3 (light red) and microneme proteins AMA1, MIC2, GAMA, and PLP1 shown in (F) and Fig 2D. Untagged parasites were analyzed in parallel to estimate the background noise (light gray), and the extent of overlap between the microneme proteins AMA1 and MIC2 (dark gray). Pearson's correlation coefficient was measured using the Fiji-JACoP plugin. Values are expressed as mean ± SD; *n*, number of parasites analyzed.

H Replication measured for TgCRMPa- and TgCRMPb-depleted parasites. The percentage of vacuoles with 2, 4, 8, 16, and 32 parasites was calculated for control (Ctrl), and TgCRMPa_iKD and TgCRMPb_iKD lines, in the absence of ATc and upon 48 and 24 h ATc treatment, respectively. Both iKD mutants (+ATc) are capable of efficient replication. Data are reported as mean ± SD ($n = 2$ biological replicates, each with three technical replicates).

I Stimulated egress was quantified for TgCRMPa- and TgCRMPb-depleted parasites. Infected cells with intact vacuoles were treated with A23187 to induce parasite egress, measured as a number of burst vacuoles over the total number of vacuoles. Egress was tested for control (Ctrl), TgCRMPa_iKD and TgCRMPb_iKD lines, in the absence of ATc and upon 48 and 24 h ATc treatment, respectively. Values are reported as mean ± SD ($n = 3$ biological replicates, each with three technical replicates). The biological replicates are represented by different symbols. *P*-values are non-significant for all datasets (two-tailed *t*-test).

J Attachment measured for TgCRMPa- and TgCRMPb-depleted parasites. The number of parasites attached to the host cell was counted for control (Ctrl), TgCRMPa_iKD and TgCRMPb_iKD lines, in the absence of ATc and upon 48 and 24 h ATc treatment, respectively. BAPTA treatment was used as a control since it prevents attachment. TgCRMPa- and TgCRMPb-depleted parasites were able to attach to host cells. Values are reported as in (I; $n = 3$ biological replicates, each with three technical replicates). The biological replicates are represented by different symbols. *P*-values were measured by a two-tailed *t*-test.

Source data are available online for this figure.

 © 2022 The Authors

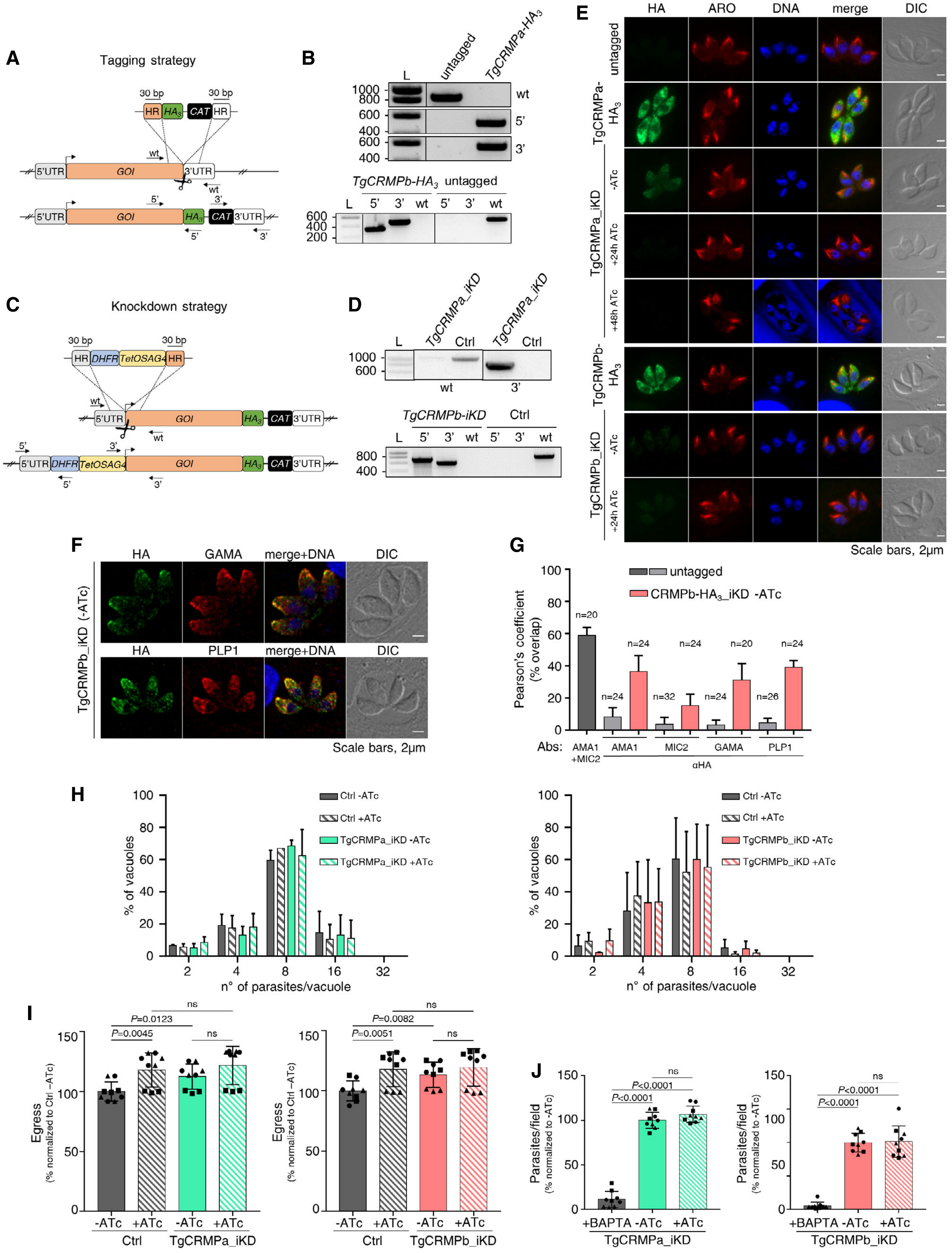
A
Tagging strategy
B
untagged
TgCRMPa-HA3
TgCRMPb-HA3 untagged
C
Knockdown strategy
D
TgCRMPa_iKD
Ctrl
TgCRMPb-iKD
E
HA
ARO
DNA
merge
DIC
untagged
TgCRMPa-HA3
TgCRMPa_iKD
TgCRMPb-HA3
TgCRMPb_iKD
Scale bars, 2μm
F
GAMA
PLP1
merge+DNA
TgCRMPb_iKD (-ATc)
G
untagged
CRMPb-HA3_iKD -ATc
Pearson's coefficient (% overlap)
H
% of vacuoles
n° of parasites/vacuole
Ctrl -ATc
Ctrl +ATc
TgCRMPa_iKD -ATc
TgCRMPa_iKD +ATc
TgCRMPb_iKD -ATc
TgCRMPb_iKD +ATc
I
Egress (% normalized to Ctrl -ATc)
J
Parasites/field (% normalized to -ATc)

Figure EV2.

Figure EV3. Tg277910-depleted tachyzoites with a disrupted lytic cycle show no defects in microneme staining, replication, stimulated egress, and attachment (related to Fig 3).

A Coomassie Blue staining of eluted proteins (1, 3, 5) immunoprecipitated (IP) with anti-HA beads, and protein fractions of the corresponding clear lysates (CL; 2, 4, 6) prior to beads incubation, from TgCRMPa-HA_3, TgCRMPb-HA_3, and untagged lines. The TgCRMP protein used as bait in each IP lane is indicated by the asterisk. Samples in lanes 1, 3, and 5 were analyzed by mass spectrometry. MW: molecular weight standards.

B Marker-free strategy for $FLAG_3$ tagging of TgCRMPa. To generate a C-terminal $FLAG_3$ fusion of TgCRMPa, a gBlock containing the $FLAG_3$ tag flanked by ~ 30-bp-long TgCRMPa homology regions (HR) was amplified and integrated into the TgCRMPa endogenous locus via CRISPR-cas9 technology (scissors). The $FLAG_3$-tagged TgCRMPa was generated also in the TgCRMPb-HA_3 line. The arrows indicate the binding sites of the primers used in (C).

C Integration of the $FLAG_3$ tag was tested by PCR in putative TgCRMPa-$FLAG_3$ and TgCRMPa-$FLAG_3$ + TgCRMPb-HA_3 lines. The addition of the tag at the C-terminus of the TgCRMPa gene corresponds to the insertion of an additional 74 bp to the wild-type sequence. A higher band was observed in the putative tagged lines compared to the untagged ones. DNA ladder (L) is shown on the left of each panel. Primers are listed in Table EV1.

D Eluates from Fig 3B and 1/20 of the clear lysates (before beads incubation) were also immunoblotted with anti-ROP5 antibodies to confirm the specificity of the immunoprecipitation experiments. The red arrowhead indicates TgROP5 protein, and the asterisk indicates unspecific bands detected in the eluates, likely corresponding to the light chain of the beads-conjugated antibody. MW: molecular weight standards.

E Strategy based on the pLIC system (Huynh & Carruthers, 2009) for tagging Tg277910 with triple HA. The arrows indicate the binding sites of the primers used in (F).

F Integration of the HA_3 tag and CAT cassette at the C-terminus of TGGT1_277910 was tested by PCR. Genomic DNA from an untagged line and a clonal population for the putative HA_3-tagged line were amplified with primers binding to the 3′ C-terminus of TGGT1_277910 and HA_3 sequence. The HA_3 tag (5′) was correctly amplified indicating that it was efficiently integrated at the TGGT1_277910 locus. L: DNA ladder (bp). Primers are listed in Table EV1.

G Integration of the DHFR cassette followed by the TetOSag4 promoter in TGGT1_277910 line was tested by PCR as in Fig EV2C and D. Genomic DNA from untagged parasites and putative Tg277910_iKD clonal population was amplified with primers binding the gene's 5′UTR and 5′ N-terminus, flanking the DHFR-TetOSag4 insert, and used also in pairwise combination with primers binding the DHFR cassette and the Sag4 promoter, respectively. The wild-type fragment (wt) was amplified only in the control line (Ctrl), while the fragments corresponding to the DHFR integration (5′) and TetOSag4 integration (3′) were detected exclusively in the putative iKD line. A low-abundant unspecific band of similar size to the 3′ fragments was observed in the untagged line. L: DNA ladder (bp). Primers are listed in Table EV1.

H Whole-cell lysates were collected from Tg277910-HA_3 parasites (HA_3) and from the line generated for the inducible knockdown (iKD) treated with ATc for 24, 48, and 72 h and untreated. The samples were immunoblotted with anti-HA Abs (upper panel) to visualize Tg277910 protein under all mentioned conditions. TgROP5 was used as a loading control (lower panel). A band corresponding to the predicted size for Tg277910 (~ 138 kDa) was detected in the untreated samples (−) and decreased overtime in the ATc-treated ones (+) to completely disappear upon 72 h of ATc treatment. Protein molecular weight standards (MW) are shown on the left of each panel.

I Immunofluorescence images of untagged and Tg277910-HA_3- and Tg277910-depleted (iKD) intracellular tachyzoites. Parasites were stained with anti-HA and anti-AMA1 Abs to label Tg277910 and micronemes, respectively. The nuclei (DNA) are stained with Hoechst. Tg277910-HA_3 pattern mirrors that of Fig 3D. Micronemes show a wild-type appearance in the Tg277910-depleted parasites. Shown are single focal planes.

J Quantification of plaques for Tg277910-depleted parasites. Lysis plaque areas were measured for untreated and 72 h ATc-treated control and iKD lines. Values are reported as mean ± SD (n = 3 biological replicates, each with three technical replicates). The biological replicates are represented by different symbols.

K–M Quantification of replication (K), stimulated egress (L), and attachment (M) for control (Ctrl) and Tg277910-depleted (iKD) lines were performed as in Fig EV2H–J, respectively, with 72 h ATc-treated and untreated parasites. Tg277910-depleted parasites replicate, egress, and attach normally. Values are reported as in (J; replication and egress: n = 2 biological replicates, each with three technical replicates; attachment: n = 3 biological replicates, each with three technical replicates). The biological replicates are represented by different symbols.

Data information: *P*-values in (J and M) were measured by a two-tailed *t*-test.
Source data are available online for this figure.

 © 2022 The Authors

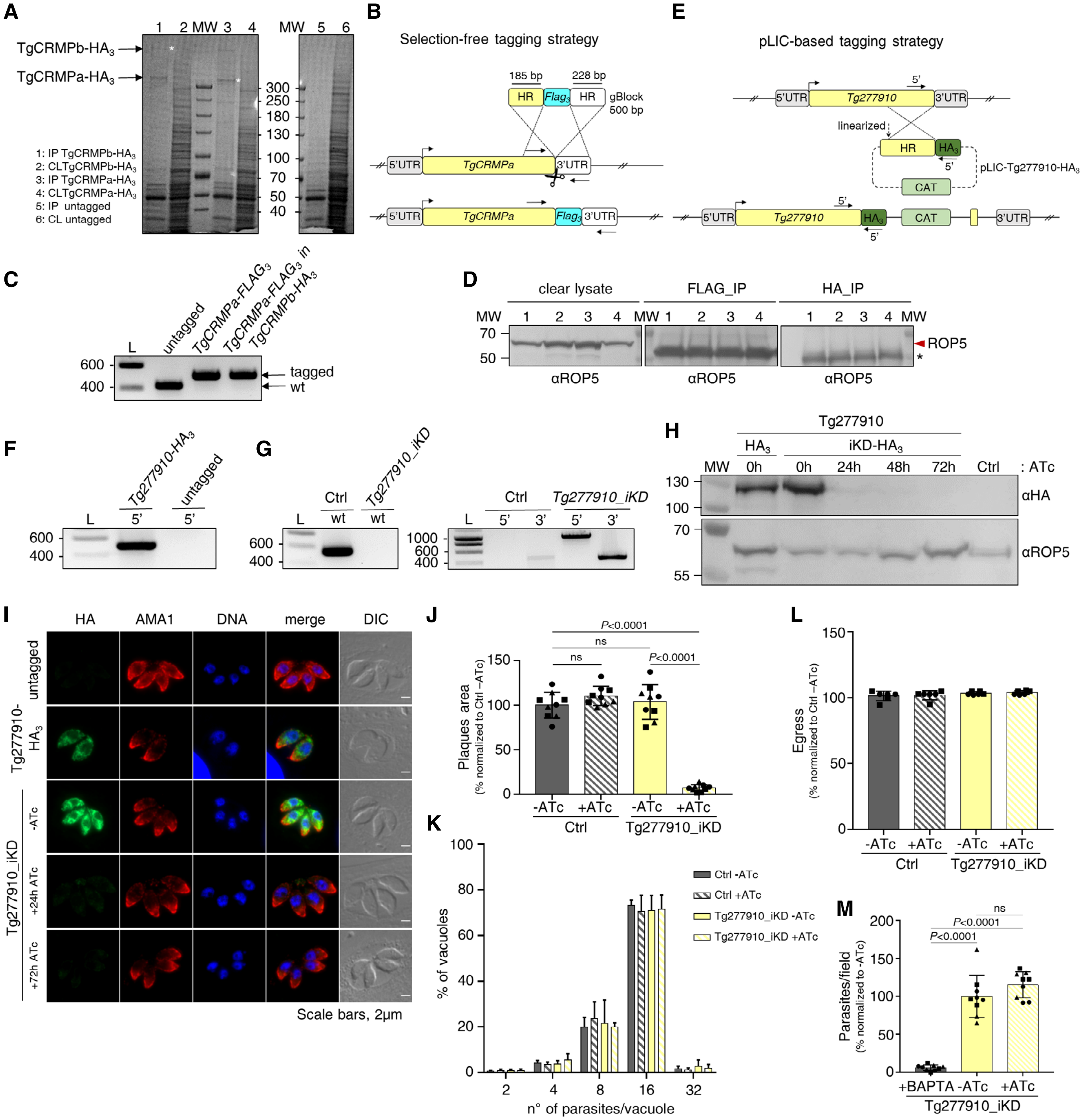
A
TgCRMPb-HA3
TgCRMPa-HA3
1: IP TgCRMPb-HA3
2: CLTgCRMPb-HA3
3: IP TgCRMPa-HA3
4: CLTgCRMPa-HA3
5: IP untagged
6: CL untagged
B
Selection-free tagging strategy
gBlock 500 bp
E
pLIC-based tagging strategy
pLIC-Tg277910-HA3
C
untagged
TgCRMPa-FLAG3
TgCRMPa-FLAG3 in TgCRMPb-HA3
tagged
wt
D
clear lysate
FLAG_IP
HA_IP
αROP5
ROP5
F
Tg277910-HA3
untagged
G
Tg277910_iKD
H
Tg277910
iKD-HA3
αHA
αROP5
I
HA AMA1 DNA merge DIC
untagged
Tg277910-HA3
Tg277910_iKD
Scale bars, 2μm
J
Plaques area (% normalized to Ctrl −ATc)
K
% of vacuoles
n° of parasites/vacuole
Ctrl -ATc
Ctrl +ATc
Tg277910_iKD -ATc
Tg277910_iKD +ATc
L
Egress (% normalized to Ctrl −ATc)
M
Parasites/field (% normalized to -ATc)
+BAPTA -ATc +ATc
Tg277910_iKD

Figure EV3.

Figure EV4. TgCRMPa and TgCRMPb accumulate at the tip of the extruded conoid (related to Fig 5).

A Quantification of the dot pattern for TgCRMPa-HA_3 in TgCRMPa-depleted (iKD) tachyzoites. TgCRMPa accumulation at the apical tip of extracellular parasites was measured upon incubation with host cell monolayers for 2 min to stimulate natural conoid extrusion. CRMPa signal at the apical dot disappeared after 48 h ATc treatment, indicating that the association with the tip of the extruded conoid was specific. No significant apical signal was detected for the control line (Ctrl), as in Fig 5B. Numbers are expressed as a percentage of parasites showing (dot) or lacking (no dot) the tip accumulation of TgCRMPa. The number of parasites (*n*) analyzed for each line is reported on the column tops.

B Strategy for the inducible depletion (iKD) of TgCRMPa-$FLAG_3$. The iKD lines were generated starting from the $FLAG_3$-tagged lines previously produced. In order to conditionally deplete the protein, the endogenous promoter of the TgCRMPa-$FLAG_3$ gene was replaced with an ATc-regulableTetOSag4 promoter, preceded by the DHFR resistance cassette. The DNA fragment containing the cassette and promoter was PCR-amplified from a donor vector with primers containing ~ 30-bp-long homology regions (HR) specific for *TgCRMPa* gene, and introduced upstream to the starting codon via CRISPR-cas9 technology (scissors) and homologous recombination. The arrows indicate the binding sites of the primers used in (C).

C Integration of the DHFR cassette followed by the TetOSag4 promoter in the putative TgCRMPa-$FLAG_3$ and TgCRMPa-$FLAG_3$ + TgCRMPb-$FLAG_3$ iKD lines was tested by PCR as in Fig EV2C and D (upper panel). The fragments corresponding to the DHFR integration (5′) and TetOSag4 integration (3′) were detected exclusively in the putative iKD lines, while the wild-type fragment (wt) was amplified only in the control line (Ctrl). L: DNA ladder (bp). Primers are listed in Table EV1.

D Whole-cell lysates from untagged and TgCRMPa-$FLAG_3$_iKD and TgCRMPa-$FLAG_3$_iKD + TgCRMPb-HA_3 lines were immunoblotted with anti-FLAG Abs to visualize tagged CRMPa in ATc-treated and untreated samples. CRMPa disappeared upon 48 h ATc incubation in both lines. A ~ 300 kDa unspecific cross-reactive band was observed in all samples. MW: molecular weight standards.

E Auxin-degron strategy used for generating TgCRMPa-miniAID-HA_3 strain. The integration of the tag and drug resistance cassette into the *TgCRMPa* locus is ensured by ~ 30-bp-long homology regions (HR) upon CRISPR-Cas9 activity (scissors). The arrows indicate the binding sites of the primers used in (F).

F Integration of the miniAID-HA_3 and HXGPRT cassette at the *TgCRMPa* locus in the Tir-1 line was tested by PCR as in Fig EV2A and B (upper panel). The fragments corresponding to the miniAID-HA_3 (5′) and HXGPRT cassette (3′) integration were detected exclusively in the putative iKD line, while the wild-type fragment (wt) was amplified only in the untagged line. A ~ 4,000 bp fragment corresponding to the miniAID-HA_3 + HXGPRT cassette, and amplified with primers binding the wild-type sequence, was detected in the iKD line. L: DNA ladder (bp). Primers are listed in Table EV1.

G Marker-free strategy used for generating HA_3-miniAID-TgCRMPa strain. The integration of the tag at the N-terminus between residues Val69 and Leu70 (before the MAR/Kringle domain; Fig 5E) into the *TgCRMPa* locus is ensured by 207- and 265-bp-long homology regions (HR) flanking the tag in the synthetic gBlock, upon CRISPR-Cas9 activity (scissors). The arrows indicate the binding sites of the primers used in (H).

H Integration of the miniAID-HA_3 at the N-terminus of the *TgCRMPa* locus in the Tir-1 parental line was tested by PCR. The fragments corresponding to the HA_3 (5′) and the HA_3-miniAID (3′) integration were detected exclusively in the putative iKD line; the wild-type fragment (wt) was amplified in the untagged line (~ 1,556 bp) and iKD line (~ 1,026 bp, tag minus introns). L: DNA ladder (bp). Primers are listed in Table EV1.

I Immunofluorescence images of untagged and N-terminal and C-terminal miniAID-HA_3-TgCRMPa (iKD) intracellular tachyzoites. Parasites treated 24 h with IAA, as well as untreated (−IAA), were stained with anti-HA Abs. The nuclei (DNA) are stained with Hoechst. Shown are single focal planes.

J Representative images of lytic plaques formation in HFF monolayers infected with IAA-treated and untreated Tir-1 control and N-terminal and C-terminal miniAID-HA_3-TgCRMPa (iKD) lines.

K Immunofluorescence images of extracellular N-terminal and C-terminal miniAID-HA_3-TgCRMPa tachyzoites. Parasites were incubated with ionophore A23187 to induce artificial conoid extrusion, and stained with anti-HA Abs. TgCRMPa localization at the tip of the extruded conoid (arrow) is visible only in the C-terminally miniAID-HA_3-tagged TgCRMPa (lower panel). DNA is labeled by Hoechst. Single focal planes are shown. DIC: differential interference contrast.

L Quantification of the dot pattern for HA_3-miniAID-TgCRMPa (N-term) and TgCRMPa-miniAID-HA_3 (C-term) tachyzoites. TgCRMPa accumulation at the apical tip of extracellular parasites was measured upon incubation with ionophore A23187 to induce artificial conoid extrusion. Parasites were fixed and stained with anti-HA Abs and with (+ triton) or without (− triton) permeabilization. CRMPa signal at the apical dot is absent in non-permeabilized parasites, and it is robustly detected only in permeabilized parasites expressing C-terminally miniAID-HA_3-tagged TgCRMPa. No significant apical signal was detected for the control (untagged) or the N-terminally miniAID-HA_3-tagged TgCRMPa lines. Numbers are expressed as a percentage of parasites showing (dot) or lacking (no dot) the tip accumulation of TgCRMPa. The number of parasites (*n*) analyzed for each line is reported on the column tops.

M Marker-free strategy used for generating HA_3-TgCRMPa strain. The integration of the tag at the N-terminus between residues Thr600 and Asn601 (after the MAR/Kringle domain; Fig 5H) into the *TgCRMPa* locus is ensured by 200-bp-long homology regions (HR), flanking the tag in the synthetic gBlock upon CRISPR-Cas9 activity (scissors). The arrows indicate the binding sites of the primers used in (N).

N Integration of the triple HA at the N-terminus of the *TgCRMPa* locus was tested by PCR. The fragments corresponding to the 5′ and 3′ integration were detected exclusively in the putative HA_3-tagged line; and the wild-type fragment (wt) was amplified in the untagged line (~ 1,424 bp) and tagged line (~ 1,550 bp, containing linker+HA_3). L: DNA ladder (bp). Primers are listed in Table EV1.

O Immunofluorescence image of intracellular HA_3-TgCRMPa tachyzoites. Parasites were stained with anti-HA Abs and DNA is labeled by Hoechst. Single focal planes are shown. DIC, differential interference contrast.

Source data are available online for this figure.

 © 2022 The Authors

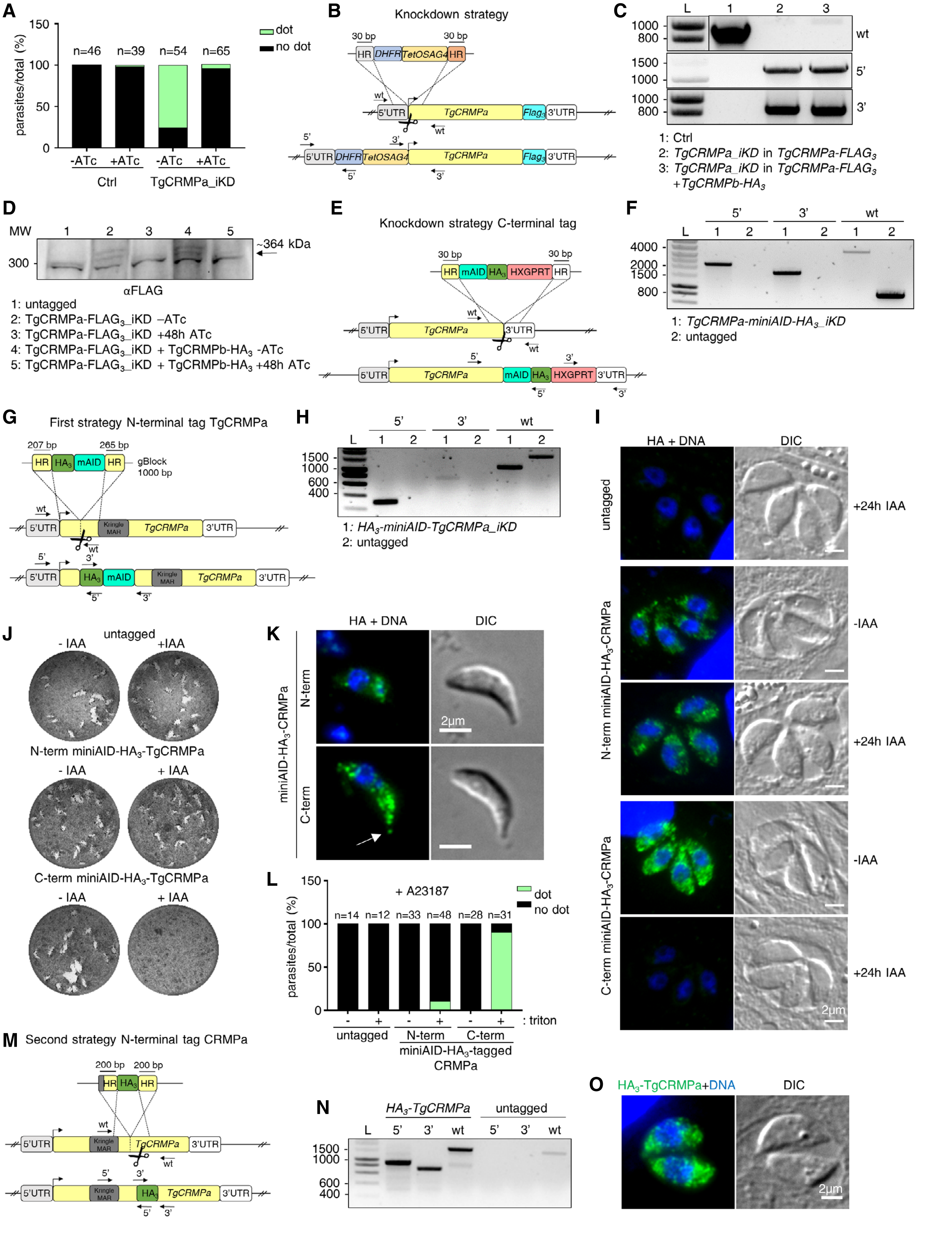
A
parasites/total (%)
dot
no dot
n=46 n=39 n=54 n=65
-ATc +ATc -ATc +ATc
Ctrl TgCRMPa_iKD
B
Knockdown strategy
30 bp 30 bp
HR DHFR TetOSAG4 HR
5'UTR TgCRMPa Flag3 3'UTR
C
L 1 2 3
wt
5'
3'
1: Ctrl
2: TgCRMPa_iKD in TgCRMPa-FLAG3
3: TgCRMPa_iKD in TgCRMPa-FLAG3 +TgCRMPb-HA3
D
MW 1 2 3 4 5
~364 kDa
αFLAG
1: untagged
2: TgCRMPa-FLAG3_iKD –ATc
3: TgCRMPa-FLAG3_iKD +48h ATc
4: TgCRMPa-FLAG3_iKD + TgCRMPb-HA3 -ATc
5: TgCRMPa-FLAG3_iKD + TgCRMPb-HA3 +48h ATc
E
Knockdown strategy C-terminal tag
HR mAID HA3 HXGPRT HR
F
1: TgCRMPa-miniAID-HA3_iKD
2: untagged
G
First strategy N-terminal tag TgCRMPa
207 bp 265 bp
gBlock 1000 bp
H
1: HA3-miniAID-TgCRMPa_iKD
2: untagged
I
HA + DNA DIC
untagged
N-term miniAID-HA3-CRMPa
C-term miniAID-HA3-CRMPa
+24h IAA
-IAA
J
untagged
- IAA +IAA
N-term miniAID-HA3-TgCRMPa
C-term miniAID-HA3-TgCRMPa
K
miniAID-HA3-CRMPa
N-term
C-term
2µm
L
+ A23187
n=14 n=12 n=33 n=48 n=28 n=31
: triton
untagged N-term C-term
miniAID-HA3-tagged CRMPa
M
Second strategy N-terminal tag CRMPa
200 bp 200 bp
N
HA3-TgCRMPa untagged
L 5' 3' wt 5' 3' wt
O
HA3-TgCRMPa+DNA DIC

Figure EV4.

Figure EV5. CRMPs and Nd6 co-localize at the exocytic site in extracellular *Toxoplasma gondii* (related to Fig 6).

A Strategy for TY_2 tagging of TgNd6 in TgCRMPa-HA_3 and TgCRMPb-HA_3 lines. To generate a C-terminal TY_2-fusion of TgNd6, a DNA fragment was amplified from a donor vector containing the TY_2 tag and the drug resistance cassette (DHFR). Primers to amplify the DNA fragment were designed to contain 30-bp-long stretches (HR) homologous to TgND6 regions flanking the insertion site for the epitope tag. Upon CRISPR-cas9 cut (scissors), the PCR-amplified DNA fragment efficiently recombines into the targeted endogenous locus. The arrows indicate the binding sites of the primers used in (B).

B Integration of the TY_2 tag and DHFR cassette at the C-terminus of *TgND6* locus was tested by PCR. Genomic DNAs from an untagged line and clonal populations for TgNd6-TY_2 + TgCRMPa-HA_3 and TgNd6-TY_2 + TgCRMPb-HA_3 lines were amplified with primers binding to the 3′ C-terminus and 3′UTR of *TgNd6*, and also in pairwise combination with primers binding the TY_2 and DHFR sequences, respectively. The fragments corresponding to the TY_2 tag (5′) and the resistance cassette (3′) were correctly amplified in the putative tagged lines, indicating that they were efficiently integrated at the *TgNd6* locus. As expected, the wild-type fragment for *TgNd6* (wt) was detected only in the untagged line. L: DNA ladder (bp). Primers are listed in Table EV1.

C Whole-cell lysates from untagged and TgNd6-TY_2 + TgCRMPa-HA_3 and TgNd6-TY_2 + TgCRMPb-HA_3 parasites were immunoblotted with anti-TY Abs to detect tagged Nd6. A band around the expected size (~ 195 kDa) for TgNd6-TY_2 was observed exclusively for the tagged lines. MW: molecular weight standards.

D Immunofluorescence images of intracellular (upper and middle panels) and extracellular (lower panel) tachyzoites from untagged and TgCRMPb-HA_3 + TgNd6-TY_2 lines. Extracellular parasites were incubated with host cell monolayers for 2 min prior to fixation. Parasites were stained with anti-HA and anti-TY Abs to label CRMPb and Nd6, respectively. Nd6, but not CRMPb, accumulates at the tachyzoite apex in intracellular parasites (arrowheads), while both proteins localize at the tip of the extruded conoid in extracellular parasites (arrows). DNA is labeled by Hoechst. Single focal planes are shown. DIC, differential interference contrast.

E Integration of the HA_3 tag and CAT cassette at the C-terminus of *TgCRMPa* and *TgCRMPb* genes in TgNd9_iKD line was tested by PCR as in Fig EV2A and B. The fragments corresponding to the HA_3 tag (5′) and the resistance cassette (3′) were correctly amplified in the putative tagged lines, indicating that they were efficiently integrated at the TgCRMPs loci. As expected, the wild-type fragment of each gene (wt) was detected only in the untagged line. L: DNA ladder. Primers are listed in Table EV1.

F Depletion of *TgNd9* transcripts was assessed by RT–PCR for the experiment shown in Fig 6D. Total RNAs from TgCRMPa-HA_3 and TgCRMPb-HA_3 expressed in TgNd9_iKD (minus epitope tag) parasites and parental line were subjected to reverse transcription and PCR amplified with primers binding *TgNd9* transcripts. *TgGAPDH* was used as housekeeping gene. *TgNd9* transcripts strongly decreased upon 72 h ATc treatment (+ATc). L: DNA ladder (L). Primers are listed in Table EV1.

G Depletion of TgNd9 proteins in the lines used for the experiment in Fig 6D was also assessed by quantifying the defect in the invasion of ATc-treated TgNd9_iKD parasites expressing TgCRMPa-HA_3 and TgCRMPb-HA_3 versus untreated. The values are reported as percentages of the number of invading/intracellular and extracellular parasites over the total number of parasites. The number of fields (f) analyzed for each line is reported on the column tops.

H Immunofluorescence images of intracellular and extracellular tachyzoites from TgCRMPb-HA_3 + TgCRMPa-TY_2 line. Extracellular parasites were incubated with host cell monolayers for 2 min prior to fixation. Parasites were stained with anti-HA and anti-TY Abs to label CRMPb and CRMPa, respectively. Both proteins localize at the tip of the extruded conoid in extracellular parasites (arrows) and show partial overlap within the parasite cytosol. An untagged line was used to estimate the background noise. DNA is labeled by Hoechst. Single focal planes are shown. DIC, differential interference contrast.

I Integration of the TY_2 tag and DHFR cassette at the C-terminus of the *TgCRMPa* locus in TgCRMPb-HA_3 line was tested by PCR as described in (B) for TgNd6-TY_2. The fragments corresponding to the TY_2 tag (5′) and the resistance cassette (3′) were correctly amplified in the putative tagged line, indicating that they were efficiently integrated at the *TgCRMPa* locus. As expected, the wild-type fragment for *TgCRMPa* (wt) was detected only in the untagged line. L: DNA ladder (bp). Primers are listed in Table EV1.

J Whole-cell lysates from TgCRMPb-HA_3 and TgCRMPb-HA_3 + TgCRMPa-TY_2 parasites were immunoblotted with anti-TY Abs to detect tagged CRMPa. A band around the expected size (~ 348 kDa) for TgCRMPa-TY_2 together with the processed form were observed exclusively for the tagged line. MW: molecular weight standards.

Source data are available online for this figure.

 © 2022 The Authors

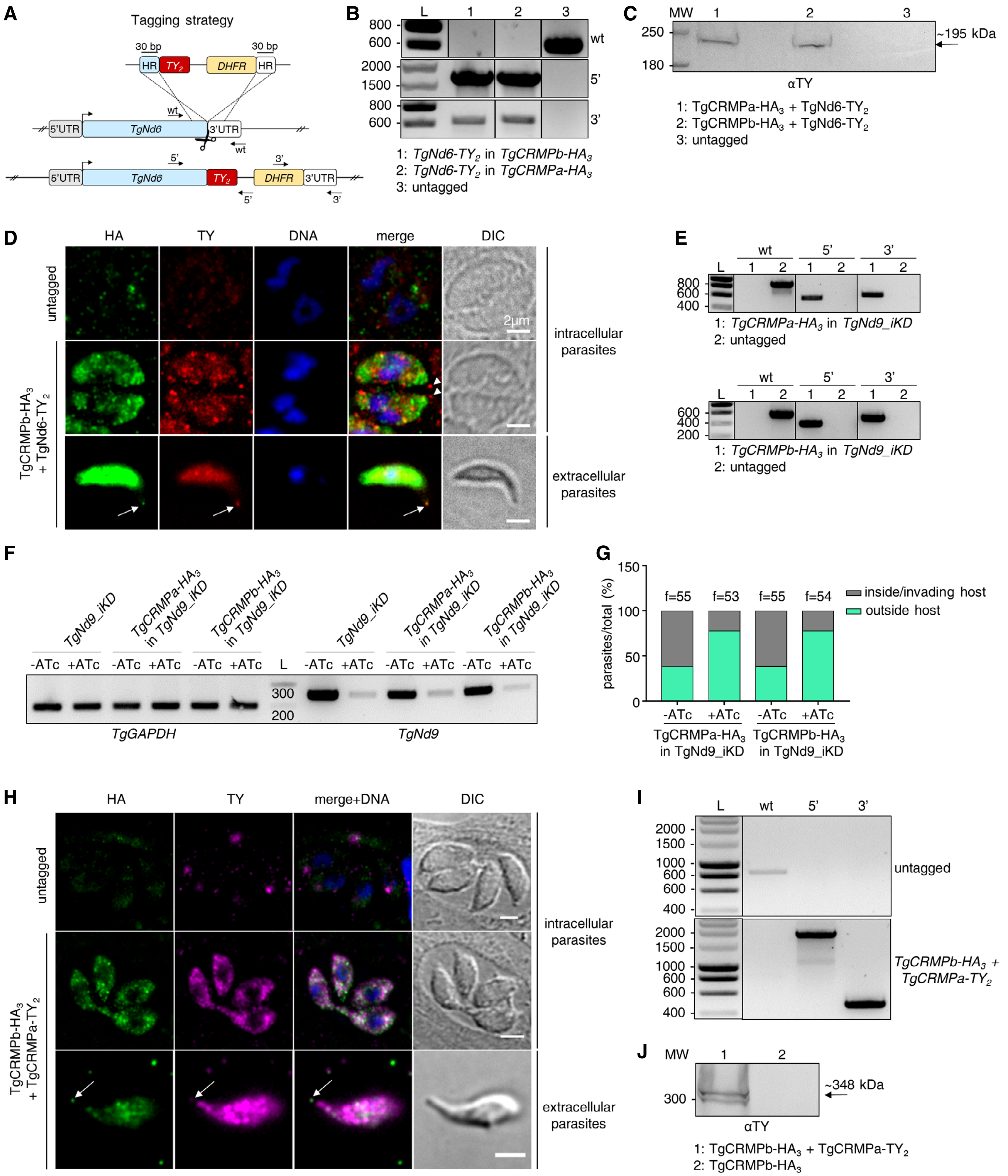

A
Tagging strategy
30 bp
30 bp
HR
TY2
DHFR
HR
5'UTR
TgNd6
3'UTR
wt
wt
5'
3'
TY2
B
L 1 2 3
800
600
2000
1500
800
600
wt
5'
3'
1: TgNd6-TY2 in TgCRMPb-HA3
2: TgNd6-TY2 in TgCRMPa-HA3
3: untagged
C
MW 1 2 3
250
180
~195 kDa
αTY
1: TgCRMPa-HA3 + TgNd6-TY2
2: TgCRMPb-HA3 + TgNd6-TY2
3: untagged
D
HA TY DNA merge DIC
untagged
TgCRMPb-HA3 + TgNd6-TY2
2μm
intracellular parasites
extracellular parasites
E
wt 5' 3'
L 1 2 1 2 1 2
800
600
400
1: TgCRMPa-HA3 in TgNd9_iKD
2: untagged
600
400
200
1: TgCRMPb-HA3 in TgNd9_iKD
2: untagged
F
TgNd9_iKD
TgCRMPa-HA3 in TgNd9_iKD
TgCRMPb-HA3 in TgNd9_iKD
-ATc +ATc -ATc +ATc -ATc +ATc L -ATc +ATc -ATc +ATc -ATc +ATc
300
200
TgGAPDH
TgNd9
G
150
100
50
0
parasites/total (%)
f=55 f=53 f=55 f=54
inside/invading host
outside host
-ATc +ATc -ATc +ATc
TgCRMPa-HA3 in TgNd9_iKD
TgCRMPb-HA3 in TgNd9_iKD
H
HA TY merge+DNA DIC
untagged
TgCRMPb-HA3 + TgCRMPa-TY2
intracellular parasites
extracellular parasites
I
L wt 5' 3'
2000
1500
1000
600
600
400
untagged
TgCRMPb-HA3 + TgCRMPa-TY2
J
MW 1 2
300
~348 kDa
αTY
1: TgCRMPb-HA3 + TgCRMPa-TY2
2: TgCRMPb-HA3

Figure EV5.

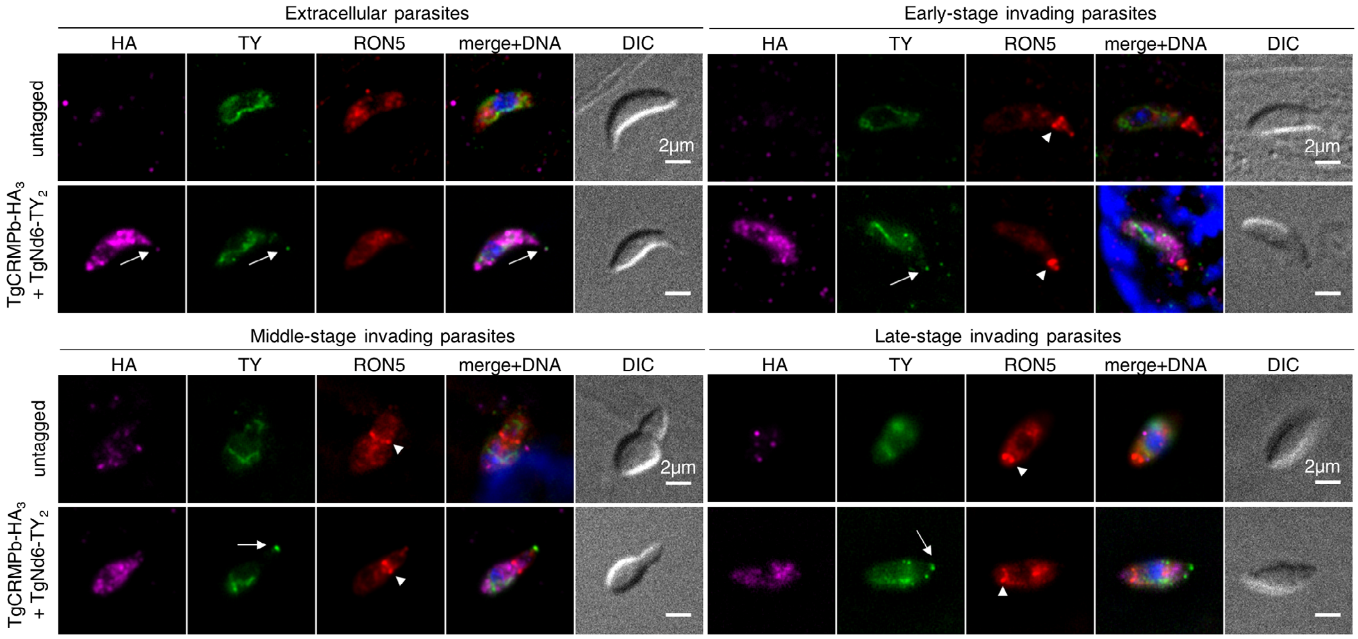

Figure EV6. CRMP and Nd complexes show different dynamics at the exocytic site in *Toxoplasma gondii* (related to Fig 6).

Immunofluorescence images of extracellular parasites and parasites in early, middle, and late stages of host cell invasion. Parasites co-expressing TgCRMPb-HA_3 with TgNd6-TY_2 were incubated with host cell monolayers and stained as in Fig 6E. Untagged parasites were treated in parallel. In contrast with TgNd6 (arrow), the apical accumulation of TgCRMPb observed in extracellular parasites disappears upon entering the host and remains undetected for the entire process. The moving junction is indicated by the arrowhead. Non-specific anti-TY labeling of mitochondria was detected for both untagged and tagged lines. DIC, differential interference contrast. Single focal planes are shown.

 © 2022 The Authors
