# Supplementary material for: An apical membrane complex for triggering rhoptry exocytosis and invasion in Toxoplasma
Source: EMBO J. 2022 Oct 17;41(22):e111158. doi: 10.15252/embj.2022111158 (PMC9670195; doi:10.15252/embj.2022111158)

Related to figure EV3

Figure EV3D, left, middle, right panels

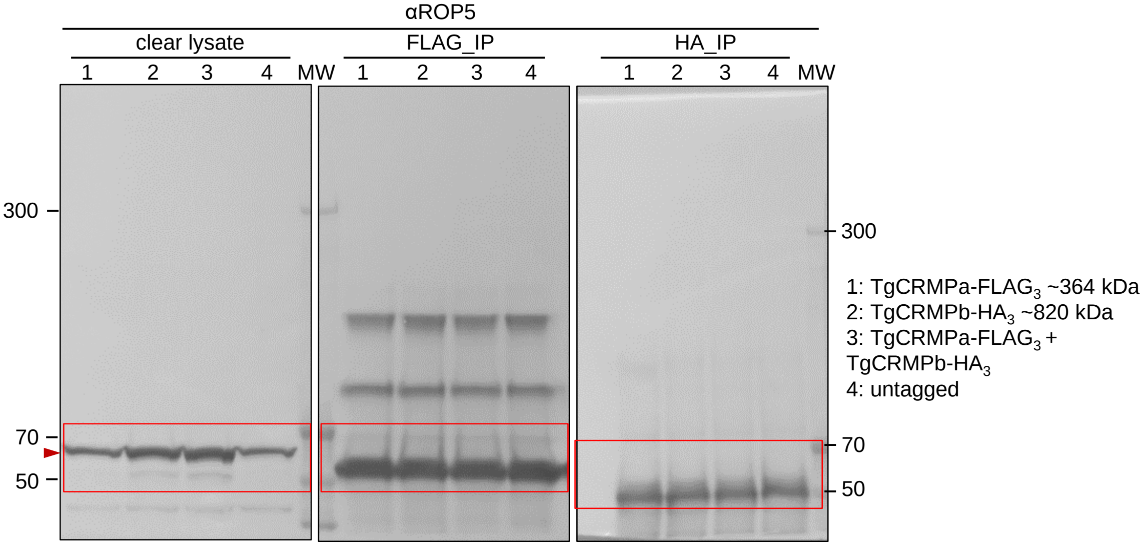

Figure EV3A

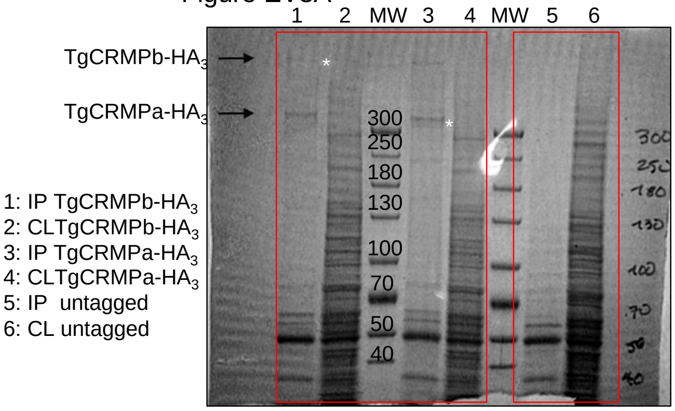

Figure EV3C

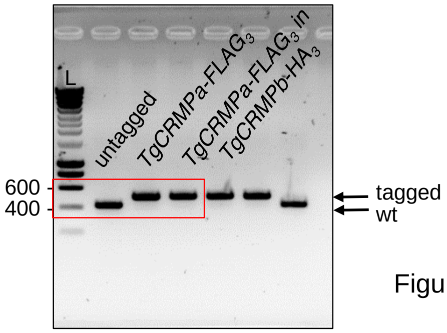

Figure EV3FH

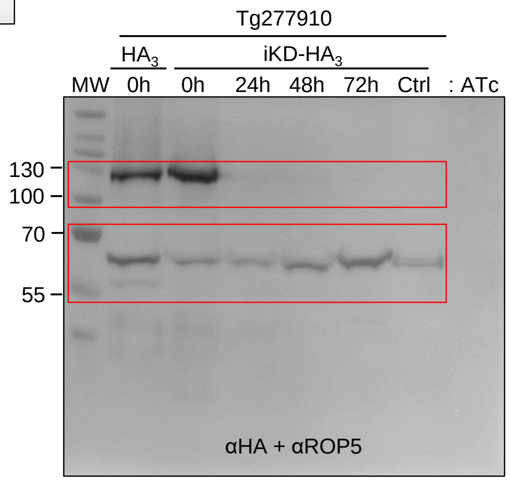

Figure EV3F

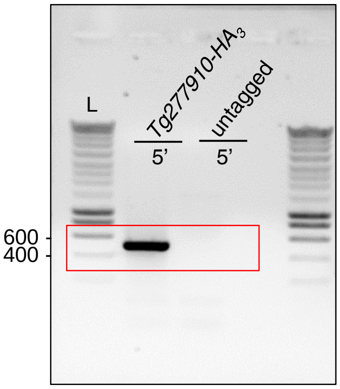

Figure EV3G, left and right panels

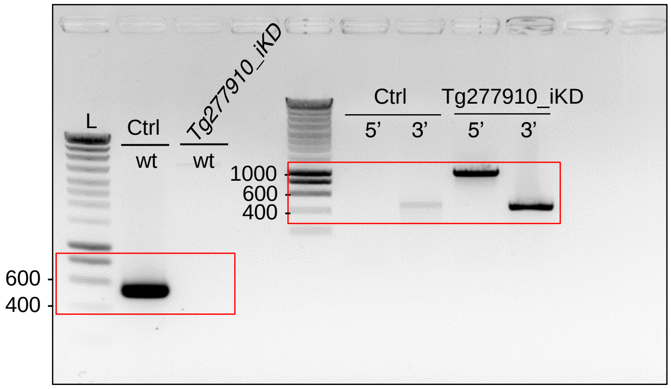

Supplement: Supplementary file 7 — Source Data for Expanded View [file EMBJ-41-e111158-s003.zip › Source_data_Figure EV3.pdf]
