# Supplementary material for: An apical membrane complex for triggering rhoptry exocytosis and invasion in Toxoplasma
Source: EMBO J. 2022 Oct 17;41(22):e111158. doi: 10.15252/embj.2022111158 (PMC9670195; doi:10.15252/embj.2022111158)

Related to figure EV1

Figure EV1B, upper panel

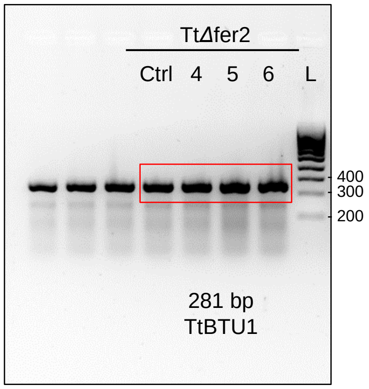

Figure EV1B, lower panel

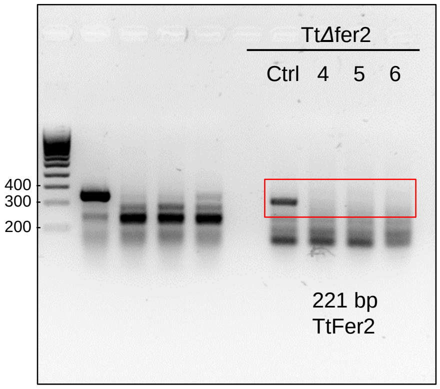

Figure EV1C

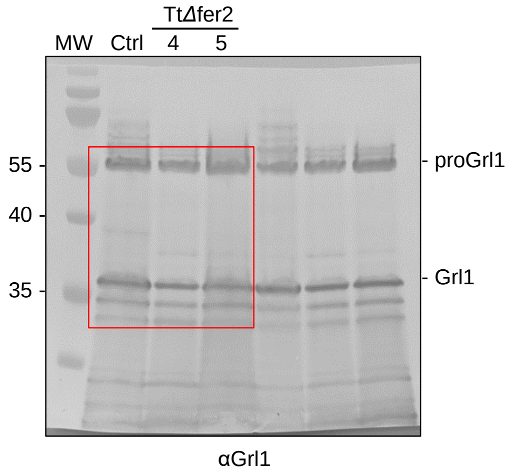

Figure EV1E, upper panel

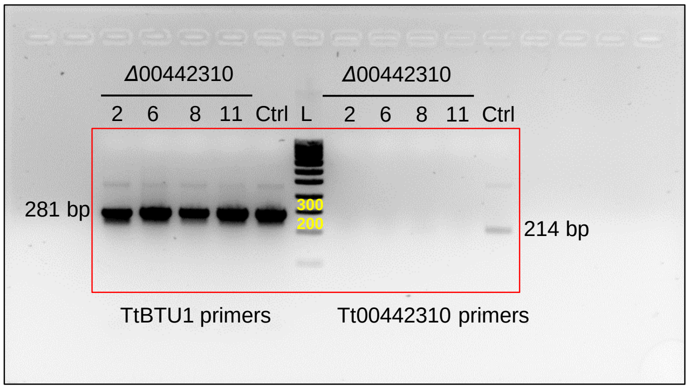

Figure EV1E, lower panel

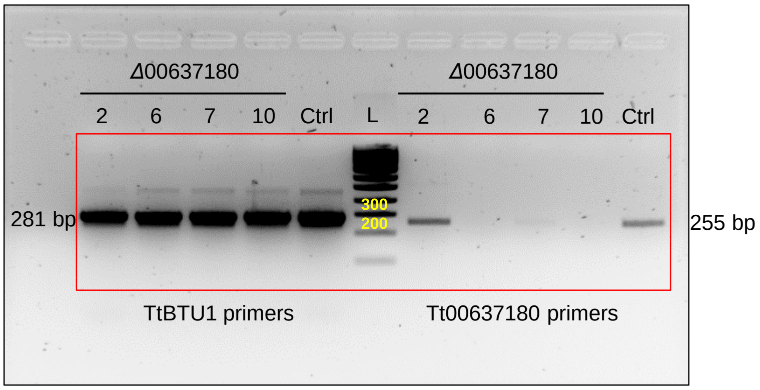

Figure EV1F

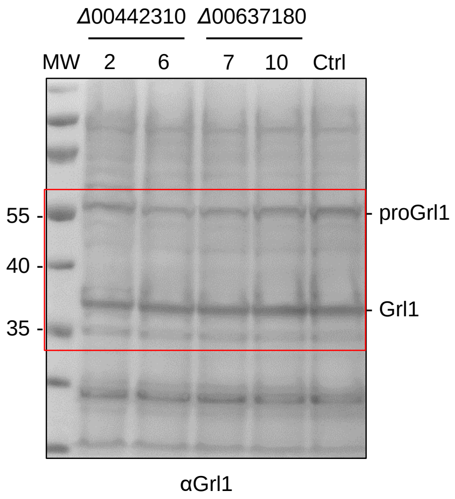

Supplement: Supplementary file 7 — Source Data for Expanded View [file EMBJ-41-e111158-s003.zip › Source_data_Figure EV1.pdf]
