# Supplementary material for: An apical membrane complex for triggering rhoptry exocytosis and invasion in Toxoplasma
Source: EMBO J. 2022 Oct 17;41(22):e111158. doi: 10.15252/embj.2022111158 (PMC9670195; doi:10.15252/embj.2022111158)

Related to figure EV5

Figure EV5B

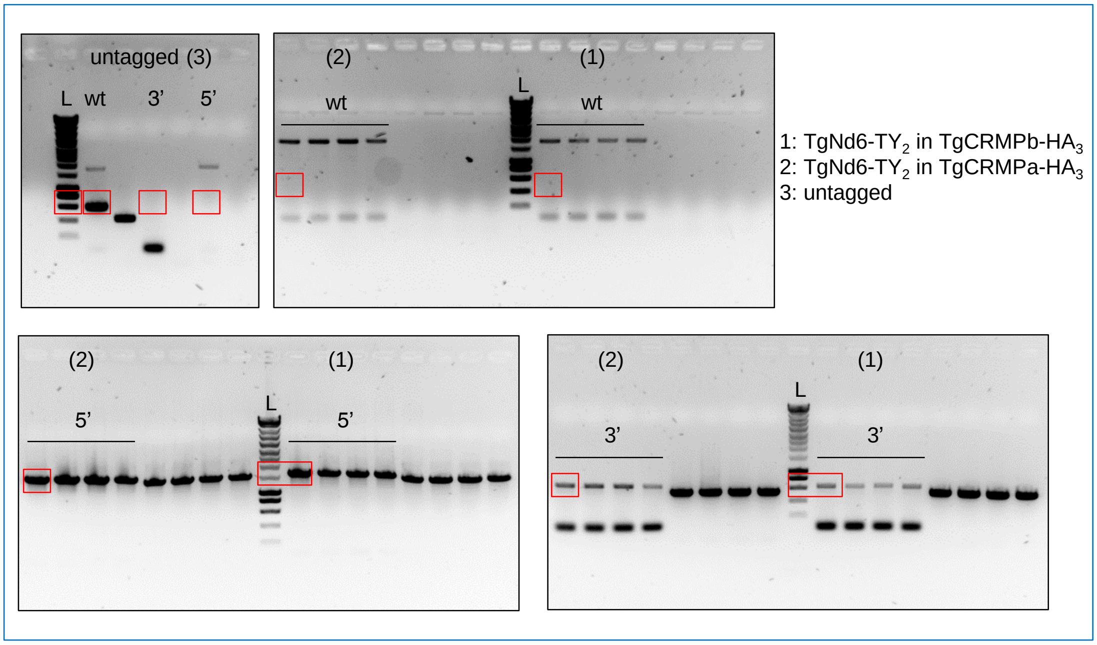

Figure EV5C

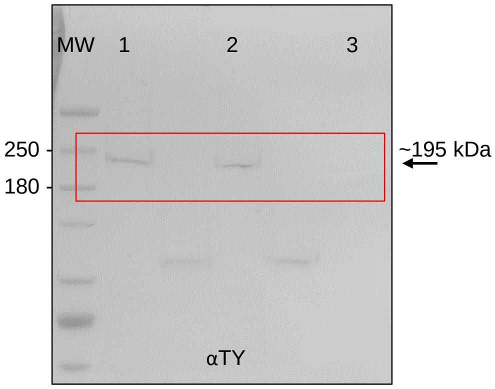

1: TgCRMPa-HA$_3$ + TgNd6-TY$_2$
2: TgCRMPb-HA$_3$ + TgNd6-TY$_2$
3: untagged

Figure EV5E, upper panel

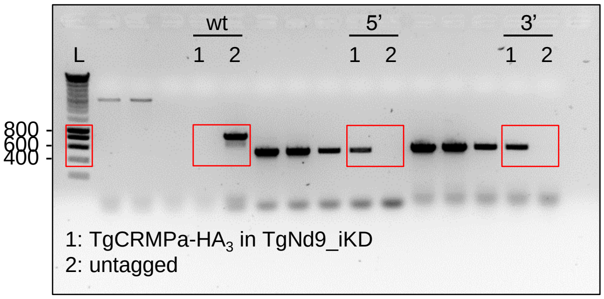

Figure EV5E, lower panel

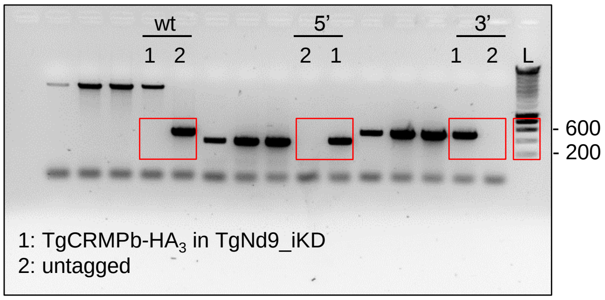

Figure EV5F

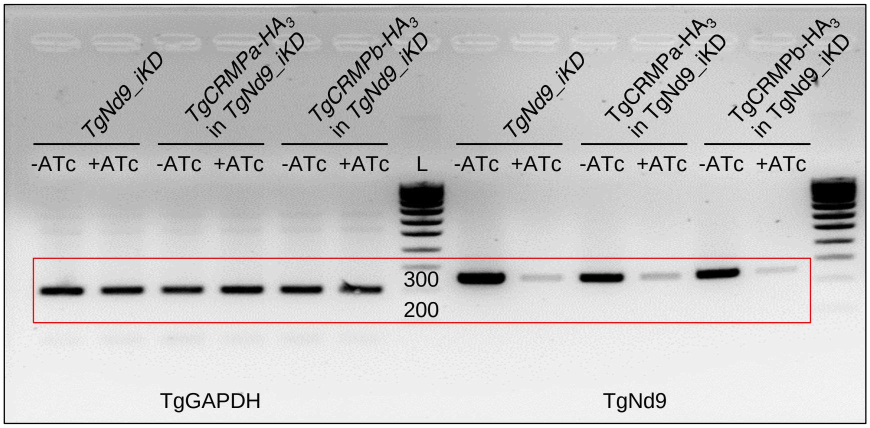

Figure EV5J

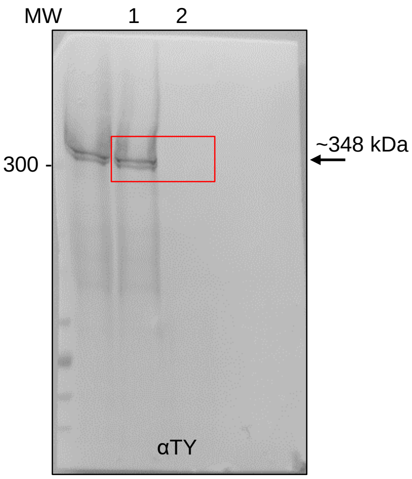

1: TgCRMPb-HA$_3$ + TgCRMPa-TY$_2$
2: TgCRMPb-HA$_3$

Figure EV5I, upper and lower panels

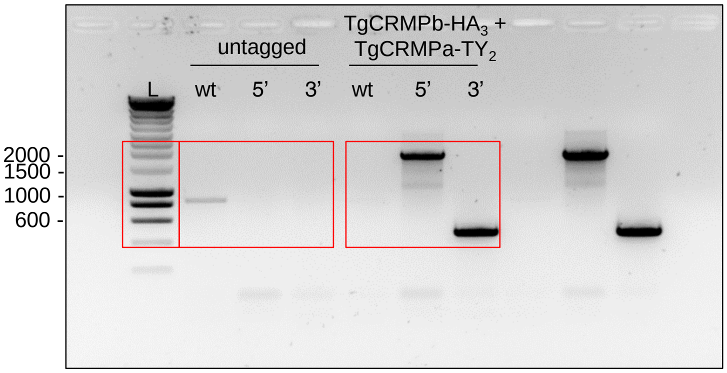

Supplement: Supplementary file 7 — Source Data for Expanded View [file EMBJ-41-e111158-s003.zip › Source_data_Figure EV5.pdf]
