# Supplementary material for: An apical membrane complex for triggering rhoptry exocytosis and invasion in Toxoplasma
Source: EMBO J. 2022 Oct 17;41(22):e111158. doi: 10.15252/embj.2022111158 (PMC9670195; doi:10.15252/embj.2022111158)

Related to figure EV4

Figure EV4D

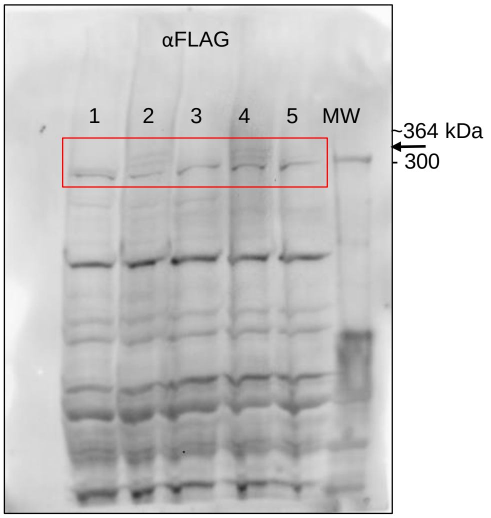

1: untagged
2: TgCRMPa-FLAG$_3$_iKD –ATc
3: TgCRMPa-FLAG$_3$_iKD +48h ATc
4: TgCRMPa-FLAG$_3$_iKD + TgCRMPb-HA$_3$ -ATc
5: TgCRMPa-FLAG$_3$_iKD + TgCRMPb-HA$_3$ +48h ATc

Figure EV4C, upper, lower panels

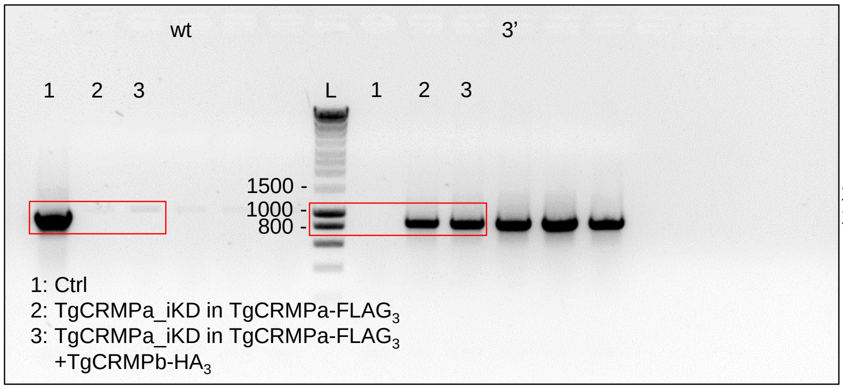

Figure EV4C, middle panel

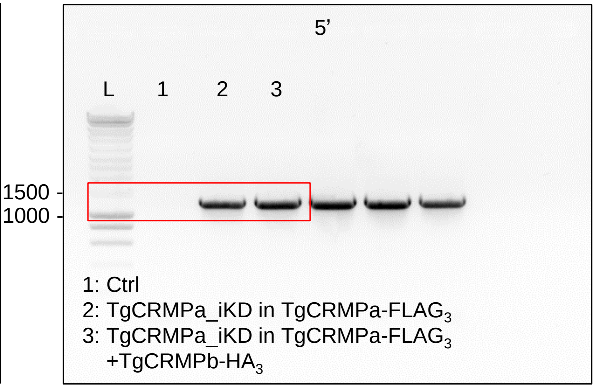

Figure EV4F

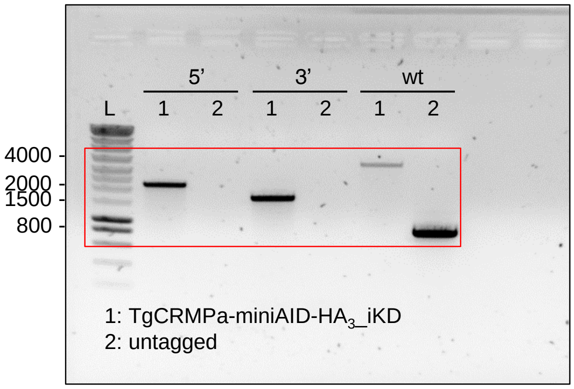

Figure EV4H

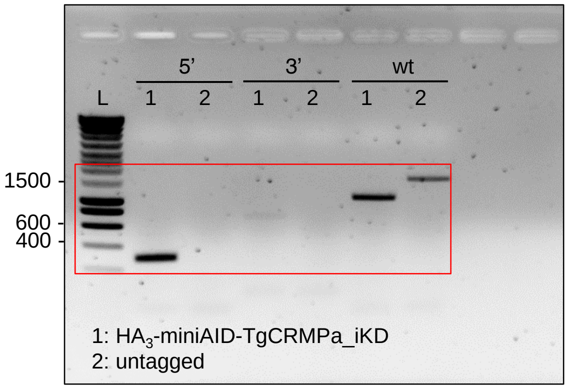

Figure EV4N

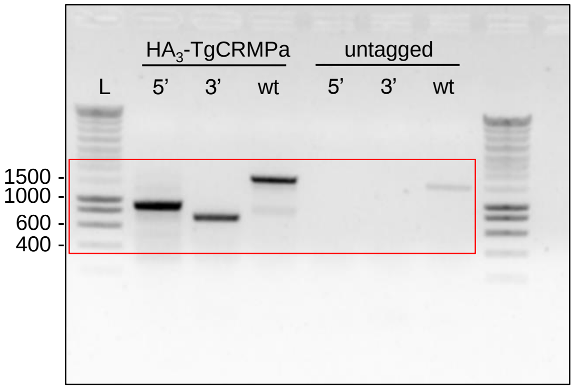

Supplement: Supplementary file 7 — Source Data for Expanded View [file EMBJ-41-e111158-s003.zip › Source_data_Figure EV4.pdf]
