# Supplementary figures and images for: An apical membrane complex for triggering rhoptry exocytosis and invasion in Toxoplasma
Source: EMBO J. 2022 Oct 17;41(22):e111158. doi: 10.15252/embj.2022111158 (PMC9670195; doi:10.15252/embj.2022111158)

Figure EV2B, upper panel

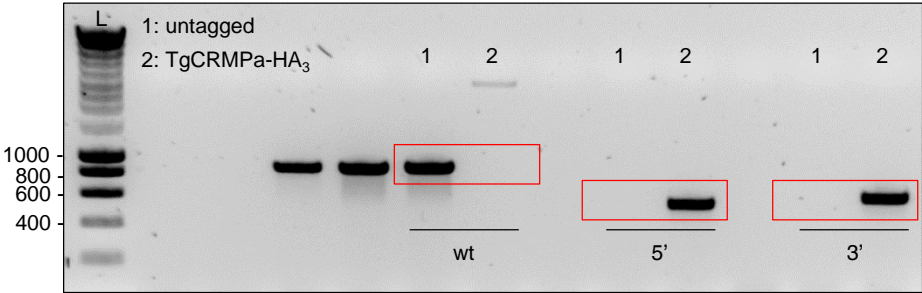

Figure EV2B, lower panel

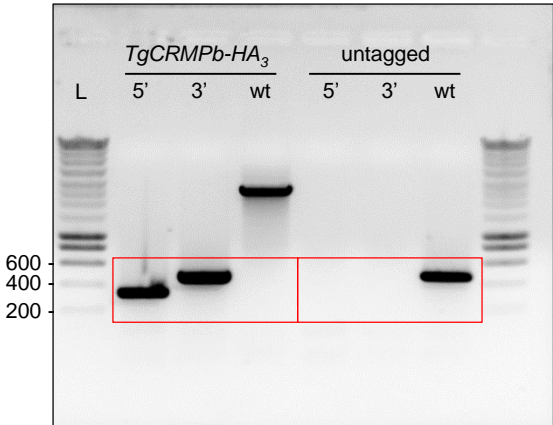

Figure EV2D, upper panel

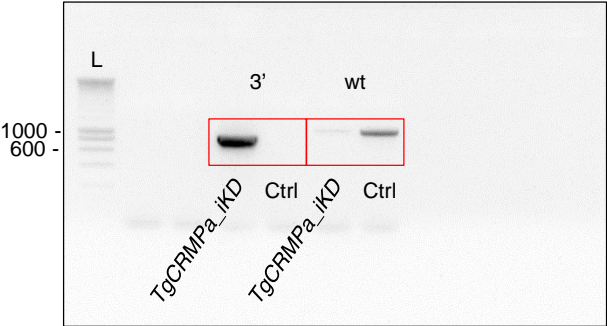

Figure EV2D, lower panel

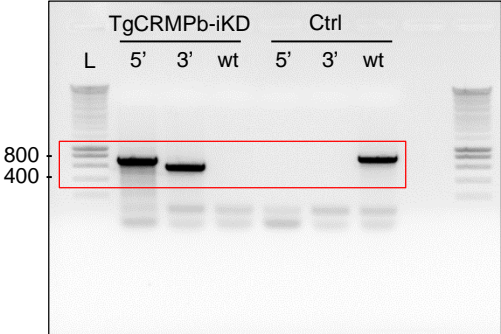

Supplement: Supplementary file 7 — Source Data for Expanded View [file EMBJ-41-e111158-s003.zip › Source_data_Figure EV2.pdf]

Related to figure 2

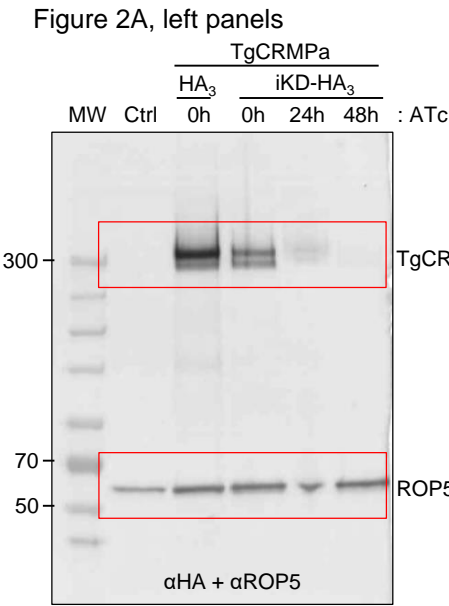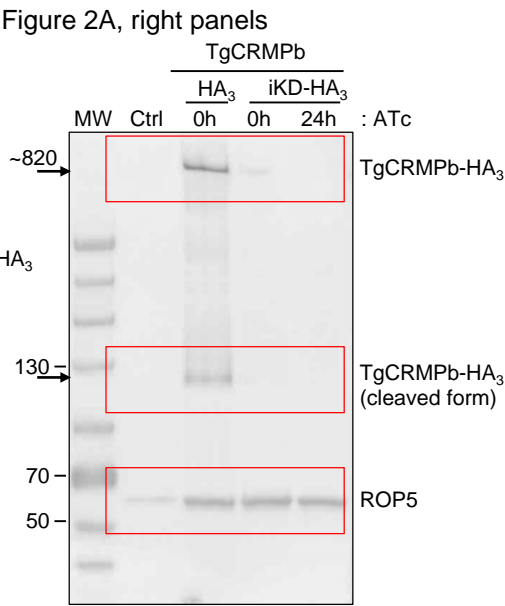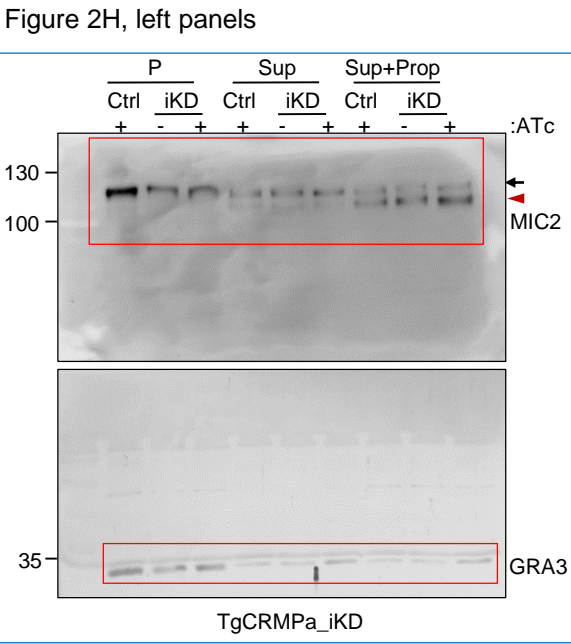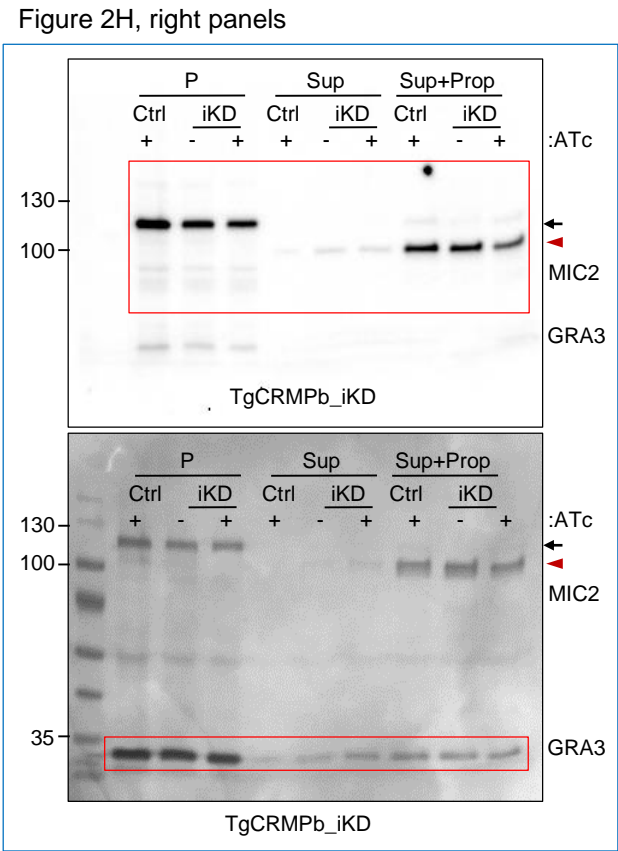

Supplement: Supplementary file 8 — Source Data for Figure 2 [file EMBJ-41-e111158-s002.pdf]

Related to figure 3

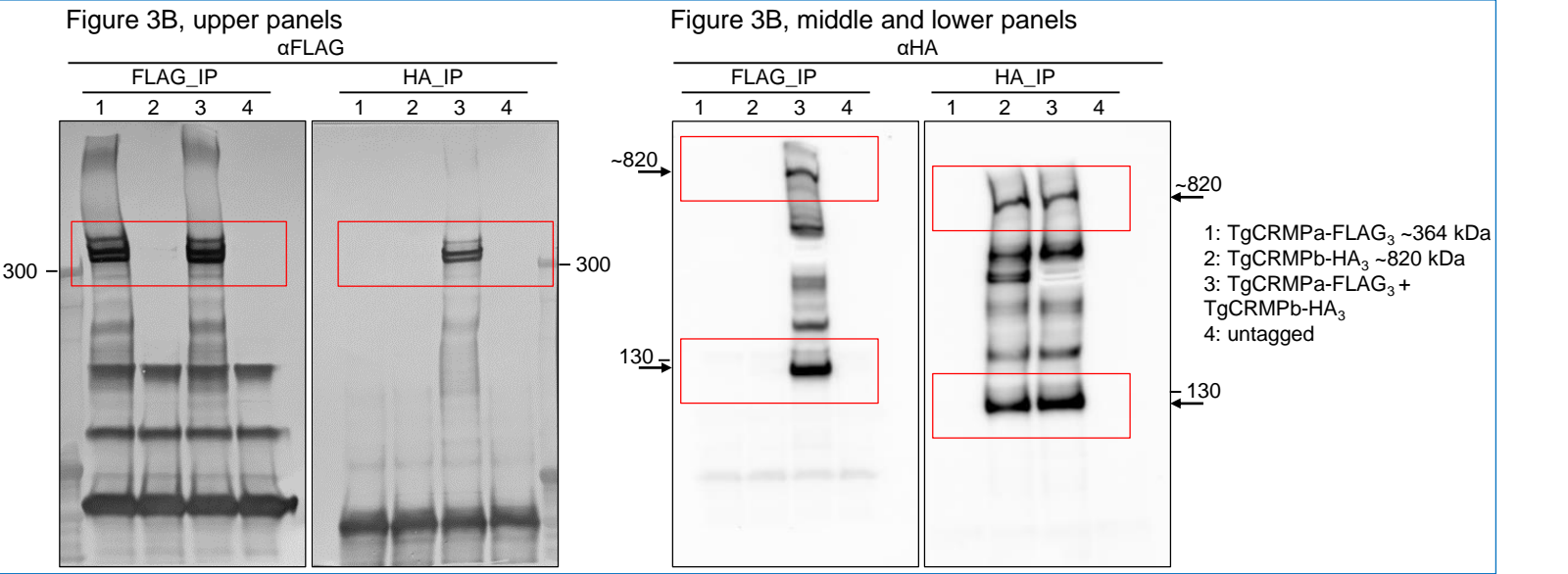

Figure 3G, upper and lower panels

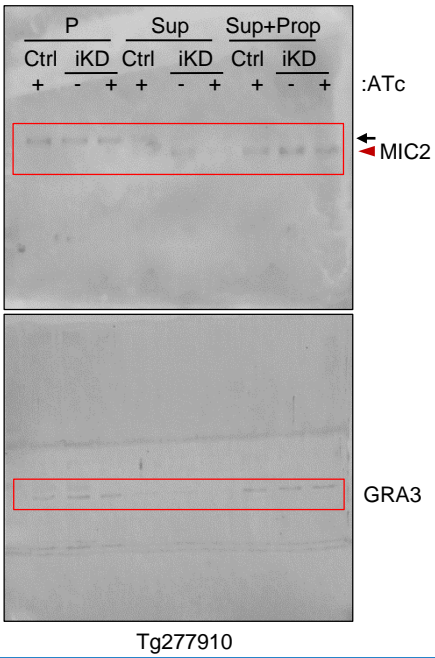

Supplement: Supplementary file 9 — Source Data for Figure 3 [file EMBJ-41-e111158-s012.pdf]

Figure 5G

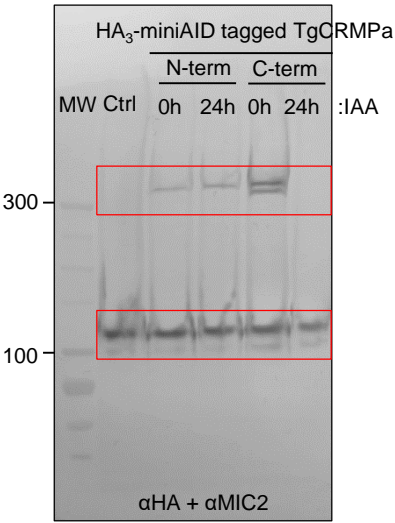

Figure 5I

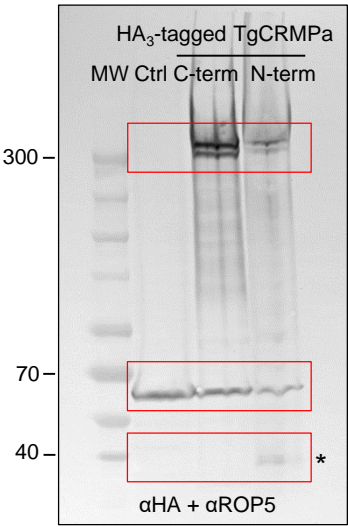

Supplement: Supplementary file 10 — Source Data for Figure 5 [file EMBJ-41-e111158-s004.pdf]
