# Supplementary material for: An apical membrane complex for triggering rhoptry exocytosis and invasion in Toxoplasma
Source: EMBO J. 2022 Oct 17;41(22):e111158. doi: 10.15252/embj.2022111158 (PMC9670195; doi:10.15252/embj.2022111158)
Supplement: Supplementary file 11 — PDF+ [file EMBJ-41-e111158-s001.pdf]

Article

SOURCE DATA TRANSPARENT PROCESS OPEN ACCESS

THE EMBO JOURNAL

An apical membrane complex for triggering rhoptry exocytosis and invasion in *Toxoplasma*

Daniela Sparvoli[1,†], Jason Delabre[1,†], Diana Marcela Penarete-Vargas[1,‡], Shrawan Kumar Mageswaran[2,‡], Lev M Tsypin[3,§], Justine Heckendorn[1], Liam Theveny[2], Marjorie Maynadier[1], Marta Mendonça Cova[1], Laurence Berry-Sterkers[1], Amandine Guérin[4], Jean-François Dubremetz[1], Serge Urbach[5], Boris Striepen[4], Aaron P Turkewitz[3], Yi-Wei Chang[2] & Maryse Lebrun[1,*]

Abstract

Apicomplexan parasites possess secretory organelles called rhoptries that undergo regulated exocytosis upon contact with the host. This process is essential for the parasitic lifestyle of these pathogens and relies on an exocytic machinery sharing structural features and molecular components with free-living ciliates. However, how the parasites coordinate exocytosis with host interaction is unknown. Here, we performed a *Tetrahymena*-based transcriptomic screen to uncover novel exocytic factors in Ciliata and conserved in Apicomplexa. We identified membrane-bound proteins, named CRMPs, forming part of a large complex essential for rhoptry secretion and invasion in *Toxoplasma*. Using cutting-edge imaging tools, including expansion microscopy and cryo-electron tomography, we show that, unlike previously described rhoptry exocytic factors, TgCRMPs are not required for the assembly of the rhoptry secretion machinery and only transiently associate with the exocytic site—prior to the invasion. CRMPs and their partners contain putative host cell-binding domains, and CRMPa shares similarities with GPCR proteins. Collectively our data imply that the CRMP complex acts as a host–molecular sensor to ensure that rhoptry exocytosis occurs when the parasite contacts the host cell.

Keywords apicomplexa; ciliates; CRMP; rhoptry; secretion

Subject Categories Membranes & Trafficking; Microbiology, Virology & Host Pathogen Interaction

DOI 10.15252/embj.2022111158 | Received 14 March 2022 | Revised 13 September 2022 | Accepted 19 September 2022 | Published online 17 October 2022

The EMBO Journal (2022) 41: e111158

Introduction

Apicomplexan parasites can cause life-threatening diseases including malaria, cryptosporidiosis, and toxoplasmosis. They are obligate intracellular organisms that invade and subvert functions of diverse host cells by releasing multiple adhesins, perforins, and effectors from three different secretory organelles: micronemes, rhoptries, and dense granules (Lebrun *et al*, 2020). The content of rhoptries is secreted directly into the host cell (Gilbert *et al*, 2007; Besteiro *et al*, 2009), typically at the onset of host cell contact (Carruthers & Sibley, 1997; Riglar *et al*, 2011). The signaling pathways that mediate rhoptry discharge are unknown, but they might depend on the initial secretion of microneme proteins (Kessler *et al*, 2008; Singh *et al*, 2010). Upon injection into the host cell, rhoptry proteins facilitate invasion by establishing a structure called the moving junction (MJ), which anchors the parasite invasion machinery into the host cell cortex (Besteiro *et al*, 2011; Guerin *et al*, 2017). Rhoptry proteins also contribute to the formation of the parasitophorous vacuole (Ghosh *et al*, 2017) and play key roles in subverting host immune responses (Kemp *et al*, 2012; Hakimi *et al*, 2017). How rhoptry content is delivered into the host cell cytoplasm has been a vexing question for decades. Delivery requires docking and fusion of the organelle with the parasite plasma membrane (PPM); this process of exocytosis is coupled with the translocation of rhoptry content across the host plasma membrane (HPM). The latter likely involves the formation of a pore at the junction between the PPM and HPM (Nichols *et al*, 1983; Suss-Toby *et al*, 1996; Dubremetz, 1998; Hanssen *et al*, 2013; Burrell *et al*, 2021), but its nature and composition are unknown. Excitingly, recent studies revealed new insights into the structure and molecular players essential for the exocytic step (Suarez *et al*, 2019; Aquilini *et al*, 2021; Mageswaran

1 Laboratory of Pathogen Host Interactions, UMR 5235 CNRS, Université de Montpellier, Montpellier, France
2 Department of Biochemistry and Biophysics, Perelman School of Medicine, University of Pennsylvania, Philadelphia, PA, USA
3 Department of Molecular Genetics and Cell Biology, University of Chicago, Chicago, IL, USA
4 Department of Pathobiology, School of Veterinary Medicine, University of Pennsylvania, Philadelphia, PA, USA
5 IGF, Université de Montpellier, CNRS, INSERM, Montpellier, France
*Corresponding author. Tel: +33 04 67 14 37 45; E-mail: maryse.lebrun@umontpellier.fr
†These authors contributed equally to this work
‡These authors contributed equally to this work
§Present address: Division of Biology and Biological Engineering, California Institute of Technology, Pasadena, CA, USA

© 2022 The Authors. Published under the terms of the CC BY 4.0 license.

et al, 2021; Martinez *et al*, 2022). Rhoptry exocytosis relies on the proper assembly of a "rosette" of eight particles embedded in the PPM at the apex of the parasite (Aquilini *et al*, 2021). A similar rosette is present at the exocytic site of ciliate secretory organelles known as trichocysts in *Paramecium tetraurelia* and mucocysts in *Tetrahymena thermophila* (Satir *et al*, 1972; Plattner *et al*, 1973), and its presence is a firm requirement for the release of organelle content (Beisson *et al*, 1976). Cryo-electron tomography (Cryo-ET) of the apical tips of *Toxoplasma*, *Cryptosporidium*, and *Plasmodium* zoites revealed the rosette to be part of an elaborate machinery named Rhoptry Secretory Apparatus (RSA; Aquilini *et al*, 2021; Mageswaran *et al*, 2021; Martinez *et al*, 2022). This complex molecular machine connects the rhoptry to the PPM via an intermediate apical vesicle (AV). A group of Alveolata-restricted "non-discharge" proteins (Nd6, Nd9, NdP1, and NdP2) is required for the formation of the rosette in both Ciliata and Apicomplexa (Froissard *et al*, 2001; Gogendeau *et al*, 2005; Aquilini *et al*, 2021), demonstrating a conserved mechanism for exocytic fusion in Alveolata (reviewed in Sparvoli & Lebrun, 2021). However, several aspects of rhoptry secretion remain unknown, including the exact function of Nd proteins in this process, and how rhoptry discharge is regulated and triggered by host cell contact to inject content inside the host.

Here, we extend the use of ciliate models, specifically *Tetrahymena thermophila*, to further uncover the mechanism of rhoptry secretion. *Tetrahymena* possesses hundreds of mucocysts concentrated at the plasma membrane which are capable of rapid and synchronous release upon stimulation (Satir, 1977). Following the mucocyst exocytosis, the organelles are regenerated *de novo* and docked at the plasma membrane in a highly synchronous process (Haddad & Turkewitz, 1997). These organelles are dispensable for cell survival in laboratory conditions, allowing the mechanisms leading to their formation and release to be analyzed by disruption of genes essential for this pathway. Genes involved in the mucocyst pathway are tightly co-expressed, and new biogenesis-related factors have been identified by the analysis of their expression profiles (Briguglio *et al*, 2013; Kumar *et al*, 2014). To further exploit this phenomenon, we used the Coregulation Data Harvester (CDH) tool (Tsypin & Turkewitz, 2017) to automate the search of genes with expression patterns similar to those of the *Tetrahymena Nd* genes and also conserved in Apicomplexa. By this approach, we identified two novel *Tetrahymena* proteins with a role in mucocyst exocytosis. Both proteins show similarities with the cysteine repeat modular proteins (CRMPs) previously described in *Plasmodium* (Thompson *et al*, 2007; Douradinha *et al*, 2011) and two uncharacterized proteins in *Toxoplasma*, named hereafter TgCRMPa and TgCRMPb. We investigated the two uncharacterized *Toxoplasma* homologs and found that they are necessary for rhoptry exocytosis and subsequent parasite invasion. TgCRMPa and TgCRMPb are part of a complex comprising at least two additional yet uncharacterized proteins, and we demonstrated that one of them is also involved in rhoptry secretion. Unlike the exocytic Nd complex, we found that TgCRMPs are not essential for the assembly of the RSA or the anchoring of the AV to the RSA, and they only accumulate at the exocytic site just prior to the invasion and subsequently, the signal disappears at the onset of host invasion. Sequence analyses of TgCRMPs showed that they are multipass transmembrane proteins containing putative host cell-binding domains. Moreover, TgCRMPa is related to *G protein-coupled receptor* (GPCR) and exposes its host cell-binding domain toward the extracellular milieu upon egress. These features, together with their transient localization to exocytic sites, support a role for this complex in the signaling pathway that coordinates rhoptry content discharge with host contact.

Results

Tetrahymena-based strategy to search for new exocytic factors conserved in Apicomplexa

We recently demonstrated that a group of Alveolata-restricted proteins, Nd6, Nd9, NdP1, and NdP2, regulate mucocyst/trichocyst and rhoptry exocytosis in ciliates and apicomplexans, respectively (Aquilini *et al*, 2021). In addition, we found that *Toxoplasma* protein ferlin 2 (TgFer2), which has a role in rhoptry secretion (Coleman *et al*, 2018), is associated with the Nd complex. To test a conserved role of Fer2 in the two systems, we searched for the *Tetrahymena* ortholog of TgFer2 and verified its role in exocytosis. Our phylogenetic analysis of the four *Tetrahymena* ferlin genes predicted TTHERM_00886960 as the putative ortholog of TgFer2 (Fig 1A), while the other *Tetrahymena* ferlins belong to a separate subgroup. To support such an evolutionary relationship, we investigated this prediction experimentally by deleting the expressed (macronuclear) copies of *TtFer2* candidate in *Tetrahymena* cells (Fig EV1A and B). We found that the *Δ00886960* (*Δfer2*) mutant cells have a defect in mucocyst release when stimulated with the secretagogue dibucaine (Fig 1B), although the organelles appeared properly formed and docked at the plasma membrane (Fig 1C). Also arguing against any defect in biogenesis was our finding that the content protein Grl1 was proteolytically processed (Fig EV1C), an essential step in mucocyst maturation (Chilcoat *et al*, 1996). These results demonstrate a role for TTHERM_00886960 in exocytosis, and support TTHERM_00886960 as the ortholog of apicomplexans Fer2, further highlighting the conservation of exocytic mechanisms in Alveolata.

Genes involved in mucocyst exocytosis share similar patterns of expression, as shown by the transcriptional profiles of *Tetrahymena Nd6*, *Nd9*, *NdP1*, *NdP2*, and *Fer2* genes in different life stages (Fig 1D, left), while genes involved in different pathways have non-matching profiles (Fig 1D, right). We, therefore, employed a bioinformatic tool specifically developed for *Tetrahymena*, called the Coregulation Data Harvester (CDH; Tsypin & Turkewitz, 2017), to screen for other proteins with comparable patterns in the *Tetrahymena* databases (TGD, http://ciliate.org; FGD, http://tfgd.ihb.ac.cn). Since we were interested in genes with a conserved function in exocytosis in Alveolata, with a particular focus on rhoptry exocytosis in *Toxoplasma* and *Plasmodium*, we refined our analysis and set up the CDH search to look for *Tetrahymena* genes conserved specifically in *T. gondii* and *P. falciparum* (Fig 1E). We performed the CDH analysis using *Tetrahymena* Nd6, NdP1, NdP2 and Fer2 as separate queries, but excluded TtNd9 due to its very low expression level. The CDH program identified those *Tetrahymena* genes co-expressed with each selected query, and with homologs in *T. gondii* and *P. falciparum*, by BLAST and reciprocal BLAST. We then prioritized a list of candidates shared by at least three of the four queries (Fig 1E and Dataset EV1).

 © 2022 The Authors

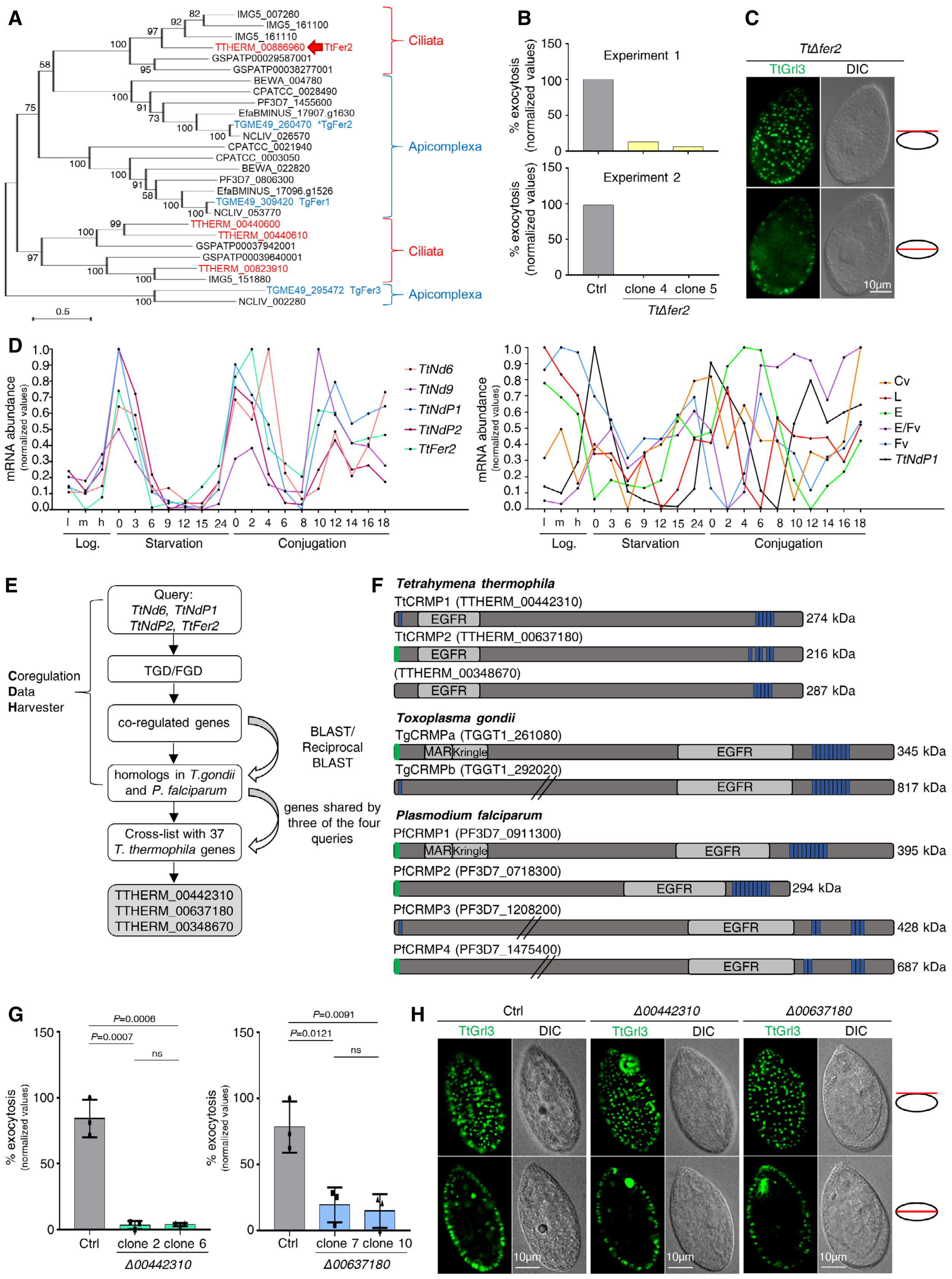
A
Ciliata
Apicomplexa
TTHERM_00886960 TtFer2
TGME49_260470 *TgFer2
TGME49_309420 TgFer1
TGME49_295472 TgFer3
B
Experiment 1
Experiment 2
% exocytosis (normalized values)
Ctrl
clone 4
clone 5
TtΔfer2
C
TtΔfer2
TtGrl3
DIC
10µm
D
mRNA abundance (normalized values)
TtNd6
TtNd9
TtNdP1
TtNdP2
TtFer2
Cv
L
E
E/Fv
Fv
TtNdP1
Log.
Starvation
Conjugation
E
Query: TtNd6, TtNdP1 TtNdP2, TtFer2
TGD/FGD
co-regulated genes
homologs in T.gondii and P. falciparum
Cross-list with 37 T. thermophila genes
TTHERM_00442310
TTHERM_00637180
TTHERM_00348670
Coregulation Data Harvester
BLAST/ Reciprocal BLAST
genes shared by three of the four queries
F
Tetrahymena thermophila
TtCRMP1 (TTHERM_00442310)
274 kDa
TtCRMP2 (TTHERM_00637180)
216 kDa
(TTHERM_00348670)
287 kDa
Toxoplasma gondii
TgCRMPa (TGGT1_261080)
345 kDa
TgCRMPb (TGGT1_292020)
817 kDa
Plasmodium falciparum
PfCRMP1 (PF3D7_0911300)
395 kDa
PfCRMP2 (PF3D7_0718300)
294 kDa
PfCRMP3 (PF3D7_1208200)
428 kDa
PfCRMP4 (PF3D7_1475400)
687 kDa
EGFR
MAR
Kringle
G
P=0.0006
P=0.0007
ns
P=0.0091
P=0.0121
clone 2 clone 6
Δ00442310
clone 7 clone 10
Δ00637180
H
Ctrl
Δ00442310
Δ00637180

Figure 1.

Figure 1. A *Tetrahymena*-based strategy identified two new non-discharge genes conserved in *Toxoplasma gondii* and *Plasmodium falciparum*.

A Phylogeny depicting the relationships between Ciliata and Apicomplexa ferlins. The maximum-likelihood phylogenetic tree was obtained with the protein sequences of ferlin genes retrieved for the ciliates *Tetrahymena thermophila* (TTHERM), *Paramecium tetraurelia* (GSPATP), and *Ichthyophthirius multifiliis* (IMG5), and for the apicomplexans *Toxoplasma gondii* (TGME49), *Plasmodium falciparum* (PF3D7), *Cryptosporidium parvum* (CPATCC), *Neospora caninum* (NCLIV), *Eimeria falciformis* (EfaB-MINUS), and *Theileria equi* (BEWA). *Tetrahymena* and *Toxoplasma* ferlins are highlighted in red and blue, respectively. The *Tetrahymena* ortholog of the rhoptry-related TgFer2 (asterisk) is indicated by the red arrow. Numbers at each node correspond to the bootstrap values. The scale bar represents the branch length.

B Quantification of the exocytic response of *Tetrahymena Δfer2* cells to dibucaine stimulation. $n = 2$ biological replicates.

C Immunofluorescence images of a *TtΔfer2* cell with paired differential interference contrast (DIC) images. Mucocysts were immunostained with mAbs 5E9 which label the granule protein Grl3, and appeared similar to wild-type (Fig 1H) in shape and docking. Single focal planes of surface (upper) and cross (lower) sections are shown for the same cell.

D Expression profiles of *Tetrahymena Nd* genes involved in mucocysts exocytosis (left graph) compared to those of genes functioning in different pathways (right graph: Cv, contractile vacuole, TTHERM_00532700; L, lysosomes, TTHERM_00716100; E, endosomes, TTHERM_00384890; E/Fv, endosomes/food vacuoles, TTHERM_00691590; Fv, food vacuoles, TTHERM_00393150; Sparvoli *et al*, 2020). The plot values were downloaded from http://tfgd.ihb.ac.cn and normalized to that of the gene's maximum expression level. The data were collected from growing (low, medium, and high culture density) and starved (S0–S24) cultures, and different time points during conjugation (C0–C18).

E *Tetrahymena*-based bioinformatics approach for identifying new exocytic factors. TGD: *Tetrahymena* Genome Database (http://ciliate.org); FGD: *Tetrahymena* Functional Genomics Database (http://tfgd.ihb.ac.cn).

F Protein domains in *T. thermophila*, *T. gondii*, and *P. falciparum* CRMPs. Epidermal growth factor receptor (EGFR), microneme adhesive repeat (MAR), and Kringle domains are shown in gray. Green: predicted signal peptide; blue: transmembrane domains; slanted lines: truncation of the full-length protein sequence.

G Quantification of the exocytic response of *Tetrahymena Δ00442310* and *Δ00637180* mutants to dibucaine stimulation. Mean ± SD ($n = 3$ biological replicates, each with two technical replicates). *P*-values were measured by two-tailed *t*-test.

H Immunofluorescence images of *Tetrahymena* cells. Mucocysts in wild-type (Ctrl) and *Δ00442310* and *Δ00637180* cells were immunostained with mAbs 5E9. The mucocyst pattern in the mutants was similar to wild-type. Single focal planes of surface and cross sections are shown for each cell. DIC, differential interference contrast.

Among the 37 *Tetrahymena* candidates identified, three (TTHERM_00442310, TTHERM_00637180, and TTHERM_00348670) encode proteins containing similar features including an epidermal growth factor receptor domain and multiple C-terminal transmembrane domains. These domains are shared by the putative homologs found in *T. gondii* and *P. falciparum* (Fig 1F). The *Plasmodium* homologs were previously described as members of a family of four genes named CRMPs for cysteine repeat modular proteins (Thompson *et al*, 2007; Douradinha *et al*, 2011), but the two *Toxoplasma* counterparts, which we called TgCRMPa (TGGT1_261080) and TgCRMPb (TGGT1_292020), had not been previously studied. In addition to the common features, TgCRMPa and PfCRMP1 possess a Kringle domain known to bind proteins (Patthy *et al*, 1984). Secondary structure-based predictions (Zimmermann *et al*, 2018) revealed that TgCRMPa and PfCRMP1 also possess a microneme adhesive repeat (MAR) domain at the N-terminus, which is a novel carbohydrate-binding domain found in microneme proteins of enteroparasitic coccidians and known to interact with sialic acids (Blumenschein *et al*, 2007; Friedrich *et al*, 2010). Interestingly, TgCRMPa and PfCRMP1 are also predicted to be *G protein-coupled receptor* (GPCR)-like proteins by PANTHER analysis (Mi *et al*, 2021). These similarities between TgCRMPa and PfCRMP1 are consistent with their evolutionary relatedness (Fig EV1D).

To validate the *in silico* screening, we first knocked-out the three *Tetrahymena* genes (Fig EV1A). We obtained complete knockout lines for the genes TTHERM_00442310 and TTHERM_00637180 (Fig EV1E) but not for TTHERM_00348670. *Δ00442310* and *Δ00637180* cells were impaired in mucocyst discharge (Fig 1G) but not in biogenesis, as judged by normal mucocyst staining (Fig 1H) and correct processing of the Grl1 precursor (Fig EV1F). We concluded that the affected step was exocytosis. These data showed that TTHERM_00442310 and TTHERM_00637180 are two novel non-discharge proteins, and prompted us to study the function of their apicomplexan CRMP homologs.

TgCRMPa and TgCRMPb are essential for rhoptry secretion and host cell invasion

PfCRMP1 and PfCRMP2 are not essential for the asexual stage of *P. berghei*, but they appear to control sporozoite invasion of the mosquito salivary glands (Thompson *et al*, 2007; Douradinha *et al*, 2011). We tested the function of CRMP proteins in the apicomplexan model *T. gondii*. TgCRMPa and TgCRMPb are predicted to be fitness-conferring genes in tachyzoites (Sidik *et al*, 2016); thus, we generated inducible knockdown lines (iKD). We introduced a triple HA tag at the C-terminus of TgCRMPa and TgCRMPb (Fig EV2A and B) and then replaced the endogenous promoter of each gene with the anhydrotetracycline (ATc)-regulatable TetOSag4 promoter (Fig EV2C and D) to switch off gene expression by using ATc (Meissner *et al*, 2002). Two bands were detected by western blot for both TgCRMPa-HA_3 and TgCRMPb-HA_3 and appeared less abundant in the ATc-untreated (0 h) iKD lines compared to the solely HA_3-tagged lines (Fig 2A), indicating that the promoter switch reduced transcription of both *TgCRMPs* genes. The two bands might reflect proteolytic processing, and both disappeared in the iKD lines upon ATc treatment (24–48 h; Fig 2A). Expression and efficient depletion of the tagged proteins were also confirmed by immunofluorescence microscopy (Figs 2B and EV2E). We observed diffuse punctate staining of TgCRMPa-HA_3 and TgCRMPb-HA_3 dispersed in the parasite cytosol that disappears upon ATc incubation. Occasionally, the staining appeared more concentrated at the apex of the tachyzoite, similar to micronemes visualized using antibodies to AMA1 (Fig 2B). The apical concentration of TgCRMPs was more evident in the untreated iKD lines (−ATc; Figs 2B and C, and EV2E), likely due to lower levels of the proteins, as shown in Fig 2A. However, they did not extensively co-localize with the microneme proteins AMA1, MIC2, GAMA, and PLP1 by confocal microscopy, as shown for TgCRMPb-HA_3_iKD (Figs 2D and EV2F and G).

We tested the overall ability of TgCRMPs_iKD lines to proliferate and lyse host cells, and found that treatment with ATc (+ATc)

 © 2022 The Authors

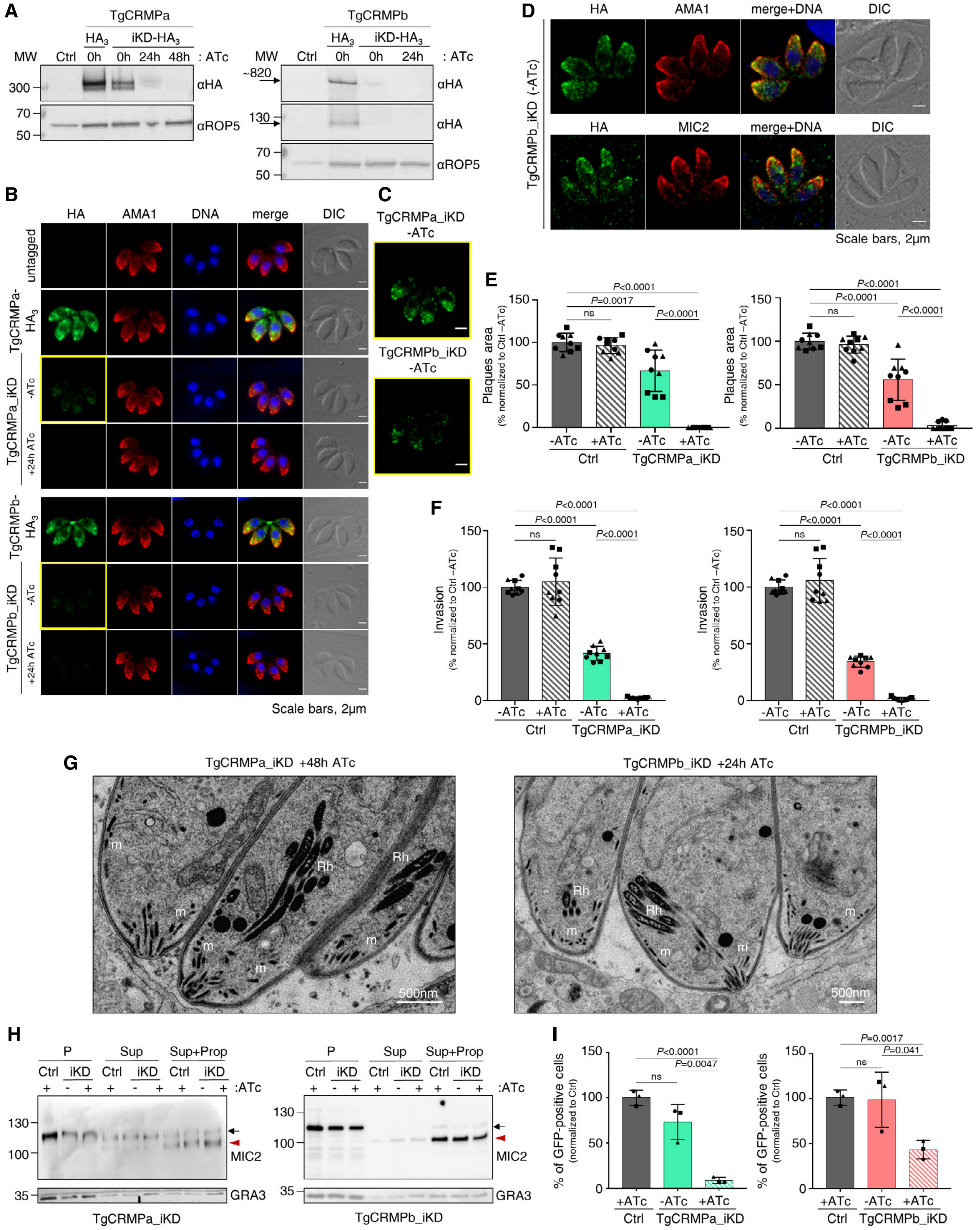
A
TgCRMPa
HA3 iKD-HA3
MW Ctrl 0h 0h 24h 48h : ATc
300
70
50
αHA
αROP5
TgCRMPb
MW Ctrl 0h 0h 24h : ATc
~820
130
70
50
αHA
αHA
αROP5
B
HA AMA1 DNA merge DIC
untagged
TgCRMPa-HA3
TgCRMPa_iKD -ATc +24h ATc
TgCRMPb-HA3
TgCRMPb_iKD -ATc +24h ATc
Scale bars, 2μm
C
TgCRMPa_iKD -ATc
TgCRMPb_iKD -ATc
D
HA AMA1 merge+DNA DIC
HA MIC2 merge+DNA DIC
TgCRMPb_iKD (-ATc)
Scale bars, 2μm
E
Plaques area (% normalized to Ctrl -ATc)
P<0.0001
P=0.0017
ns
P<0.0001
-ATc +ATc -ATc +ATc
Ctrl TgCRMPa_iKD
P<0.0001
P<0.0001
ns
P<0.0001
Ctrl TgCRMPb_iKD
F
Invasion (% normalized to Ctrl -ATc)
P<0.0001
P<0.0001
ns
P<0.0001
-ATc +ATc -ATc +ATc
Ctrl TgCRMPa_iKD
Ctrl TgCRMPb_iKD
G
TgCRMPa_iKD +48h ATc
TgCRMPb_iKD +24h ATc
m
Rh
500nm
H
P Sup Sup+Prop
Ctrl iKD Ctrl iKD Ctrl iKD
+ - + + - + + - + :ATc
130
100
35
MIC2
GRA3
TgCRMPa_iKD
TgCRMPb_iKD
I
% of GFP-positive cells (normalized to Ctrl)
P<0.0001
ns
P=0.0047
+ATc -ATc +ATc
Ctrl TgCRMPa_iKD
P=0.0017
P=0.041
ns
Ctrl TgCRMPb_iKD

Figure 2.

Figure 2. TgCRMPa and TgCRMPb are essential for rhoptry secretion and host cell invasion in *Toxoplasma*.

A Immunoblot with anti-HA Abs of lysates from parental (Ctrl) and tagged lines (TgCRMPa-HA_3 and TgCRMPb-HA_3) together with inducible-knockdown lines (TgCRMPa-HA_3_iKD and TgCRMPb-HA_3_iKD) treated with ATc for 0, 24, or 48 h. TgROP5 was used as a loading control. Two close bands around 300 kDa were detected for TgCRMPa. A ~ 820 kDa protein, corresponding to the predicted size for TgCRMPb, was observed together with a ~ 130 kDa band.

B Immunofluorescence microscopy of intracellular parasites (untagged, TgCRMPa-HA_3, TgCRMPb-HA_3, and TgCRMPs-depleted (iKD) lines). Parasites were labeled with anti-HA and anti-AMA1 Abs to visualize CRMPs-HA_3 and micronemes, respectively. The nuclei (DNA) are stained with Hoechst. DIC: differential interference contrast. TgCRMPs-HA_3 shows a heterogeneous distribution within the parasite cytosol, occasionally showing a microneme-like apical gradient (yellow boxes), highlighted in (C). Shown are single focal planes.

C Images in yellow boxes are shown in (B) with increased contrast and brightness.

D Confocal immunofluorescence images of TgCRMPb-depleted (iKD) intracellular tachyzoites. Parasites were stained with anti-HA and with anti-AMA1 and anti-MIC2 Abs to visualize TgCRMPb and micronemes, respectively. The nuclei (DNA) are stained with Hoechst. Shown are single focal planes.

E Quantification of plaques areas for control and TgCRMPa_iKD and TgCRMPb_iKD in the absence of ATc, and upon 24 and 48 h ATc treatment for TgCRMPb and TgCRMPa, respectively. Values are reported as mean ± SD (n = 3 biological replicates, each with three technical replicates). The biological replicates are represented by different symbols.

F Invasion of TgCRMPa- and TgCRMPb-depleted tachyzoites upon 48 and 24 h treatment with ATc, respectively. Data are reported as in (E; n = 3 biological replicates, each with three technical replicates). The biological replicates are represented by different symbols.

G Electron micrographs of TgCRMPa_iKD and TgCRMPb_iKD intravacuolar parasites treated with ATc for 48 and 24 h, respectively. Micronemes (m) and rhoptries (Rh) appeared properly localized and shaped in both mutants.

H Quantification of microneme secretion in TgCRMPa- and TgCRMPb-depleted tachyzoites was measured by detecting the processed form (arrowhead) of TgMIC2 (arrow) in the media. Control and TgCRMPa_iKD and TgCRMPb_iKD parasites, ATc-treated (+) and untreated (−), were stimulated with propranolol to release microneme contents. Blots were probed with anti-MIC2 (secretion of micronemes) and anti-GRA3 (constitutive secretion of dense granules). P, Parasites pellet. Sup, Supernatant from untreated parasites. Sup + Prop, Supernatant from parasites treated with propranolol. The results are representative of two independent experiments.

I Quantification of rhoptry secretion in TgCRMPa_iKD and TgCRMPb_iKD parasites upon 48 and 24 h ATc treatment, respectively, using the SeCreEt system (Koshy *et al*, 2010). Successful secretion of rhoptry proteins into the host causes a switch from red to green fluorescence in a reporter host cell line. CRMPs-depleted parasites were unable to efficiently deliver rhoptry content into the host cytosol. Data are represented as mean ± SD (n = 3 biological replicates). The biological replicates are represented by different symbols.

Data information: P-values in (E, F, and I), were measured by two-tailed t-test.
Source data are available online for this figure.

resulted in the loss of plaque formation; TgCRMPb_iKD parasites exhibited significant defects in plaque formation even in the absence of ATc (−ATc; Fig 2E). Importantly, parasites could efficiently replicate, egress from the PV, and attach to host cells (Fig EV2H–J) but were severely impaired in host cell invasion (Fig 2F). Invasion depends on the sequential secretion of microneme and rhoptry proteins. Since the morphology and positioning of both organelles appeared unaltered by ATc treatment (Figs 2B and G, and EV2E), we tested whether their discharge was disrupted. While microneme secretion occurred normally in TgCRMPs-depleted parasites (Fig 2H), the discharge of rhoptry contents into the host cell was greatly impaired (Fig 2I). We conclude that CRMP proteins serve a crucial function, conserved between Ciliata and Apicomplexa, in the regulated discharge of secretory organelles. We named *Tetrahymena* TTHERM_00442310 and TTHERM_00637180, TtCRMP1 and TtCRMP2, respectively.

TgCRMPa and TgCRMPb form a complex with two additional membrane proteins

TgCRMPa and TgCRMPb have similar organization and function, suggesting that they might collaborate in regulating rhoptry secretion. To test this, we isolated each TgCRMP-HA_3 and its associated proteins by affinity capture (Fig EV3A) and analyzed the associated proteins by liquid chromatography–tandem mass spectrometry (Datasets EV2 and EV3). Indeed, TgCRMPa and TgCRMPb were associated with each other (Fig 3A), a result also confirmed by co-immunoprecipitation experiments with parasites co-expressing TgCRMPa-$FLAG_3$ and TgCRMPb-HA_3 (Figs 3B and EV3B–D). Moreover, TgCRMPs robustly associate with two additional uncharacterized membrane proteins (Fig 3A and C), Tg247195 and Tg277910. Tg277910 and Tg247195 possess one and three thrombospondin type 1 (TSP-1) domains, respectively (Fig 3C), known to participate in cell adhesion (Adams & Tucker, 2000). In addition, Tg247195 possesses an H-type lectin domain (Pietrzyk-Brzezinska & Bujacz, 2020) and, interestingly, has a role in invasion (preprint: Singer *et al*, 2022; Possenti *et al*, 2022) and rhoptry secretion (Possenti *et al*, 2022). To determine the function of Tg277910, we generated an inducible knockdown HA_3-tagged line (Tg277910_iKD; Fig EV3E–G). A single Tg277910-HA_3 band was detected by western blot in the absence of ATc, and the protein was undetectable after ATc treatment in both western blot (Fig EV3H) and IFA (Figs 3D and EV3I). We observed a consistent reduction in the area of lytic plaques in ATc-treated tachyzoites (Fig EV3J) that was not related to the disruption of parasite replication, stimulated egress, or attachment (Fig EV3K–M), but a consequence of the inability of the parasites to invade the host cell (Fig 3E). This defect was associated with loss of rhoptries discharge (Fig 3F), but not that of micronemes (Fig 3G). We note that again the morphology and localization of these two secretory organelles were not affected by protein depletion (Figs 3D and EV3I).

We did not find any of the rhoptry exocytic factors described previously (TgNd6, TgNd9, TgNdP1, TgNdP2, and TgFer2) among the proteins co-isolated with TgCRMPs, suggesting that CRMPs are part of a distinct complex regulating rhoptry secretion, a result also supported by the mass spectrometry analysis of Nd9 and NdP1 pull-downs (Aquilini *et al*, 2021).

Toxoplasma and *Tetrahymena* CRMP proteins are not required for rosette formation, RSA assembly, or AV positioning in *T. gondii*

Our findings on *Toxoplasma* and *Tetrahymena* CRMPs strongly suggest that they have a role in exocytosis, the last step of the secretory pathway, which depends on the proper assembly of the rosette at the plasma membrane (Plattner *et al*, 1973; Aquilini *et al*, 2021).

 © 2022 The Authors

Since CRMPs are predicted to be transmembrane proteins (Fig 1F), we considered that they might be rosette components. To test this hypothesis, we performed thin-section and freeze-fracture electron microscopy (EM) analyses of CRMP mutants. *Tetrahymena* mutant *Δcrmp1* accumulated well-formed rosettes at the plasma membrane as shown by freeze-fracture EM of the cell surface (Fig 4A and B), arrayed in the known pattern of mucocyst docking sites (Fig 4C). In *Toxoplasma*, no apparent defects were observed in the positioning of the AV in CRMPs_iKD strains after ATc treatment (Fig 4D) or was there an apparent defect in the assembly of the rosette, as shown for TgCRMPa-depleted tachyzoites (Fig 4E). To inspect possible minor defects affecting the RSA, we performed cryo-electron tomography (cryo-ET) on frozen-hydrated TgCRMPb-depleted cells. The subtomogram average of the RSA showed an eightfold symmetry of defined densities holding the AV as seen previously in the wild-type (Mageswaran *et al*, 2021; Fig 4F). We did not observe profound rearrangements of the RSA densities and their distance to the AV in the TgCRMPb-depleted parasites compared to wild-type (Fig 4G and H), in stark contrast to what we previously showed after TgNd9 depletion (Mageswaran *et al*, 2021). We only observed a minor alteration in the AV shape and anchoring angle (Fig 4G and I–K). In conclusion, since freeze-fracture EM and

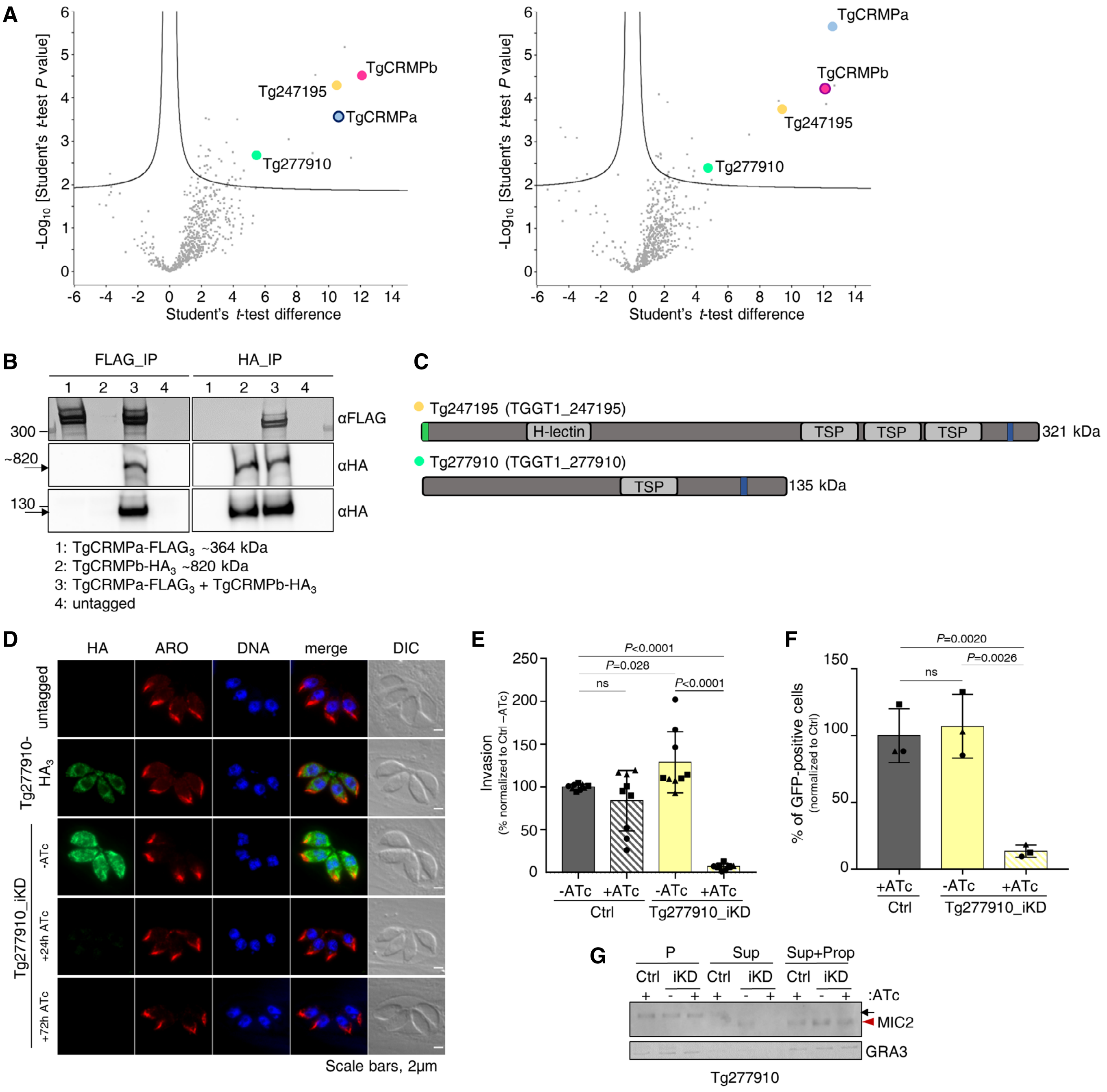

Figure 3.

Figure 3. TgCRMPa and TgCRMPb are in complex with two additional membrane proteins, one of which is required for rhoptry exocytosis.

A Mass spectrometric identification of proteins co-isolated with HA_3-tagged TgCRMPa (left plot) and TgCRMPb (right plot). The volcano plots shown here were generated by plotting the $\log_{10}$ *t*-test *P*-value versus the *t*-test difference. The colored dots mark members of the CRMP complex. Baits are indicated by darker outlines.

B Co-immunoprecipitation of TgCRMPa and TgCRMPb. Lysates from parasites co-expressing TgCRMPa-$FLAG_3$ and TgCRMPb-HA_3 were split and incubated with either anti-FLAG (left panels) or anti-HA beads (right panels). Eluates were subjected to SDS–PAGE and immunoblotted with anti-HA and anti-FLAG Abs. Untagged, TgCRMPa-$FLAG_3$ and TgCRMPb-HA_3 parasites were used as controls. TgCRMPs robustly associate with each other.

C Protein domains of Tg247195 and Tg277910, co-purified with TgCRMPs. TSP: thrombospondin domain; H-lectin: lectin-binding domain. Green: predicted signal peptides; blue: transmembrane domains.

D Immunofluorescence images of untagged and Tg277910-HA_3 and Tg277910_iKD intracellular tachyzoites. Parasites were stained with anti-HA and anti-ARM (ARO) Abs to label Tg277910 and rhoptries, respectively. The nuclei (DNA) are stained with Hoechst. Tg277910 localization is similar to that of TgCRMPs, with an increased fluorescence in the iKD line (−ATc). Tg277910 signal almost completely disappeared upon 72 h ATc treatment. Single focal planes are shown.

E Invasion of parental and Tg277910_iKD lines in the absence of ATc, and upon 72 h ATc treatment. Values are reported as mean ± SD (n = 3 biological replicates, each with three technical replicates). The biological replicates are represented by different symbols.

F Quantification of rhoptry secretion in Tg277910_iKD by SeCreEt system as described in Fig 2I. Tg277910-depleted parasites failed to deliver rhoptry content into the host cytosol. Values are reported as mean ± SD (n = 3 biological replicates). The biological replicates are represented by different symbols.

G Quantification of microneme secretion in control and Tg277910-depleted tachyzoites was measured as in Fig 2H. Blots were probed with anti-MIC2 (secretion of micronemes) and anti-GRA3 (constitutive secretion of dense granules). P, Parasites pellet. Sup, Supernatant from untreated parasites. Sup + Prop, Supernatant from parasites treated with propranolol. The results are representative of two independent experiments.

Data information: *P*-values in (E and F) were measured by a two-tailed *t*-test.
Source data are available online for this figure.

cryo-ET demonstrate that CRMPs are not essential for building the rhoptry secretion machinery, CRMPs have a function different from that of the previously described Nd complex.

TgCRMPa and TgCRMPb accumulate at the tip of the extruded conoid in extracellular tachyzoites

Since the CRMPs labeling was reminiscent of MICs, which are typically released on the surface of parasite upon egress, we analyzed the location of CRMPs in extracellular parasites. TgCRMPa-HA_3 and TgCRMPb-HA_3 were found to consistently accumulate at the tip of the extruded conoid in freshly egressed parasites kept in contact with host cells (Fig 5A, left panels) and in those treated with the calcium ionophore A23187 (Fig 5A, right panels and 5B), which artificially induces conoid extrusion (Mondragon & Frixione, 1996) and microneme secretion (Carruthers & Sibley, 1999). This staining appears as a tiny dot at the apex of the parasite and thus contrasts with the wide redistribution of MICs proteins at the surface of the parasite (Carruthers & Sibley, 1999). This accumulation did not occur upon TgCRMPa depletion (Fig EV4A), indicating that it was not a staining artifact.

To test whether the apical localization of TgCRMPa and TgCRMPb were interdependent, we generated an inducible knockdown (iKD) for TgCRMPa, in which TgCRMPa was tagged with a triple FLAG tag and

Figure 4. TgCRMPa and TgCRMPb are dispensable for apical vesicle positioning and, similarly to TtCRMP1, show a well-assembled rosette.

A Freeze-fracture electron micrograph of *Tetrahymena Δcrmp1* cell surface showing a representative rosette (red box) at the plasma membrane. On the right, the magnified rosette with the eight intramembranous particles (IMPs, arrowheads) surrounding the central one.

B Freeze-fracture electron micrographs of larger cell surfaces for *TtΔcrmp1* and wild-type (control) in which multiple rosettes (red circles), corresponding to multiple mucocyst docking sites, are correctly aligned along 2° meridians in both lines.

C Cartoon of a *Tetrahymena* cell (surface section). Mucocysts primarily occupy sites along 2° meridians which mark the membrane spaced between two consecutive 1° meridians, defined in turn by longitudinal rows of basal bodies (BB). Mucocysts are also found at a lower frequency between BBs. 1° and 2° meridians regularly span the length of the cell, from the anterior oral apparatus (OA) to the cell posterior.

D Electron micrographs of TgCRMPa_iKD and TgCRMPb_iKD intracellular tachyzoites treated with ATc for 48 and 24 h, respectively. A well-formed apical vesicle (AV) appears correctly positioned at the parasite apex in both mutants (magnified red box, arrow). Rh rhoptry; m microneme; co conoid; PCR pre-conoidal rings, indicated by arrows.

E Freeze-fracture electron micrographs of the apex of a TgCRMPa_iKD extracellular tachyzoite treated with ATc for 48 h. A well-assembled rosette (red box) was observed at the center of the parasite apex, and magnified on the right with the eight IMPs indicated by arrowheads.

F A tomogram slice showing the apical vesicle (AV) anchoring on the plasma membrane in TgCRMPb-iKD line; anchoring is mediated by a rhoptry secretory apparatus (RSA) that is morphologically undistinguishable from the wild-type (WT). Left: side view presented by a central slice through the AV and the RSA. Right: top view presented by a slice through the subtomogram average, revealing the RSA densities that anchor the AV.

G AV dimensions and anchoring parameters in WT and TgCRMPb-iKD. Left: a schematic depicting the parameters. Right: a table summarizing their measurements.

H AV anchoring distance (the shortest distance measured from the parasite apex to the AV membrane).

I AV dimensions (AV_{maj} and AV_{min}).

J AV eccentricity (Ecc) calculated using the AV dimensions (AV_{maj} and AV_{min}) in panel (I).

K AV orientation parameter (Ψ) that measures the AV anchoring angle.

Data information: Panels (H), (J), and (K) show a combination of boxplot and swarmplot for each dataset. For the boxplot, the lower and upper boundaries of the box represent the first and third quartiles (Q1 and Q3), whiskers extend to 1.5 times the interquartile range (Q3–Q1) below and above Q1 and Q3, and points outside (diamonds) are regarded as outliers. The horizontal divider within the box represents the median with the value noted next to it. For the swarmplot, each data point represents a measurement from a tomogram. Panel (I) shows a jointplot, which is a combination of a bivariate scatterplot and two marginal univariate kernel density estimate plots (a.k.a. probability density plots), one each for AV_{maj} and AV_{min}. Mann–Whitney *U* tests were used to calculate the *P*-values. A total of 22 and 37 tomograms for each strain were used for measurements in (H–J and K), respectively.

© 2022 The Authors

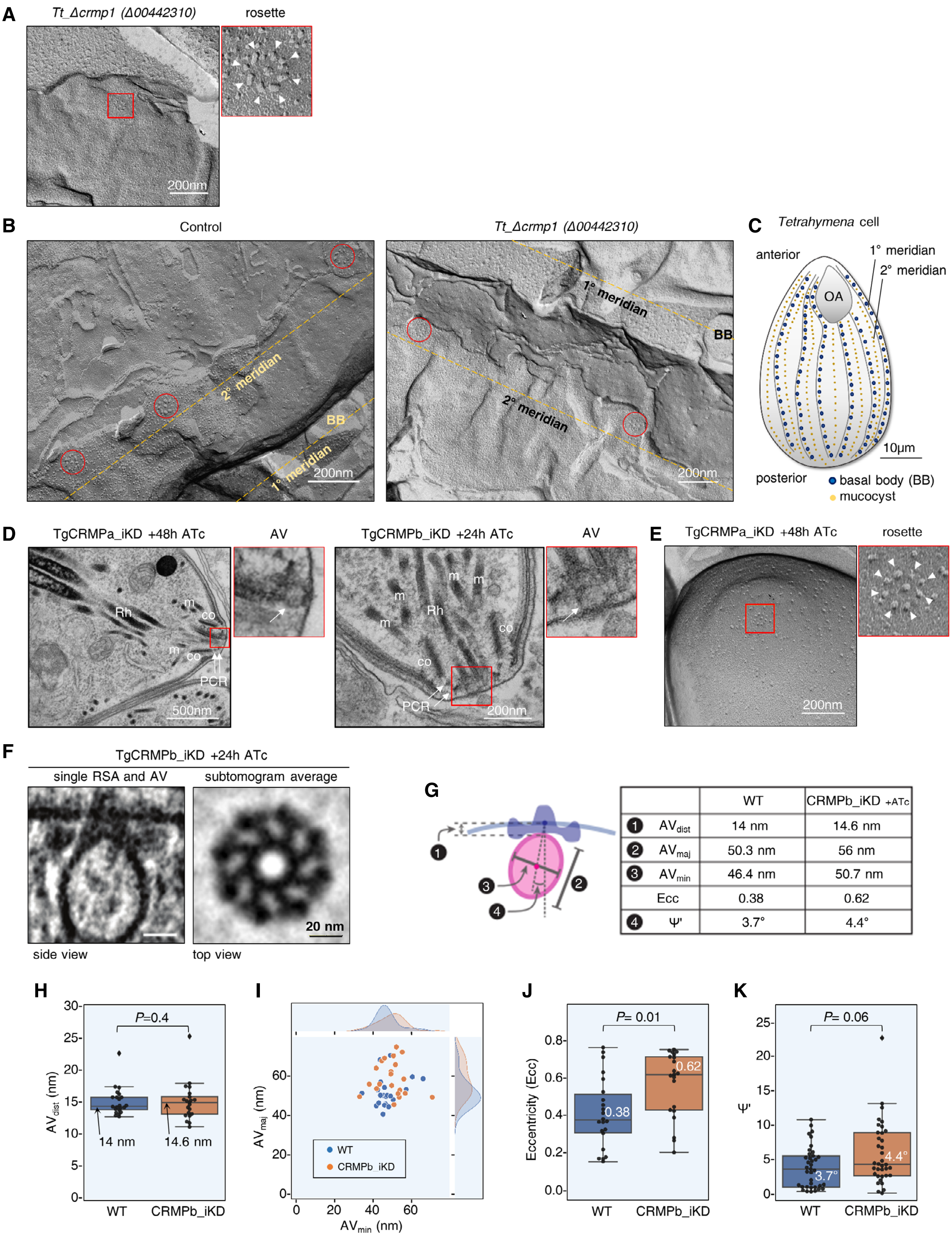
A
Tt_Δcrmp1 (Δ00442310)
rosette
200nm
B
Control
2° meridian
BB
1° meridian
200nm
Tt_Δcrmp1 (Δ00442310)
1° meridian
BB
2° meridian
200nm
C
Tetrahymena cell
anterior
1° meridian
2° meridian
OA
10µm
posterior
basal body (BB)
mucocyst
D
TgCRMPa_iKD +48h ATc
AV
Rh
m
co
PCR
500nm
TgCRMPb_iKD +24h ATc
AV
m
Rh
co
PCR
200nm
E
TgCRMPa_iKD +48h ATc
rosette
200nm
F
TgCRMPb_iKD +24h ATc
single RSA and AV
subtomogram average
20 nm
side view
top view
G
1
2
3
4
	WT	CRMPb_iKD +ATc
1 AVdist	14 nm	14.6 nm
2 AVmaj	50.3 nm	56 nm
3 AVmin	46.4 nm	50.7 nm
Ecc	0.38	0.62
4 Ψ'	3.7°	4.4°
H
P=0.4
AVdist (nm)
14 nm
14.6 nm
WT
CRMPb_iKD
I
AVmaj (nm)
AVmin (nm)
WT
CRMPb_iKD
J
P= 0.01
Eccentricity (Ecc)
0.38
0.62
WT
CRMPb_iKD
K
P= 0.06
Ψ'
3.7°
4.4°
WT
CRMPb_iKD

Figure 4.

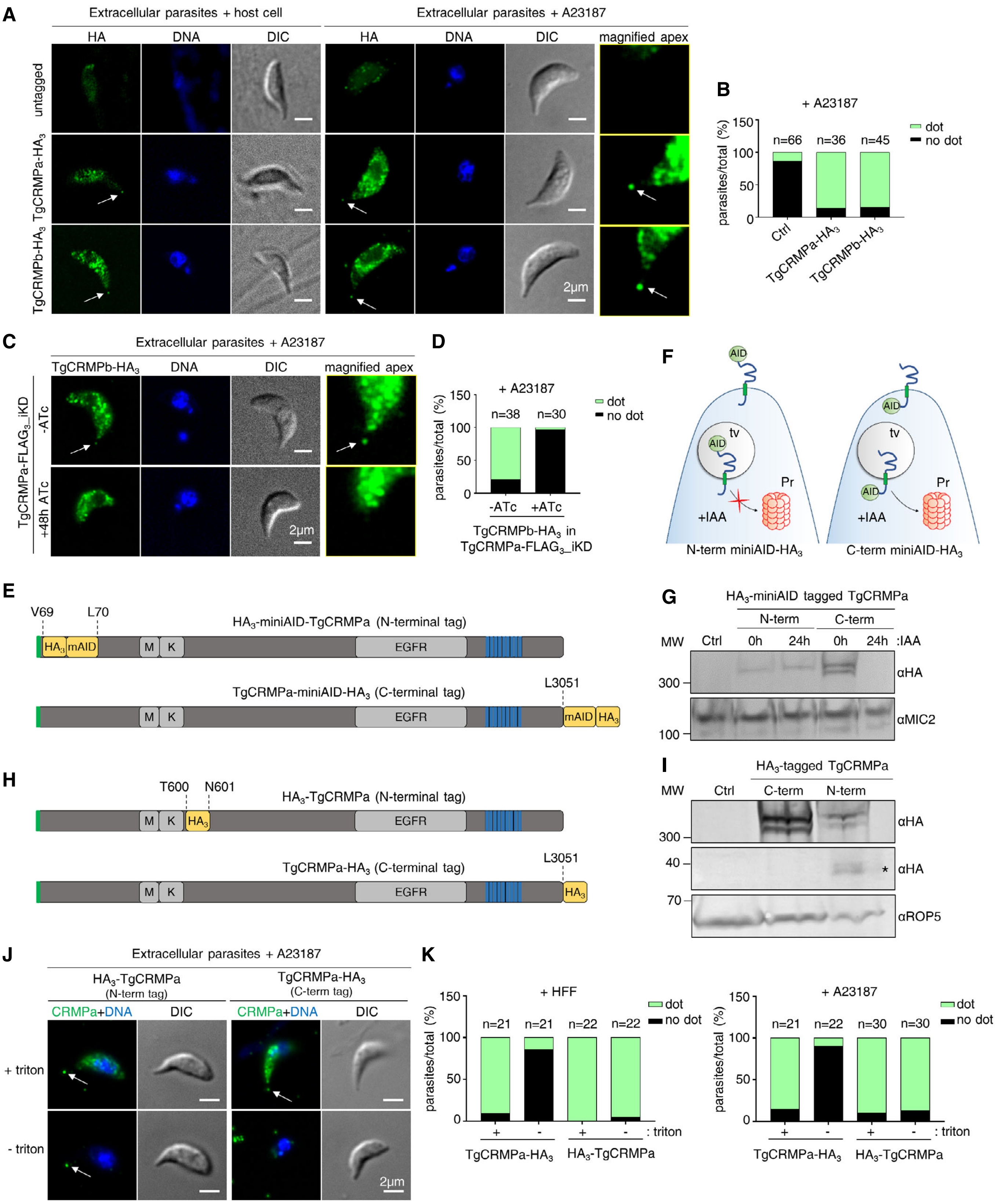

Figure 5.

© 2022 The Authors

Figure 5. TgCRMPa and TgCRMPb accumulate at the apical tip of extracellular tachyzoites, with TgCRMPa N-terminal end oriented toward the outside space.

A Immunofluorescence images of extracellular tachyzoites of untagged and TgCRMPa-HA_3 and TgCRMPb-HA_3 parasites, incubated either with host cell monolayers for 2 min, or with ionophore A23187, to induce natural or artificial conoid extrusion, respectively. Parasites were immunostained with anti-HA Abs; DNA was labeled by Hoechst. CRMPa and CRMPb consistently accumulate at the tip of extruded conoids (arrows). The apexes of A23187-treated parasites were magnified on the right and increased in brightness and contrast to highlight the apical dots. DIC: differential interference contrast. Single focal planes are shown.

B Quantification of the dot pattern upon A23187 treatment shown in (A). Values are expressed as the percentage of parasites showing (dot) or lacking (no dot) the apical accumulation of TgCRMPa and TgCRMPb; *n*, number of parasites analyzed per line.

C Immunofluorescence images of extracellular TgCRMPb-HA_3 tachyzoites in the presence (-ATc) or absence (+48 h ATc) of TgCRMPa-$FLAG_3$ (iKD line). Parasites were stained with anti-HA Abs to visualize CRMPb. TgCRMPb localization at the tip of the extruded conoid (arrow) disappears upon TgCRMPa depletion, but it is still detected in the cytoplasm (lower panel). DNA is labeled by Hoechst. Single focal planes are shown. DIC, differential interference contrast.

D Quantification of the dot pattern shown in (C). The values are reported as in (B).

E Schematic representation of the N- (top) and C-terminal (bottom) tagging of TgCRMPa with the triple HA and the miniAID. The amino acid residues where the insertion of the tag occurred are indicated with dotted lines. M: MAR domain; K: Kringle domain; EGFR: epidermal growth factor receptor. Green: predicted signal peptide; blue: transmembrane domains.

F Cartoon depicting the targeting of a membrane protein to the proteasome (Pr) by the AID-degron system when the AID-fused C-terminus is exposed to the cytosol. IAA: 3-indoleacetic acid or auxin; AID: auxin-inducible degron; tv: transport vesicle.

G Whole-cell lysates from TIR1-expressing parental line (Ctrl), HA_3-miniAID-TgCRMPa_iKD (N-term), and TgCRMPa-miniAID-HA_3_iKD (C-term) lines were immunoblotted with anti-HA Abs to visualize tagged CRMPa in IAA-treated and untreated samples. CRMPa was undetectable upon 24 h incubation with IAA when C-terminally, but not N-terminally, tagged with the miniAID-HA_3, suggesting that the C-terminus is the one exposed toward the cytosol. TgMIC2 was used as loading control and detected with anti-MIC2 Abs. MW, molecular weight standards.

H Schematic representation of the N- (top) and C-terminal (bottom) tagging of TgCRMPa with the triple HA. The amino acid residues where the insertion of the tag occurred are indicated with dotted lines. The domains are indicated as in (E).

I Whole-cell lysates from C-terminally and N-terminally HA_3-tagged TgCRMPa lines were immunoblotted as in (G). In the "N-term" lane, in addition to the full-length and processed form of CRMPa, a smaller band (asterisk) is also visible by anti-HA Abs staining. Untagged parasites (Ctrl) were treated in parallel. TgROP5 was used as a loading control and detected with anti-ROP5 Abs. MW, molecular weight standards.

J Immunofluorescence images of extracellular A23187-treated parasites expressing either N- or C-terminally HA_3-tagged TgCRMPa, and immunostained as in (A). TgCRMPa accumulates at the tip of extruded conoids (arrows) in triton-permeabilized (+) or non-permeabilized (−) parasites. DIC, differential interference contrast. Single focal planes are shown.

K Quantification of the dot pattern upon natural (+Human Foreskin Fibroblasts) or artificial (+A23187) conoid extrusion in parasites expressing either N- or C-terminally HA_3-tagged TgCRMPa. Values are reported as in (B).

Source data are available online for this figure.

TgCRMPb with a triple HA tag (Fig EV4B and C). Upon depletion of TgCRMPa-$FLAG_3$ by ATc treatment (Fig EV4D), TgCRMPb-HA_3 was readily detected in the cytosol but its apical accumulation disappeared (Fig 5C and D), suggesting that the localization of TgCRMPb-HA_3 at the tip of extracellular parasites is dependent on the interaction with TgCRMPa.

Altogether, these results suggest that TgCRMPa and TgCRMPb are potentially associated together with the site of rhoptry exocytosis.

TgCRMPa is a transmembrane protein with a cytosolic C-terminus and the N-terminal domains facing the extracellular space

Modeling the topology of CRMPs by TMHMM (http://www.cbs.dtu.dk/services/TMHMM/) and TOPCONS (http://topcons.cbr.su.se) predicted the C-terminal ends extending toward the parasite cytosol and the adhesion domains facing the extracellular space. To experimentally validate this prediction, we tagged TgCRMPa with the auxin-inducible degron (mAID) system either at the C-terminus (TgCRMPa-miniAID-HA_3; Fig EV4E and F) or N-terminus (HA_3-miniAID-TgCRMPa) before the MAR/Kringle domain (Fig EV4G and H), as depicted in Fig 5E. The mAID tag targets the fusion protein to the proteasome upon the addition of 3-indoleacetic acid (IAA or auxin) when it is topologically oriented toward the cytosol (Fig 5F; Nishimura *et al*, 2009). After adding IAA to the medium, we observed depletion of C-terminally, but not N-terminally tagged TgCRMPa, by western blot and immunofluorescence (Figs 5G and EV4I), indicating that the C-terminus of TgCRMPa is indeed found in the cytosol, while the N-terminus likely faces the lumen of the putative transport vesicle. Confirming our previous findings, TgCRMPa-miniAID-HA_3 IAA-dependent degradation blocked the mutant's ability to form plaques in host cell monolayers (Fig EV4J).

The subcellular localization of N- and C-terminally miniAID-HA_3-tagged TgCRMPa proteins was similar to that of TgCRMPa-HA_3, however, the former appears less abundant in intracellular parasites (Fig EV4I), in agreement with the western blot result (Fig 5G), and undetectable at the apical dot in extracellular parasites (Fig EV4K and L). Moreover, two bands were detected by western blot for TgCRMPa-miniAID-HA_3 (similar to TgCRMPa-HA_3, Fig 2A), while only the upper band of HA_3-miniAID-TgCRMPa was visible (Fig 5G). This phenotype is consistent with proteolytic cleavage of CRMPa at the N-terminal end after the HA_3-miniAID tag, which likely prevents the visualization of the mature form of TgCRMPa by western blot and IFA, either in IAA-treated or untreated parasites. We could not detect the cleaved form by western blot but the ~ 50 kDa shift between the two higher forms of CRMPa seen with the C-terminal tags, suggests that the proteolytic cleavage occurs after the tag but before the MAR/Kringle domain (Fig 5E). Another possibility is that the insertion of the tag perturbed the protein processing, but this scenario is unlikely since there is no evident accumulation of the full-length protein compared to the C-terminally tagged one (Fig 5G).

To ultimately determine whether the N-terminal domain of TgCRMPa is exposed toward the extracellular milieu, we generated another cell line (hereafter called HA_3-TgCRMPa) where a triple HA tag was added after the MAR/Kringle domain between residues Thr600 and Asn601 (Figs 5H and EV4M and N). The HA_3-TgCRMPa protein showed the same profile as the C-terminal tagged version by

© 2022 The Authors

western blot, with both pro- and mature forms labeled with anti-HA antibodies (Fig 5I). A fragment of ~ 35 kDa can be also seen in the same blot, suggesting that there is another cleavage site at the N-terminus of TgCRMPa downstream of the triple HA tag, which generates a cleaved form containing the MAR/Kringle domain (Fig 5H). The HA_3-TgCRMPa protein appears to have the same cytosolic distribution as the other tagged versions of CRMPa in intracellular parasites (Fig EV4O). We then investigated the apical localization of this new fusion protein in extracellular parasites by immunofluorescence in both permeabilizing and non-permeabilizing conditions (Fig 5J and K). Strikingly, while the C-terminal tagged version is only visible at the apex of the parasite upon membrane permeabilization, an apical dot is consistently observed in the presence or absence of detergent for HA_3-TgCRMPa parasites (Fig 5J and K), demonstrating that the N-terminal domain of the protein is exposed extracellularly.

TgCRMPa and TgCRMPb accumulate at the site of exocytosis with TgNd6 but behave differently during an invasion

The apical accumulation of TgCRMPa and TgCRMPb in extracellular parasites was reminiscent of that of TgNd6, a protein related to the rhoptry secretory machinery, in intracellular parasites (Aquilini *et al*, 2021). TgNd6 distribution in extracellular parasites was not investigated in our previous work. To assess if CRMPs and Nd6 co-localize at the site of rhoptry exocytosis in extracellular parasites, we generated *T. gondii* strains co-expressing TgCRMPa-HA_3 or TgCRMPb-HA_3 with TgNd6-TY_2 (Fig EV5A–C). TgCRMPs and TgNd6 appeared to occupy distinct compartments in intracellular parasites, with only Nd6 puncta at the apical ends of tachyzoites (Fig 6A, TgCRMPa; Fig EV5D, TgCRMPb), as previously shown (Aquilini *et al*, 2021). Remarkably, we found TgNd6 overlapping with TgCRMPs at the tip of the extruded conoid in extracellular parasites (Figs 6A and EV5D, lower panels), a result confirmed using ultrastructure expansion microscopy (U-ExM; Fig 6B). Upon parasite expansion, we could measure a ~ 40% overlap between C-terminally tagged TgCRMPs-HA_3 and TgNd6-TY_2 at the tip of the extruded conoid (Fig 6C), indicating that the two proteins might be spatially very close but part of distinct complexes, in agreement with the mass spectrometry data and the observation that CRMPa and CRMPb persist at the apical tip in the Nd9 mutant defective in RSA assembly (Figs 6D and EV5E–G). Nevertheless, this correlation is based on the detection of C-terminal markers which, in such high-resolution images, might not comprehensively reflect the spatial organization of the whole proteins. However, the co-localization analysis of co-expressed TgCRMPa-TY_2 and TgCRMPb-HA_3 (Fig EV5H–J) provided, as expected for members of the same complex, a more robust overlap than the one between TgCRMPs and TgNd6 (Fig 6B and C).

We next wondered whether CRMPs and Nd6 have a dynamic location during the invasion and checked if the apical dot labeled by CRMPs and Nd6 was maintained throughout the entire invasion process or limited to the pre-entry step. We fixed and immunostained parasites co-expressing TgCRMPs and TgNd6, at different time points during the invasion process, and used anti-RON5 antibodies to label the moving junction and mark the progress of the invasion. Interestingly, TgNd6 apical labeling was detected when the parasite started entering the host cell and remained visible throughout the entire process until the parasite was completely inside the host cell (Figs 6E and EV6). However, TgCRMPa apical signal vanished as soon as the moving junction is formed (Fig 6E). The same results were obtained for TgCRMPb (Fig EV6).

To sum up, CRMPs form a complex required to trigger exocytosis that is spatially located in close proximity but distinct from the RSA-associated Nd complex. Both Nd and CRMP complexes have different fates during the invasion, adding further support to a model where Nd and CRMP complexes play related but distinct roles in controlling rhoptry secretion at the exocytic site.

Discussion

Apicomplexan parasites have evolved highly specialized secretory organelles called rhoptries, which are key players in establishing successful infection. Rhoptry secretion is a complex process coupled with host membrane interaction and injection of materials into the host. The underlying mechanisms of this unique cell biological process remain largely unresolved, although hints regarding the exocytic step—the fusion among the rhoptry, AV, and parasite plasma membranes—have been recently obtained (Aquilini *et al*, 2021; Mageswaran *et al*, 2021; Martinez *et al*, 2022). In the present study, we took advantage of the relatively close evolutionary relationship between ciliates and apicomplexans, and in particular, their sharing unique mechanisms for regulated secretion (Aquilini *et al*, 2021), to identify new rhoptry secretion factors in *Toxoplasma*. A *Tetrahymena*-based *in silico* screening led us to the identification of a key rhoptry secretion complex comprising TgCRMPa, TgCRMPb, Tg247195, and Tg277910 proteins. Our data suggest that these novel factors link the recognition of the host cell to the activation of the rhoptry exocytic machinery.

We showed that the TgCRMPs are present at the apex of extracellular parasites, the site where the parasite contacts the host cell and discharges its rhoptry content. CRMPs, together with their partners, are membrane proteins containing domains known to interact with proteins and glycans. Protein structure predictions indicate that these domains might be exposed to the extracellular milieu, thus likely capable of interacting with host cell membranes, and we experimentally validated this topology in the case of *TgCRMPa*. This apical localization relies on the productive assembly of CRMPs. In addition, TgCMRPa apical signal is evident in both N- and C-terminally HA_3-tagged CRMPa lines, where two high-molecular-weight bands are detected by western blot, but not for the HA_3-miniAID-TgCRMPa (N-terminal tag) line, for which only the full-length protein seems to be present. These data suggest that it is mainly the processed form of TgCRMPa, the one accumulating at the apical dot in extracellular parasites. We also observed an abundant dispersed localization of CRMPs within the parasite cytosol, similar to what previously seen for all the Nd proteins (Aquilini *et al*, 2021), although in the case of CRMPs, the signal appears more apical, and partially overlapping with microneme proteins. CRMPs appear associated with vesicles since, by using the auxin degron system, the protein is degraded via proteasome only when the C-terminus, but not the N-terminus, is fused with the HA3-miniAID tag. This indicates that the N-terminal end of TgCRMPa is within the lumen of the transport vesicle and as such, protected from the effect of auxin. This suggests that CRMPs might be delivered to the apical end of the parasite upon vesicular

© 2022 The Authors

trafficking and secretion, a scenario deserving further investigation, as well as whether the vast majority of the proteins found in the cytosolic fraction play any role in addition to rhoptry exocytosis.

Both TgCRMPa and TgCRMPb partially co-localize with TgNd6 at the site of exocytosis. This association seems to be transient since it is evident only in extracellular parasites, and prior to the invasion. Once the parasite breaches the host membrane, the apical TgCRMPs staining disappears. In contrast, TgNd6 signal persists, suggesting that the factors regulating the RSA machinery and the process of membrane fusion might still be present at the parasite apex upon rhoptry secretion. Whether CRMPs loss is due to complete removal of the proteins from the exocytic site, cleavage of their C-terminal cytosolic tails, or post-entering interactions of the C-termini with cytosolic proteins masking the HA epitopes remains unknown. However, CRMPs behavior strongly argues for a function specifically at the time of rhoptry exocytosis. CRMPs and their partners Tg247195 and Tg277910 seem to not be part of the previously described Nd/NdP exocytic complex, also confirmed by a parallel study (preprint: Singer *et al*, 2022). However, we cannot exclude the existence of a dynamic/transient complex formed by CRMPs and Nd proteins at the time of rhoptry exocytosis. In contrast with the depletion of Nd9, we showed that removal of CRMPb does not affect the

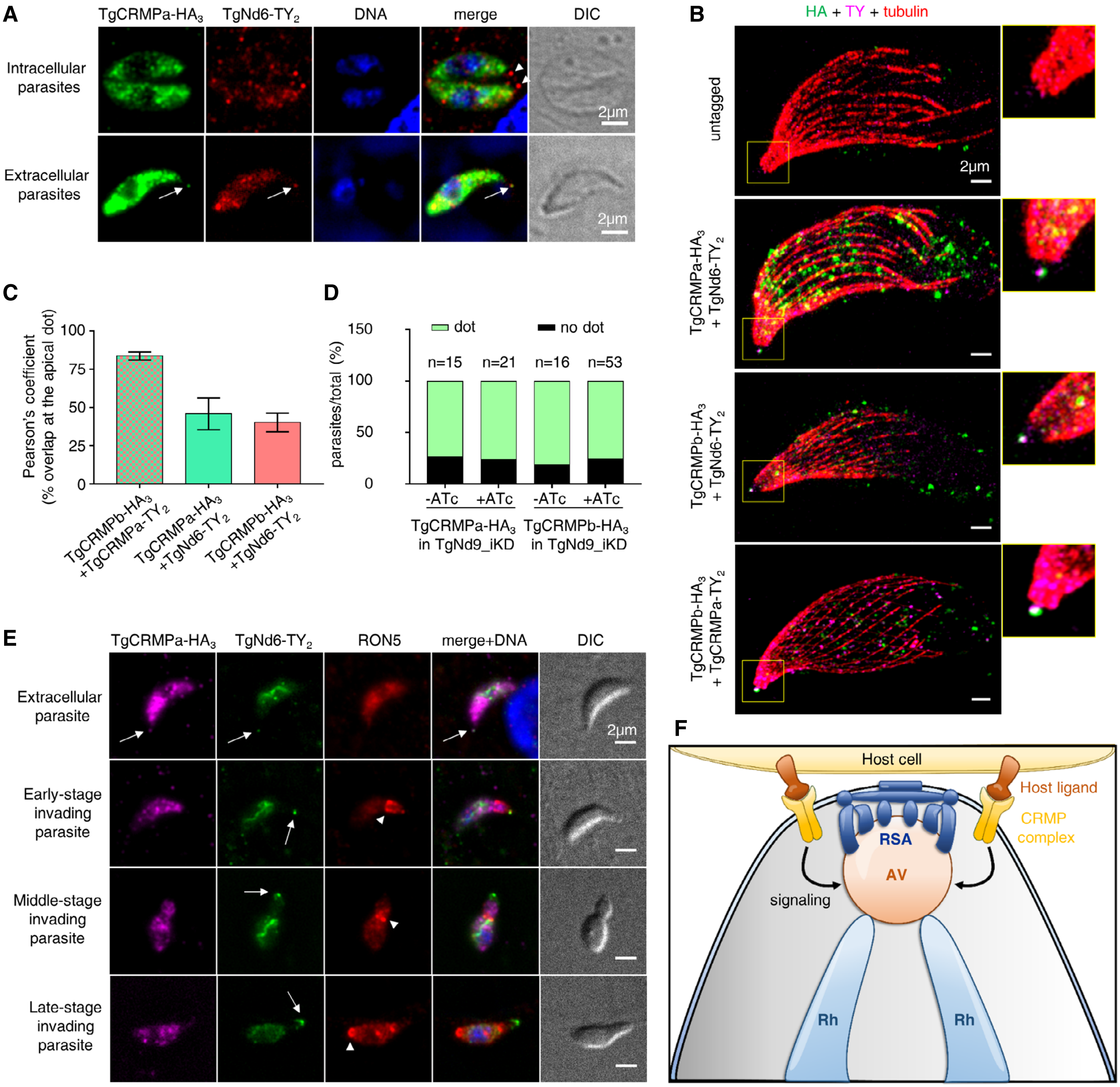

Figure 6.

Figure 6. CRMP and Nd complexes differentially regulate rhoptry secretion at the exocytic site in *Toxoplasma gondii*.

A Immunofluorescence images of intracellular (upper panel) and extracellular (lower panel) tachyzoites co-expressing TgCRMPa-HA_3 and TgNd6-TY_2. Extracellular parasites were incubated with host cell monolayers for 2 min prior to fixation. Parasites were stained with anti-HA and anti-TY Abs to label CRMPa and Nd6, respectively. Nd6, but not CRMPa, accumulates at the tachyzoite apex in intracellular parasites (arrowheads), while both proteins localize at the tip of the extruded conoid in extracellular parasites (arrows). DNA is labeled by Hoechst. Single focal planes are shown. DIC, differential interference contrast.

B Ultrastructure expansion microscopy of extracellular tachyzoites, either untagged or co-expressing TgCRMPa-HA_3/TgCRMPa-TY_2 and TgCRMPb-HA_3 together or in pairwise combination with TgNd6-TY_2. Parasites were treated with A23187 prior to fixation and preparation for U-ExM, and stained with anti-HA, anti-TY, and anti-α/β tubulin Abs to label CRMPs, Nd6/CRMPa, and microtubules, respectively. Shown are maximum-intensity projections of z-stack confocal images. CRMPs overlap with Nd6 at the tip of the extruded conoid (yellow selection). A magnified image of the apical tip of each parasite is shown on the right.

C The extent of co-localization between TgCRMPa-TY_2 and TgCRMPb-HA_3, and TgCRMPs-HA_3 with TgNd6-TY_2 in the apical dot shown in B. Pearson's correlation coefficient was measured using the Fiji-JACoP plugin. Values are expressed as mean ± SD. Three to five parasites per line were analyzed.

D Quantification of the dot pattern for TgCRMPa-HA_3 and TgCRMPb-HA_3 in TgNd9_iKD lines. CRMPs accumulation at the apical tip of extracellular parasites was measured as in Fig EV4A. TgCRMPs were still found in the apical dot in the absence of TgNd9 (+ATc). Numbers are expressed as the percentage of parasites showing (dot) or lacking (no dot) the tip accumulation of TgCRMPa and TgCRMPb. The number of parasites (*n*) analyzed for each line is reported at the top of the column.

E Immunofluorescence and DIC images of an extracellular parasite and parasites in early, middle, and late stages of host cell invasion. Parasites co-expressing TgCRMPa-HA_3 and TgNd6-TY_2 were incubated with host cell monolayers and fixed after 2, 3, and 5 min. Parasites were immunostained with anti-HA, anti-TY, and anti-RON5 Abs to label CRMPs, Nd6, and the moving junction, respectively. DNA is labeled by Hoechst. In contrast with TgNd6 (arrow), the apical accumulation of TgCRMPa observed in extracellular parasites (arrow) disappears upon entering the host, a step marked by the formation of the moving junction (arrowhead), and for the entire process. Non-specific anti-TY labeling of mitochondria was detected for both untagged and tagged lines (Fig EV6). Single focal planes are shown.

F Model depicting the proposed role for the TgCRMP complex in rhoptry exocytosis. Upon contacting the host cell via a host ligand, CRMPs participate in the activation of the signaling pathway targeting the AV-RSA system and leading to the fusion events required for the timely discharge of rhoptry contents. Rh, rhoptry; AV, apical vesicle; RSA, rhoptry secretory apparatus.

structural organization of the RSA, consistent with distinct roles for CRMPs and Nds/NdPs in the context of rhoptry secretion. This observation, together with the translocation of CRMPs to the exocytic site at the time of secretion, and their topology at the membrane with the putative host-binding domains exposed extracellularly, all support a model where CRMPs and their associated factors interact with surface ligands presented by the host cell. We propose that these interactions activate a signaling cascade within the parasite, leading to rhoptry discharge (Fig 6F). Intriguingly, removal of CRMPb induces slight changes in the shape and anchoring angle of the AV, while the RSA at the PPM is correctly assembled, which is an essential pre-requisite for efficient rhoptry secretion. Albeit the changes in the AV are relatively minor, they infer that there could be a direct or indirect connection of CRMPb to the AV which in turn could potentially regulate the rhoptry fusion apparatus. What remains unknown is whether these changes are a consequence of the signaling function of CRMPb, or if the loss of CRMPb locally affects the AV by, for example, impeding the recruitment of other factors essential for maintaining the vesicle well-shaped and fit for fusion.

Our "signaling-based" model is also supported by the fact that TgCRMPa and its ortholog PfCRMP1 are predicted to be GPCR-like proteins. Moreover, although there is no clear prediction for *Tetrahymena* TtCRMP1 and TtCRMP2 as GPCRs, they contain a GPCR-autoproteolysis inducing (GAIN) domain as found by HHpred analysis (score 81.6% for CRMP1 and 95.4% for CRMP2; Zimmermann *et al*, 2018). CRMPs cannot be considered *bona fide* GPCRs because they do not have the classical seven transmembrane domains of GPCRs (five predicted for *Tetrahymena* CRMPs, and nine for *Toxoplasma* and *Plasmodium* CRMPs), but they may be divergent forms that have maintained similar activities. GPCR is the largest family of membrane-bound receptors known to sense diverse extracellular stimuli and initiate signaling cascades within the cell cytosol to activate cellular responses. GPCRs are involved in nearly all biological processes and represent the favorite therapeutic target for many pathologies (Hauser *et al*, 2018). Few GPCRs are annotated in the *Toxoplasma* and *P. falciparum* genomes (ToxoDB.org; Fredriksson & Schiöth, 2005; Madeira *et al*, 2008), suggesting that they are highly divergent and therefore difficult to recognize in Apicomplexa. Because of CRMPs' potential host-cell-binding domains, they might be analogous to adhesion GPCRs, a sub-group of proteins with a large extracellular part containing structural modules typical of cell adhesion proteins (Yona *et al*, 2008; Liebscher *et al*, 2021). Adhesion GPCRs convert the stimulus derived from cell–cell contact into intracellular signaling via their C-termini, but many lacks identified activating ligands. Our data on the localization and topology of TgCRMPs support this scenario, in which the proteins' N-termini are exposed extracellularly to capture the signal, and the C-termini face the cell cytoplasm to transduce the signal for exocytosis. The N-terminal extensions of the apicomplexan CRMPs are larger than the *Tetrahymena* counterparts and contain, in addition to the EGF receptor domain, both lectin and Kringle domains. This difference in complexity may reflect the need to respond to different stimuli for triggering exocytosis. Ciliates must sense environmental changes to trigger exocytosis, while the response of the parasites depends on intimate cell–cell contacts: they must interact first with a host to ensure that rhoptry secretion is effective. Interestingly, while *Plasmodium* CRMP1 and CRMP2 are dispensable for merozoite invasion of red blood cells, they are required for sporozoite entry into the salivary glands (Thompson *et al*, 2007; Douradinha *et al*, 2011), a step recently shown to be dependent on rhoptry secretion (Ishino *et al*, 2019; Fernandes *et al*, 2022). These findings suggest that CRMPs evolved differently to adapt to diverse environments or hosts.

The localization of CRMPs at the apical tip is evident, while their intracellular distribution is less clear. TgCRMPa and TgCRMPb are visible as small dots likely corresponding to vesicles, within the cytosol of intracellular parasites. An apical gradient reminiscent of micronemes was occasionally observed, but CRMPs do not appear to co-localize extensively with microneme markers. Moreover, the hyperLOPIT (spatial proteomics method hyperplexed localization of organelle proteins by isotopic tagging) analysis predicts that CRMPs, Tg247195 and Tg277910, are associated with micronemes when using the TAGM-MCMC method, but this prediction was not

 © 2022 The Authors

supported by data obtained with TAGM-MAP analysis (Barylyuk *et al*, 2020). These different observations might be reconciled by the long-standing hypothesis of different subsets of micronemes (Kremer *et al*, 2013). Unfortunately, immunoelectron microscopy of TgCRMPa- and TgCRMPb-tagged lines was inconclusive, and thus did not further clarify the distribution of these proteins in intracellular parasites.

In this study we described CRMPs as novel secretory factors shared between ciliates and apicomplexans, providing further support to the existence of conserved machinery for secretion specific to Alveolata. Our previous work showed that the fusion machinery responsible for the discharge of secretory organelles is conserved between Apicomplexa and Ciliata (Nd/NdP proteins). Here, we extend such conservation to the putative signaling pathway leading to exocytosis. CRMPs represent a suitable target for new treatments against apicomplexan-related infections. Uncovering the host ligands for CRMPs, Tg247195 and Tg277910 proteins, as well as the signaling pathway downstream of their interaction, will greatly help to develop strategies for blocking rhoptry exocytosis and subsequent invasion, contributing further to fighting human infections caused by apicomplexans.

Materials and Methods

Tetrahymena culture conditions

Tetrahymena thermophila strains used in this work are the wild-type CU428.1 (Ctrl) and the mutants *Δfer2*, *Δ00442310* (*Δcrmp1*), and *Δ00637180* (*Δcrmp2*). Unless otherwise indicated, cells were grown overnight in SPP (2% proteose peptone, 0.1% yeast extract, 0.2% dextrose, and 0.003% ferric-EDTA) supplemented with 250 ug/ml penicillin G and 250 μg/ml streptomycin sulfate to medium density (1–3 × 10^5 cells/ml). For biolistic transformation, growing cells were subsequently starved in 10 mM Tris buffer, pH 7.4. Fed and starved cells were both kept at 30°C with agitation at 60 rpm. Culture densities were measured using the Neubauer chamber.

Homology searching and phylogenetic tree construction for *ferlin* genes

Toxoplasma gondii ferlin 2 protein sequence (TgFer2; TGME49_260470) was used as a query against translated open reading frame (ORF) coding sequences from the genomes of selected Ciliata and Apicomplexa species using the BLAST algorithm. Positive BLAST hits against TgFer2 query were those with an E value < 0.001 and score > 50, for which reciprocal BLAST against the genome containing the query sequence retrieved either the same sequence or an isoform of the sequence with a similar E value or lower. Once homologs of TgFer2 were identified by BLAST, the phylogenetic relationships of ferlins within ciliates and apicomplexans were determined by maximum-likelihood estimation. Homologs were aligned by MUSCLE and tree construction was performed by MEGA11 software (Tamura *et al*, 2021). The root of the tree was determined using *Toxoplasma gondii* and *Neospora caninum* ferlin 3 as an outgroup. Ciliata and Apicomplexa sequences were retrieved from either the http://ciliate.org or http://veupathdb.org databases, respectively. Identification numbers and E-values of the proteins used for tree construction are reported in Dataset EV4.

Coregulation data harvester (CDH) analysis

The identification of co-expressed genes for *Tetrahymena Fer2* (TTHERM_00886960), *Nd6* (TTHERM_00410160), *NdP1* (TTHERM_01287970), and *NdP2* (TTHERM_00498010) was performed by Coregulation Data Harvester (CDH) software (http://ciliate.org/index.php/show/CDH) as previously described (Tsypin & Turkewitz, 2017). A list of co-expressed genes, with homologs in *T. gondii* and *P. falciparum*, was obtained for each query. We then generated a cross-list by selecting genes shared at least by three queries, for which reciprocal BLAST toward ToxoDB and PlasmoDB databases retrieved *Toxoplasma* and *Plasmodium* homologous genes with an E-value of at least 10^{-4}, respectively (Dataset EV1).

Generation of *Tetrahymena* knockout strains

Δfer2, *Δcrmp1*, and *Δcrmp2* mutants were generated by replacing the macronuclear ORF of *Fer2* (TTHERM_00886960), *CRMP1* (TTHERM_00442310), and *CRMP2* (TTHERM_00637180), with the paromomycin (Neo4) drug resistance cassette (Mochizuki, 2008) via homologous recombination with the linearized vectors p00886960-Neo4, p00442310-Neo4, and p00637180-Neo4. To generate the knockout constructs, 500–800 bp fragments homologous to the genomic regions upstream (5′UTR) and downstream (3′UTR) of each ORF were PCR amplified with KOD HiFi polymerase (Merk), and cloned into SacI/PstI and XhoI/HindIII restriction sites, respectively, flanking the Neo4 cassette in the pNeo4 plasmid by Quick Ligation (New England, Biolabs Inc.). Specifically, for p00886960-Neo4, 707 bp (5′UTR) and 754 bp (3′UTR) homology regions (HRs) were amplified with primers ML4379/ML4380 and ML4381/ML4382, respectively; for p00442310-Neo4, 569 bp (5′UTR) and 721 bp (3′UTR) were amplified with primers ML3830/ML3831 and ML3832/ML3833, respectively; and for p00637180-Neo4, 727 bp (5′UTR) and 506 bp (3′UTR) were amplified with primers ML4283/ML4284 and ML4285/ML4286, respectively. Each construct was linearized by digestion with SacI and KpnI, and delivered into CU428.1 cells by biolistic transformation. Primers are listed in Table EV1.

Tetrahymena biolistic transformation

Tetrahymena CU428.1 was grown to mid-log phase and starved for 18–24 h in 10 mM Tris, pH 7.4. The biolistic transformation was performed with 20 μg synthetic DNA as described previously (Chilcoat *et al*, 1996; Cassidy-Hanley *et al*, 1997). Selection of positive transformants was initiated 5 h after bombardment by adding 120 μg/ml paromomycin sulfate and 1 μg/ml $CdCl_2$ to the cultures. Transformants were serially transferred 6×/week in increasing concentrations of the drug and decreasing concentrations of $CdCl_2$ (up to 3 mg/ml paromomycin and 0.2 μg/ml $CdCl_2$) for at least 6 weeks before further testing. Successful integration and replacement of all endogenous alleles at each genomic locus were tested by RT–PCR.

RT–PCR assessment of gene disruption in *Tetrahymena*

Overnight cultures of mid-log phase cells from each knockout strain were pelleted, washed once with 10 mM Tris pH 7.4, and total RNA was isolated using NucleoSpin RNA, Mini kit for RNA purification

© 2022 The Authors

(Macherey-Nagel), according to manufacturer's instructions. The cDNA synthesis from 2 to 3 μg of total RNA was performed using High-Capacity cDNA Reverse Transcription kit (Applied Biosystems). The cDNA was PCR amplified with GoTaq DNA Polymerase (Promega) to assay the presence of the corresponding transcripts (200–300 bp) in the knockout strains using primers listed in Table EV1. To confirm that equal amounts of cDNA were amplified, reactions with primers specific for β-tubulin 1 (BTU1) were run in parallel. At least three clones each for the knockout strains were tested.

Tetrahymena mucocysts secretion assay

Wild-type CU428.1 and knockout strains were grown to stationary phase (10^6 cells/ml) in 30 ml SPP for 48 h, and then concentrated into 500 μl loose pellet by centrifugation at 1,800 *g* for 3 min. Cells were stimulated with 165 μl of 25 mM dibucaine, vigorously mixed for 30 s, and diluted to 15 ml with 10 mM HEPES pH 7.4 and 5 mM $CaCl_2$. Samples were then centrifuged at 1,800 *g* for 3 min resulting in the formation of a cell pellet/flocculent bilayer. Quantification of exocytic competence was performed by measuring the ratio between flocculent layer and pellet volumes. At least three clones each for the knockout strains were tested.

Tetrahymena western blotting

Whole-cell lysates were collected from 5 × 10^4 cells from overnight cultures, washed once with 10 mM Tris pH 7.4, resuspended in 2× lithium dodecyl sulfate (LDS) sample buffer containing 40 mM DTT, and denatured at 95°C for 10 min. Proteins were resolved with the Novex NuPAGE Gel system (10% Bis-Tris gels, Invitrogen) and transferred to 0.45 μm PVDF membranes (Immobilon®-P, Millipore). Blots were blocked with 5% dried milk in 1× TNT (15 mM Tris, 140 mM NaCl, and 0.05% Tween 20, pH 8). The rabbit anti-Grl1 serum (Turkewitz *et al*, 1991) was diluted 1:2,000 in blocking solution. Proteins were visualized with anti-rabbit alkaline phosphatase (AP)-conjugated (Promega) diluted 1:7,500 and with BCIP/NBT Color development substrate (Promega). At least three clones each for the knockout strains were tested.

Tetrahymena immunofluorescence microscopy

Overnight cultures of mid-log phase *Tetrahymena* cells for CU428.1 (control), *Δ00886960* (*Δfer2*), *Δ00442310* (*Δcrmp1*), and *Δ00637180* (*Δcrmp2*) were washed once with 10 mM Tris pH 7.4, and fixed with 4% paraformaldehyde (PFA) in 50 mM HEPES pH 7.4 at room temperature. Cells were permeabilized with 0.1% Triton X-100 and blocking was performed with 1% bovine serum albumin (BSA) in TBS (25 mM Tris, 3 mM KCl, and 140 mM NaCl, pH 7.4); mucocyst proteins Grl3 were visualized with mouse mAb 5E9 (1:10; Cowan *et al*, 2005) followed by AlexaFluor488 goat anti-mouse antibody (1:450; Invitrogen), both diluted in 1% BSA. Cells were mounted in 30% glycerol/TBS and imaged on a Leica Thunder microscope, with a 100× oil objective NA = 1.4, equipped with the sCMOS 4.2MP camera, using Leica Application Suite X (LAS X) software (Leica Biosystems). Z-stacks were denoised, adjusted in brightness and contrast, and colored with the program Fiji (Schindelin *et al*, 2012). At least two clones each for the knockout strains were tested.

Toxoplasma culture conditions

Toxoplasma gondii RH tachyzoites (type I strain) lacking the *Ku80* gene (*ΔKu80*; Huynh & Carruthers, 2009) were used for genetic recombination. In particular, to generate inducible knockdown strains, we used either the *ΔKu80* line expressing the TATi transactivator for the TetOff system (*TATi-ΔKu80*; Sheiner *et al*, 2011), or the Tir-1 receptor for the auxin inducible degron system (miniAID; Brown *et al*, 2018; *ΔKu80* Tir-1). Parasites were routinely cultured in human foreskin fibroblasts (HFFs) monolayers (ATCC, CRL 1634) in standard medium (DMEM 5% fetal bovine serum (FBS), 2 mM glutamine, supplemented with penicillin and streptomycin from Gibco) at 37°C and 5% CO_2. For SeCreEt assays, parasites expressing the protein toxofilin fused with a Cre recombinase (Koshy *et al*, 2010) were cultured in mouse fibroblast cell line 10 T1/2, constitutively expressing a floxed red fluorescent protein DsRed (Koshy *et al*, 2010), used as Cre reporter cell line for assessing rhoptry secretion. Parasites used for immunoprecipitation experiments were cultured in vero cells (ATCC, CCL 81) with DMEM 3% FBS supplemented with glutamine, penicillin, and streptomycin. For positive selection via hypoxanthine–xanthine–guanine phosphoribosyl transferase (HXGPRT) drug resistance cassette, 25 μg/ml mycophenolic acid plus 50 μg/ml xanthine were added to the culture media; 2 μM pyrimethamine and 20 μM chloramphenicol (CHL) were used for selection with the dihydrofolate reductase thymidylate synthase (DHFR-TS) and chloramphenicol acetyl transferase (CAT) drug resistance cassettes, respectively. For negative selection via uracil phosphoribosyl transferase (UPRT) cassette, 5 μM fluorodeoxyuridine (FUDR) was added to the medium. To induce protein depletion in the iKD lines, 1 μg/ml anhydrotetracycline (ATc; Fluka 37919) or 0.5 mM auxin (indole-3-acetic acid; Sigma) was added to the medium for 24, 48, and 72 h, depending on the strain.

Generation of *Toxoplasma* tagged and knockdown strains

All *Toxoplasma*-related primers and RNA guides (gRNAs) used in this study are listed in Table EV1.

Genomic DNA was isolated using Wizard SV Genomic DNA Purification system (Promega). KOD HiFi Polymerase (Merk) and GoTaq DNA Polymerase (Promega) were used to amplify gene fragments for cloning strategy and colony screening PCRs, respectively.

TgCRMPa (TGGT1_261080) and *TgCRMPb* (TGGT1_292020) were C-terminally fused with a triple hemagglutinin (HA_3) tag followed by the chloramphenicol resistance cassette (CAT) for selection, in the *TATi-ΔKu80* line using CRISPR/Cas9. Briefly, gRNAs targeting the 3′UTR of the genes were generated by annealing primers ML3283/ML3284 and ML3279/ML3280, respectively. The annealed gRNAs were cloned in the pU6-Cas9-YFP plasmid using the BsaI restriction sites to generate pU6-TgCRMPa_gRNA1 and pU6-TgCRMPb_gRNA1. DNA fragments containing gene-specific homologous regions flanking the triple HA tag and the CAT cassette were amplified from pLIC_HA3_CAT vector (Huynh & Carruthers, 2009) using the primer pairs ML3287/ML3288 and ML3277/ML3278 for *TgCRMPa* and *TgCRMPb*, respectively, containing ~ 30 bp of homology to the 3′ and 3′UTR of the gene of interest. pU6-TgCRMPa_gRNA1 and pU6-TgCRMPb_gRNA1 plasmids and the corresponding donor DNAs were mixed prior to being transfected. The resulting lines were named TgCRMPa-HA_3 and TgCRMPb-HA_3.

© 2022 The Authors

Tg277910 was tagged with a triple HA tag (HA_3) at the C-terminal end of the protein in the *TATi-ΔKu80* line using the ligation independent cloning (LIC) strategy (Huynh & Carruthers, 2009) and chloramphenicol selection. Briefly, 1,485 bp fragment corresponding to the 3′ part of TGGT1_277910 gene minus the stop codon was amplified with primers ML4046/ML4047 and integrated in the pLIC_HA3-CAT (Huynh & Carruthers, 2009). The vector was then linearized with BaeI site prior to transfection. The tagged line was named Tg277910-HA_3.

Inducible knockdowns (iKDs) of *TgCRMPa*, *TgCRMPb*, and *Tg277910* were generated in TgCRMPa-HA_3, TgCRMPb-HA_3, and Tg277910-HA_3 lines, respectively, using pyrimethamine selection; the resulting strains were named *TgCRMPa*_iKD, *TgCRMPb*_iKD, and *Tg277910*_iKD. To create the iKD lines, the endogenous promotor of each gene was replaced by an anhydrotetracycline (ATc)-regulatable promotor (TetO7SAG4), preceded by the *DHFR* cassette, using CRISPR/Cas9, as described previously (Suarez *et al*, 2019). gRNAs targeting the 5′UTR of the genes were generated by annealing the primer pairs ML3342/ML3343, ML3338/ML3339, and ML3970/ML3971, for *TgCRMPa*, *TgCRMPb*, and *Tg277910*, respectively, and introduced in the BsaI site of pU6-Cas9-YFP to generate pU6-TgCRMPa_gRNA3, pU6-TgCRMPb_gRNA3, and pU6-Tg277910_gRNA1. Donor DNA fragments were obtained by amplifying the TetO7SAG4 promoter and the DHFR resistance cassette from the DHFR-TetO7SAG4 plasmid (Sheiner *et al*, 2011) with the following primers: ML3317/ML3318 (*TgCRMPa*_iKD), ML3315/ML3316 (*TgCRMPb*_iKD), and ML3966/ML3967 (*Tg277910*_iKD), respectively. Each pair of primers contains ~ 30 bp of homology to the 5′UTR and 5′ coding region of the gene. The gRNAs and donor DNAs were mixed prior to parasite transfection.

Auxin-inducible knockdown of *TgCRMPa* was generated in Tir-1-expressing line. *TgCRMPa* was either C-terminally fused with the miniAID sequence followed by a triple HA tag, or N-terminally fused with a triple HA tag followed by the miniAID sequence; the resulting lines were named TgCRMPa-miniAID-HA_3_iKD and HA_3-miniAID-TgCRMPa_iKD, respectively. For the C-terminal tagging of TgCRMPa, a fragment containing the miniAID sequence followed by the *HXGPRT* cassette was amplified from the plasmid pTUB8YFP-mAID-3 (Brown *et al*, 2017) with primers ML4909/ML4910, and mixed with the pU6-TgCRMPa_gRNA1. For the N-terminal tagging of TgCRMPa, we used a marker-free strategy. Integration of the tag (upstream of the MAR/Kringle sequences) at the endogenous locus was achieved by homologous recombination of a 1,000 bp DNA fragment (gBlock, Genescript) containing the triple HA tag followed by the miniAID sequence flanked by 207 and 265 bp of homology to the 5′ coding sequence (minus introns), including the signal peptide, and to the 3′ coding sequence prior to the Kringle/MAR domain, respectively. The 1,000 bp donor DNA was retrieved by EcoRI restriction from plasmid pUC57, and mixed with the pU6-TgCRMPa_gRNA34 Cas9 plasmid, obtained by cloning the annealed primers ML5327 and ML5328 in the BsaI site of pU6-Cas9-YFP.

To tag TgCRMPa with a triple HA at the N-terminus, we used again a marker-free strategy. Integration of the tag (downstream the MAR/Kringle domains) at the endogenous locus was achieved by homologous recombination of a 608 bp DNA fragment (gBlock, Genescript) containing a recodonized homologous sequence followed by a linker sequence and the triple HA tag, flanked by 200 bp each of homology to the 5′ and 3′ coding sequences of *TgCRMPa*. The 608 bp donor DNA was retrieved by EcoRI-EcoRV restriction from plasmid pUC57, and mixed with pU6-TgCRMPa_gRNA148 Cas9 plasmid, obtained by cloning the annealed primers ML5480 and ML5481 in the BsaI site of pU6-Cas9-YFP. The resulting line was named HA_3-TgCRMPa.

To tag TgCRMPa with a triple FLAG at the C-terminus, we used a marker-free strategy. Integration of the tag at the endogenous locus was achieved by homologous recombination of a 500 bp DNA fragment (gBlock, Genescript) containing the triple FLAG tag flanked by 185 and 228 bp of homology to the 3′ coding sequence and 3′UTR of *TgCRMPa*, respectively. The 500 bp donor DNA was amplified from the synthetic gBlock with primers ML3002/ML3003, and mixed with the pU6-Cas9-YFP plasmid containing pU6-TgCRMPa_gRNA1. *TgCRMPa* was fused with the triple FLAG tag in the *TATi-ΔKu80* and TgCRMPb-HA_3 lines, which were then used to generate *TgCRMPa* knockdown lines as described earlier for *TgCRMPa*_iKD. The lines generated from *TATi-ΔKu80* were named TgCRMPa-$FLAG_3$ and TgCRMPa-$FLAG_3$_iKD, those generated from TgCRMPb-HA_3 were named TgCRMPa-$FLAG_3$ + TgCRMPb-HA_3 and TgCRMPa-$FLAG_3$_iKD + TgCRMPb-HA_3.

C-terminal tagging of TgNd6 with double TY tag in TgCRMPa-HA_3 and TgCRMPb-HA_3 lines was obtained by inserting the coding sequence of TY_2, followed by the DHFR resistance cassette, immediately after the gene's stop codon in the *TgNd6* locus. The gRNA primers ML3129/ML3130 tagging the 3′UTR of *TgNd6* were annealed and then cloned into the pU6-Cas9-YFP plasmid using the BsaI restriction sites to generate pU6-TgNd6_CtgRNA. The 4,597 bp donor DNA was PCR amplified from the pLinker-2xTy-DHFR plasmid (Suarez *et al*, 2019) with primers ML4734/ML4735, and mixed with pU6-TgNd6_CtgRNA prior to transfection. The lines were named TgCRMPa-HA_3 + TgNd6-TY_2 and TgCRMPb-HA_3 + TgNd6-TY_2.

C-terminal TY_2 tagging of *TgCRMPa* in TgCRMPb-HA_3 line was performed as described earlier for TgNd6-TY_2. The 4,600 bp donor DNA was PCR amplified from the pLinker-2xTy-DHFR plasmid (Suarez *et al*, 2019) with primers ML5241/ML5242, and mixed with pU6-TgCRMPa_gRNA1 prior to transfection. The line was named TgCRMPb-HA_3 + TgCRMPa-TY_2.

C-terminal HA_3 tagging of *TgCRMPa* and *TgCRMPb* in *TgNd9*_iKD (Aquilini *et al*, 2021) was obtained as described earlier for TgCRMPa-HA_3 and TgCRMPb-HA_3 lines. To quantify rhoptry secretion using SeCreEt assays, the Toxofilin-Cre recombinase was introduced in *TATi-ΔKu80*, *TgCRMPa*_iKD, *TgCRMPb*_iKD, and *Tg277910*_iKD lines at the uracil phosphoribosyl transferase (UPRT) locus to generate *TATi-ΔKu80*_Toxofilin-Cre, *TgCRMPa*_iKD_Toxofilin-Cre, *TgCRMPb*_iKD_Toxofilin-Cre, and *Tg277910*_iKD_Toxofilin-Cre. Toxofilin-Cre (3,949 bp) was amplified from ToxofilinCre plasmid (Koshy *et al*, 2010) using primers ML3522/ML3523, containing ~ 30 bp of homology to the 5′ and 3′UTR of the *UPRT* gene and co-transfected with two specific single gRNAs cutting the 5′ (ML3445/ML3446) and 3′ (ML2087/ML2088) of the *UPRT* gene.

Toxoplasma transfection and screening of positive transformants

For *T. gondii* transfection, 60 μg of pLIC plasmid for Tg277910-HA_3 or 100 μl of purified digested fragments/PCR products (~ 5 μg) mixed with 15-20 μg of corresponding pU6-CAS9-YFP plasmids were introduced in 20×10^6 tachyzoites by electroporation, using

© 2022 The Authors

Electro Cell Manipulator 630 (BTX) with the following settings: 2.02 kV, 50 Ω, and 25 μF (Kim *et al*, 1993). After transfection, positive transformants were recovered by drug selection and clones were isolated by limiting dilution, or by fluorescence-activated cell sorting (FACS). Genomic DNA from isolated clones was purified as described earlier, and screened by PCR for correct integration with GoTaq DNA Polymerase (Promega). Alternatively, PCR screening of single clones directly from 96-well plates was performed with Phire™ Tissue Direct PCR master mix (Thermo Scientific) protocol, as previously described (Piro *et al*, 2020). The primers used to test correct integration are listed in Table EV1.

Homology searching and phylogenetic tree construction for *CRMP* genes in Apicomplexa

Toxoplasma gondii CRMPa (TGME49_261080) and CRMPb (TGME49_292020) protein sequences were used as queries against translated ORF coding sequences from the genomes of selected Apicomplexa (Dataset EV4) using the BLAST algorithm. Positive BLAST hits against CRMPa and CRMPb queries were those for which reciprocal BLAST against the genome containing the query sequence retrieved the same sequence with similar E-value or lower followed by the other TgCRMP. Once the homologs of TgCRMPa and b were identified by BLAST, the phylogenetic relationships of CRMPs within apicomplexans were determined as described earlier for ferlins. Apicomplexa sequences were retrieved from http://veupathdb.org databases. Identification numbers and E-values of the proteins used for tree construction are reported in Dataset EV4.

RT–PCR assessment of *Nd9* transcripts depletion in *toxoplasma*

TgNd9_iKD and TATi-*ΔKu80* parasites were treated 72 h with ATc and total RNA was isolated and reverse transcribed as mentioned earlier for *Tetrahymena* samples. Untreated parasites were analyzed in parallel. The cDNA was PCR amplified with GoTaq DNA Polymerase (Promega) to assay the presence of the corresponding *Nd9* transcripts (~ 250 bp) in the knockdown strain using primers listed in Table EV1. To confirm that equal amounts of cDNA were amplified, reactions with primers specific to TgGAPDH were run in parallel.

Toxoplasma rhoptry secretion assay

To assess parasites competence for rhoptry secretion, secreted Cre epitope-tagged (SeCreEt) parasites expressing the toxofilin-Cre fusion protein were generated as described earlier, and used to infect murine fibroblasts (cell line 10 T1/2) constitutively expressing a floxed red fluorescent protein DsRed. This mammalian Cre-reporter cell line is able to switch from DsRed to eGFP (enhanced green fluorescent protein) expression upon toxofilin-driven Cre-mediated recombination (Koshy *et al*, 2010). DsRed cells were grown to a density of 2×10^5 cells/ml and infected in the absence of ATc, with either ATc pre-treated (48, 24, and 72 h ATc incubation for *TgCRMPa*_iKD_Toxofilin-Cre, *TgCRMPb*_iKD_Toxofilin-Cre, and *Tg277910*_iKD_Toxofilin-Cre, respectively) or untreated tachyzoites, at a multiplicity of infection (MOI) of 3. One day post-infection, infected cells were trypsinized, and DsRed and eGFP fluorescence signals were measured by fluorescence-activated cell sorting (FACS). The numbers of DsRed and GFP-positive cells were used as measure of impaired or successful rhoptry secretion, respectively; the values were reported as fraction of GFP-positive cells over the total number of cells, and expressed as percentages. Each value was normalized to that of the control line (*TATi-ΔKu80*_Toxofilin Cre + ATc) arbitrarily fixed to 100%. Graphs show the mean of three independent experiments.

Toxoplasma invasion assay

For the quantification of invasion in *TgCRMPa*_iKD, *TgCRMPb*_iKD, and *Tg277910*_iKD lines, freshly egressed tachyzoites (5×10^6/coverslips) and ATc pre-treated (48, 24, and 72 h ATc incubation for *TgCRMPa*_iKD, *TgCRMPb*_iKD, and *Tg277910*_iKD, respectively) or untreated were added to HFF monolayers grown on coverslips in a 24-well plate, and let settle on ice for 20 min, prior to being transferred to a 38°C pre-heated water bath for 5 min, to allow invasion. Parasites were fixed with 4% PFA in Hank's Balanced Salt Solution (HBSS) for 20 min at room temperature, and incubated with 10% FBS/HBSS blocking solution. In order to distinguish intracellular from extracellular parasites, a dual-antibody staining was performed as previously described (Cerede *et al*, 2005). First, non-permeabilized extracellular parasites were stained using the mouse mAbs T41E5 anti-SAG1 (1:2,000; Couvreur *et al*, 1988) in 2% FBS/HBSS. Parasites and infected cells were then permeabilized with 0.1% saponin, and incubated again with blocking solution. Secondly, parasites were stained with rabbit anti-ROP1 antibodies (1:3,000; Lamarque *et al*, 2014) in 2% FBS/PBS to label intracellular parasites in parasitophorous vacuoles. Secondary antibody staining was performed with AlexaFluor594 goat anti-mouse (1:4,000) and AlexaFluor488 goat anti-rabbit (1:10,000) antibodies (Invitrogen). DNA was stained with 16 μM Hoechst, and coverslips were mounted onto microscope slides using Immunomount (Calbiochem). Intracellular parasites were counted in 20 fields/coverslip (n = 3 coverslips/experiment) with a Leica DM2500, 100× oil objective NA = 1.4, microscope (Leica Biosystems). The values were expressed as the number of intracellular parasites per field and normalized to that of the control line (*TATi-ΔKu80* -ATc) arbitrarily fixed to 100%. Graphs show the mean of three independent experiments.

Toxoplasma host cell attachment assay

The ability of TgCRMPa_iKD, TgCRMPb_iKD, and Tg277910_iKD tachyzoites to attach to host cells was assessed as previously described (Aquilini *et al*, 2021). HFFs grown on coverslips in a 24-well plate were fixed with cold 2% glutaraldehyde/PBS for 5 min at 4°C, washed three times with cold PBS, quenched with 100 mM cold glycine for 2 min, and washed three more times in PBS, and then kept in pre-heated DMEM 5% FBS. Coverslips were infected with 1×10^7 freshly egressed ATc-treated (48, 24, and 72 h ATc incubation for *TgCRMPa*_iKD, *TgCRMPb*_iKD, and *Tg277910*_iKD, respectively) and untreated parasites resuspended in 300 μl DMEM 5% FBS. Coverslips were also infected with parasites pre-treated with 20 μM BAPTA-AM (Sigma) and used as negative control for microneme-dependent attachment. Parasites were allowed to attach to host cells for 20 min at 37°C, carefully washed twice with DMEM 5% FBS, and then fixed with 4% PFA/PBS for 30 min. Parasites were incubated with 1.5% BSA/PBS blocking solution and

© 2022 The Authors

subsequently immunostained with mouse anti-SAG1 p30 hybridoma (1:50; Couvreur *et al*, 1988) followed by secondary staining with AlexaFluor488 goat anti-mouse antibodies (1:4,000; Invitrogen), both diluted in 0.15% BSA/PBS. DNA was stained with 16 μM Hoechst, and coverslips were mounted onto microscope slides using Immunomount (Calbiochem). Parasites were counted in 20 fields/coverslip (n = 3 coverslips/experiment) with a Leica DM2500, 100× oil objective NA = 1.4, microscope (Leica Biosystems). Attachment was reported as number of parasites found attached to the host cells per field and expressed as percentage. Values were normalized to that of the control line (iKD-ATc) arbitrarily fixed to 100%. Graphs show mean values of three independent experiments.

Toxoplasma **plaque assay**

TgCRMPa_iKD, TgCRMPb_iKD, and Tg277910_iKD tachyzoites pretreated with ATc for 48, 24, or 72 h, respectively, were used to infect HFF monolayers grown in 24-well plates and incubated with the corresponding drug. Auxin-inducible TgCRMPa_iKD tachyzoites were similarly used to infect HFF monolayers in 24-well plates in the presence of auxin. Untreated parasites were analyzed in parallel. Roughly 3,500 parasites were added in each well of the first row, then serial dilutions were performed by transferring ¼ of the parasites to the next well, and so on until the end of the plate. Lysis plaques formation was allowed for 7 days at 37°C and 5% CO_2. HFFs were then fixed with 4% PFA/PBS and stained with 1% Crystal-violet. Images of the lysis plaques were collected with Olympus MVX10 macro zoom microscope, equipped with a Zeiss MRM2 Camera. For ATc-inducible knockdowns, plaques area were measured using ImageJ (Schneider *et al*, 2012) and expressed as percentage. Values were normalized to that of the control line (*TATi-ΔKu80* -ATc) arbitrarily fixed to 100%. Graphs shows the mean of three independent experiments, including three technical replicates each.

Toxoplasma **replication assay**

In vitro growth of intracellular parasites was performed as previously described (Aquilini *et al*, 2021). HFF monolayers grown on coverslips were infected with 2 × 10^5 freshly egressed parasites, pre-treated with ATc (48, 24, and 72 h ATc incubation for *TgCRMPa*_iKD, *TgCRMPb*_iKD, and *Tg277910*_iKD, respectively), or untreated. Invasion was allowed for 2 h, HFFs were then washed five times with HBSS to remove extracellular parasites incapable of entering host cells, and intracellular parasites were let replicate for 24 h at 37°C. Cells were then fixed using 4%PFA/PBS and permeabilized with 0.1% Triton X-100; parasites were immunostained with mouse mAbs T41E5 anti-SAG1 (1:2,000; Couvreur *et al*, 1988) and AlexaFluor488 goat anti-mouse (1:4,000; Invitrogen) antibodies diluted in 2% FBS/PBS. DNA was stained with 16 μM Hoechst, and coverslips were mounted onto microscope slides using Immunomount (Calbiochem). The number of vacuoles containing 2, 4, 6, 8, 16, or 32 parasites was counted using a Leica DM2500, 100× oil objective NA = 1.4, microscope (Leica Biosystems), and expressed as a percentage. A total of 200 vacuoles/coverslips (n = 3 coverslips/experiment) were analyzed. Graphs show the means of two and three independent experiments for TgCRMPa_iKD and TgCRMPb_iKD, and Tg277910_iKD, respectively, including three technical replicates each.

Toxoplasma **egress assay**

To assess the egress of *T. gondii* tachyzoites from host cells, HFF monolayers grown on coverslips were infected with 1 × 10^5 parasites and incubated at 37°C for 30 h. Egress was induced by stimulating microneme secretion and parasite motility with 3 μM A23187 for 8 min. Parasites were fixed with 4% PFA/PBS, permeabilized with 0.1% Triton X-100, incubated with 10% FBS/PBS blocking solution, and stained with mouse anti-GRA3 hybridoma (1:100; Achbarou *et al*, 1991b) and rabbit anti-GAP45 (1:9,000; Frenal *et al*, 2014) antibodies, followed by AlexaFluor488 goat anti-mouse (1:4,000) and AlexaFluor594 goat anti-rabbit (1:4,000) antibodies (Invitrogen), respectively. Upon egress, the PVM is ruptured and GRA3 proteins are released from the PV space into the extracellular milieu. Thus, egress events were analyzed by quantifying the presence of GRA3 staining in intact and ruptured PVs, 200 vacuoles/coverslip were analyzed (n = 3 coverslips/experiment) with a Leica DM2500, 100× oil objective NA = 1.4, microscope (Leica Biosystems). Values were reported as the fraction of ruptured vacuoles over the total number of vacuoles observed, and were expressed as percentages. Values were normalized to that of the control line (*TATI-ΔKu80* -ATc) arbitrarily fixed to 100%. Graphs show the means of three independent experiments for TgCRMPa_iKD and TgCRMPb_iKD, and two independent experiments for Tg277910_iKD.

Toxoplasma **microneme secretion assay**

The extent of microneme secretion in TgCRMPa_iKD, TgCRMPb_iKD, and Tg277910_iKD lines was measured by evaluating the release of the TgMIC2 processed form in the supernatant. Freshly egressed, ATc-treated (48, 24, and 72 h ATc incubation for *TgCRMPa*_iKD, *TgCRMPb*_iKD and *Tg277910*_iKD, respectively) and untreated parasites were harvested by centrifugation at 600 *g*, washed twice in pre-heated intracellular buffer (5 mM NaCl, 142 mM KCL, 1 mM MgCl2 2 mM EGTA, 5.6 mM glucose, and 25 mM HEPES, pH 7.2), and resuspended in DMEM (minus FBS) with or without 500 μM propranolol (Sigma; P0884). Parasites were then incubated at 37°C for 20 min to induce the secretion of microneme contents into the supernatant. Supernatants were separated from parasite pellets by centrifugation at 2,000 *g* for 5 min at 4°C. Pellets were washed once in PBS and supernatants were additionally cleared by centrifugation at 4,000 *g* for 5 min. One-tenth and one-fifth of the total pellets and supernatants were subjected to SDS–PAGE and western blotting, respectively; full-length (~ 115 kDa) and processed TgMIC2 (~ 100 kDa) proteins were detected with mouse anti-MIC2 hybridoma (1:2; Achbarou *et al*, 1991a) and horseradish peroxidase (HRP)-conjugated goat anti-mouse (1:10,000; Jackson Immuno Research) secondary antibodies. TgGRA3 proteins were used as a loading control and detected with rabbit anti-GRA3 primary (1:500; Achbarou *et al*, 1991b) and anti-rabbit alkaline-phosphatase (AP)-conjugated (1:7,500; Promega) secondary antibodies. Proteins were visualized with BCIP/NBT Color development (Promega) or Clarity Max™ Western ECL (Bio-Rad) substrates. One representative experiment is shown for the iKD lines.

© 2022 The Authors

Toxoplasma immunofluorescence microscopy

Unless otherwise specified, immunofluorescence assays (IFAs) of intracellular parasites were performed as previously described (El Hajj *et al*, 2008). Briefly, coverslips containing infected HFF monolayers were fixed with 4% PFA/PBS for 30 min at room temperature. Cells were washed three times with PBS, permeabilized with 0.15% Triton X-100/PBS for 10 min, and then saturated with 10% FBS/PBS blocking solution for 1 h. Proteins were stained with primary antibodies for 1 h, followed by six washes with PBS and secondary staining with proper fluorophore-conjugated antibodies for 1 h. Antibodies were diluted in 2% FBS/PBS. HA_3-tagged proteins were visualized with rat anti-HA 3F10 (1:1,000; Roche; 11867460001) or rabbit anti-HA (1:5,000; Abcam; ab9110) and AlexaFluor488 goat anti-rat (1:2,000) or goat anti-rabbit (1:10,000; Invitrogen) antibodies; TgARO, TgAMA1, TgMIC2, TgGAMA, and TgPLP1 were visualized with rabbit anti-ARM(ARO; 1:1,000; Mueller *et al*, 2013), rabbit anti-AMA1 folded (1:5,000; Lamarque *et al*, 2014), mouse anti-MIC2 hybridoma (1:50; Achbarou *et al*, 1991a), rabbit anti-GAMA (1:500; Huynh & Carruthers, 2016), and rabbit anti-PLP1 (1:500; Roiko & Carruthers, 2013) primary antibodies, respectively, together with AlexaFluor594 goat anti-rabbit (1:4,000) or goat anti-mouse (1:4,000; Invitrogen) secondary antibodies.

For detecting the apical accumulation of TgCRMPa-HA_3 and TgCRMPb-HA_3, either alone or in pairwise combination with TgNd6-TY_2, 5×10^6 parasites/condition were added to HFF monolayers grown on coverslips in a 24-well plate, and let settle on ice for 20 min prior to being transferred to a 38°C preheated water bath to allow invasion. According to the experimental design, parasites were fixed with 4% PFA/PBS after 2, 3, and 5 min incubation at 38°C. Fixation was allowed for 30 min at room temperature prior to permeabilization with 0.1% Triton X-100, blocking with 10% FBS/PBS, and antibody staining of extracellular and invading parasites. Rat anti-HA 3F10 (1:1,000) primary (Roche; 11867460001) and AlexaFluor488 goat anti-rat secondary (1:2,000; Invitrogen) antibodies diluted in 2% FBS/PBS were used to visualize triple HA-tagged TgCRMPa and TgCRMPb alone, in *TATi-ΔKu80*, TgCRMPa_iKD, and TgNd9_iKD backgrounds. The co-staining of TgCRMPa-HA_3 and TgCRMPb-HA_3 with TgNd6-TY_2 was performed with rabbit anti-HA (1:5,000; Abcam; ab9110) and mouse anti-TY hybridoma (1:100; Bastin *et al*, 1996) primary antibodies, followed by AlexaFluor488 goat anti-rabbit (1:10,000) and AlexaFluor594 goat anti-mouse (1:4,000) secondary antibodies (Invitrogen), diluted in 2% FBS/PBS. Intracellular parasites co-expressing TgCRMPs and TgNd6-TY were similarly stained.

For the time-course experiment during the invasion, the co-staining of TgCRMPa-HA_3 and TgCRMPb-HA_3 with TgNd6-TY_2 with primary antibodies was performed as described earlier, followed by AlexaFluor647 goat anti-rabbit (1:2,000) and AlexaFluor488 goat anti-mouse highly cross-adsorbed (HCA; 1:4,000; Invitrogen) secondary antibodies diluted in 10% FBS/PBS; to visualize the moving junction, parasites were incubated again with 10% FBS/PBS blocking solution, and stained with rat anti-RON5 (1:200; Besteiro *et al*, 2009) followed by AlexaFluor594 goat anti-rat HCA (1:2,000; Invitrogen) antibodies. The use of highly cross-adsorbed (HCA) secondary antibodies limited cross-reactivity. The co-staining of TgCRMPb-HA_3 and TgCRMPa-TY_2, in intracellular and extracellular parasites, was performed with rabbit anti-HA (1:5,000; Abcam; ab9110) and mouse anti-TY hybridoma (1:100; Bastin *et al*, 1996) primary antibodies, followed by AlexaFluor488 goat anti-rabbit (1:10,000) and AlexaFluor647 goat anti-mouse (1:2,000) secondary antibodies (Invitrogen), diluted in 2% FBS/PBS, respectively.

DNA was stained with 16 µM Hoechst, and coverslips were mounted onto microscope slides using Immunomount (Calbiochem). Imaging was performed either with a Leica Thunder microscope, with a 100× oil objective NA = 1.4, equipped with the sCMOS 4.2MP camera, using Leica Application Suite X (LAS X) software (Leica Biosystems), or Zeiss Axioimager Z2 epifluorescence microscope, with a 100× oil objective NA = 1.4, equipped with the CMOS Orca Flash 4.0 (Hammamatsu) camera, using Zen software (Zeiss, Intelligent Imaging Innovations), or Zeiss LSM880 confocal microscope equipped with Airyscan detector, with a 63× oil objective NA = 1.4, using Zen Black software (Zeiss, Intelligent Imaging Innovations). Images of single focal planes and z-stacks were uniformly denoised, adjusted in brightness and contrast, and colored with the program Fiji (Schindelin *et al*, 2012). Images were collected at the Montpellier Ressources Imagerie (MRI) facility of the University of Montpellier.

Co-localization analysis of *Toxoplasma* CRMPs with Nd6 and microneme proteins

To estimate the extent of co-localization, the Fiji-JACoP plugin was used to calculate Pearson's correlation coefficient (PCC; Bolte & Cordelieres, 2006). The overlap between CRMPa and CRMPb signals, as well as that of CRMPs with Nd6 at the apical dot of A23187-treated extracellular parasites, was measured by creating a binary mask of the selected area covering the entire volume of the parasite at the extreme apex. PCC was calculated by setting the threshold to the estimated value of the background. Z-stacks of three to five parasites for each line were analyzed. The overlap between CRMPb-HA_3 and the microneme proteins AMA1, MIC2, GAMA, and PLP1 in intracellular parasites was measured as described above. Untagged parasites, equally stained with anti-HA Abs in pairwise combination with the four anti-MICs Abs, were analyzed in parallel to estimate the background noise. Untagged parasites were also stained with anti-AMA1 and anti-MIC2 antibodies to measure the overlap between the two microneme proteins. A total of 20–32 parasites were analyzed for each pair of antibodies.

Western blotting of *Toxoplasma* proteins

For western blotting of whole-cell lysates, ~ 10^7 freshly egressed tachyzoites/condition were washed once in PBS and resuspended in 100°C Laemmli SDS or lithium dodecyl sulfate (LDS) sample buffer supplemented with 10-40 mM dithiothreitol (DTT; Sigma). Epitope-tagged TgCRMPa, TgCRMPb, Tg277910, and related proteins were resolved with either the Bio-Rad Gel system (10% gel: 10% acrylamide/bis, 0.4 M Tris–HCl pH 8.8, 0.1% SDS, and 0.1% APS, TEMED) or the Novex NuPAGE Gel system (3–8% Tris-Acetate gels, Invitrogen) and transferred either to 0.45 µm nitrocellulose (Amersham Protron, GE Healthcare Life Science) or 0.45 µm PVDF (Immobilon®-P, Millipore) membranes. Blots were blocked either with 5% dried milk or 3% BSA in TNT (15 mM Tris, 140 mM NaCl, and 0.05% Tween 20, pH 8). The rat anti-HA 3F10 (1:1,000; Roche; 11867460001), mouse anti-ROP5 T53E2 (1:500; El Hajj *et al*, 2007),

 © 2022 The Authors

mouse anti-MIC2 hybridoma (1:2; Achbarou *et al*, 1991a), rabbit anti-FLAG (1:5,000; Sigma, F7425), and mouse anti-TY hybridoma (1:200; Bastin *et al*, 1996) were diluted in blocking solution. FLAG-tagged proteins were visualized with horseradish peroxidase (HRP)-conjugated donkey anti-rabbit (1:10,000; Jackson Immuno Research); and HA-tagged proteins with anti-rat alkaline-phosphatase (AP)-conjugated (1:10,000; Invitrogen), TY-tagged proteins, TgMIC2 and TgROP5, were visualized with anti-mouse alkaline-phosphatase (AP)-conjugated (1:7,500; Promega) secondary antibodies. Proteins were visualized with BCIP/NBT Color development (Promega) or Clarity Max™ Western ECL (Bio-Rad) substrates. ECL-based detection was performed with Chemidoc System (Bio-Rad).

Immunoprecipitation and co-immunoprecipitation of *Toxoplasma* proteins

Immunoprecipitation (IP) of TgCRMPa-HA_3 and TgCRMPb-HA_3 and co-immunoprecipitation (co-IP) of TgCRMPa-$FLAG_3$ and TgCRMPb-HA_3 were performed from 500×10^6 tachyzoites, resuspended in 1 ml cold lysis buffer (1% NP40, 50 mM Tris pH7.4, 150 mM NaCl, and 4 mM EDTA), supplemented with protease inhibitor cocktail tablets (Roche; 11867460001), and gently mixed for 4 h at 4°C. Lysates were cleared by centrifugation at 13,500 *g* for 30 min at 4°C, and supernatants were transferred in a 1.5 ml tube for overnight incubation with proper antibody-conjugated magnetic beads. IP supernatants for mass-spectrometry analysis were incubated with 50 µl anti-HA beads (Pierce; R88836) while, those for co-IP, were split into two tubes and separately incubated with 50 µl anti-HA beads (Pierce; R88836) and 50 µl anti-FLAG M2 beads (Sigma; M8823). The beads were then washed five times with lysis buffer and resuspended in 100°C Laemmli SDS or lithium dodecyl sulfate (LDS) sample buffer, supplemented with 10–40 mM dithiothreitol (DTT; Sigma). Untagged parasites were treated in parallel. Prior to mass spectrometry analysis, protein samples were loaded on a 3–8% gel for SDS–PAGE and stained with Coomassie Blue R-250 solution to verify protein enrichment upon immunoisolation of the protein of interest. Protein samples from co-IP experiments were resolved with the Novex NuPAGE Gel system (3–8% Tris-Acetate gels, Invitrogen) and subjected to western blotting as described earlier. TgCRMPa-$FLAG_3$ and TgCRMPb-HA_3 proteins were co-stained with the rat anti-HA 3F10 (1:1,000; Roche; 11867460001) and rabbit anti-FLAG (1:5,000; Sigma; F7425) primary antibodies, in combination with anti-rabbit alkaline-phosphatase (AP)-conjugated (1:7,500; Promega) and horseradish peroxidase (HRP)-conjugated donkey anti-rat (1:10,000; Jackson Immuno Research) secondary antibodies, respectively. TgROP5 was used as a negative control for co-IP experiments and searched in the clear lysates (before incubation with beads) and IP eluates with mouse anti-ROP5 T53E2 (1:500; El Hajj *et al*, 2007) antibodies, followed by anti-mouse alkaline-phosphatase (AP)-conjugated (1:7,500; Promega) secondary antibodies. Proteins were visualized with BCIP/NBT Color development (Promega) or Clarity Max™ Western ECL (Bio-Rad) substrates. ECL-based detection was performed with Chemidoc system (Bio-Rad).

Mass spectrometry analysis of *Toxoplasma* proteins

Proteins were digested in gel (2 bands per sample) as previously described (Skorupa *et al*, 2013). Peptides were loaded onto a 25 cm reversed-phase column (75 mm inner diameter, Acclaim Pepmap 100® C18, Thermo Fisher Scientific) and separated with an Ultimate 3000 RSLC system (Thermo Fisher Scientific) coupled to a Q Exactive HFX (Thermo Fisher Scientific). MS/MS analyses were performed in a data-dependent mode. Full scans (375–1,500 m/z) were acquired in the Orbitrap mass analyzer with a resolution of 60,000 at 200 m/z. For the full scans, 3e6 ions were accumulated within a maximum injection time of 60 ms. The 12 most intense ions with charge states ≥ 2 were sequentially isolated (1e5) with a maximum injection time of 45 ms and fragmented by higher-energy collisional dissociation (HCD) in the collision cell (normalized collision energy of 28) and detected in the Orbitrap analyzer at a resolution of 30,000. Raw spectra were processed using the MaxQuant (Cox & Mann, 2008) using standard parameters with a match between runs (Cox *et al*, 2011). MS/MS spectra were matched against the UniProt Reference proteomes of *Toxoplasma gondii* and humans (respectively, Proteome ID UP000001529, v2019_11 and UP000005640, v2020_01) and 250 frequently observed contaminants as well as reversed sequences of all entries (MaxQuant contaminant database). Statistical analyses were done using Perseus on intensity data (Tyanova *et al*, 2016).

Freeze-fracture and transmission electron microscopy of *Toxoplasma* and *Tetrahymena* strains

Tetrahymena Δcrmp1 cells were grown overnight in 30 ml SPP to mid-log phase density, harvested by centrifugation at 1,800 *g* for 3 min, and washed once with 10 mM Tris pH 7.4. Cells were resuspended in 5 ml 20 mM phosphate buffer (prepared from 0.1 M stock pH 7.1, containing 0.1 M sodium phosphate monobasic and 0.1 M sodium phosphate dibasic at 1:4 ratio) and fixed by adding 5 ml 3% glutaraldehyde (1.5% final conc.) diluted in 30 mM phosphate buffer, for 4 h at room temperature with gentle mixing. Three milliliter of fixed *Tetrahymena* cells were withdrawn from the total amount, pelleted to remove the fixative solution, and resuspended in 30 mM phosphate buffer. *Toxoplasma* TgCRMPa_iKD parasites were cultured for 48 h in the presence of ATc; freshly egressed tachyzoites were harvested by centrifugation at 650 *g* for 5 min, and fixed as solid pellets with 2.5% glutaraldehyde in 0.1 M phosphate buffer for 2 h at room temperature. Upon removal of the fixative, pellets were maintained in 30% glycerol diluted in 0.1 M phosphate buffer. Fixed *Tetrahymena* and *Toxoplasma* samples were subjected to freeze-fracture as previously described (Aquilini *et al*, 2021). Briefly, cells were quickly frozen in liquid nitrogen and fractured in a Bal-Tec BAF060 apparatus. The fracture surface replica was obtained by evaporating platinum at a 45° angle (~ 3.2 nm thick) and carbon at a 90° angle (~ 25 nm thick), respectively. Replicas were washed in 6.5% sodium hypochlorite, rinsed first in chloroform solution (2:1, v/v; this step was skipped for *Tetrahymena* replicas), and then in distilled water prior to mounting on copper grids. Images were acquired with a Jeol 1200 EXII transmission electron microscope FLAGat the Electron Microscopy Platform of the University of Montpellier, adjusted in brightness and contrast, with the program Fiji (Schindelin *et al*, 2012).

Tachyzoites preparation for Cryo-ET of *Toxoplasma*

RH strain *T. gondii* tachyzoites (both wild-type and CRMPb_iKD mutant) were cultivated as described earlier (Suarez *et al*, 2019)

© 2022 The Authors

with minor modifications. Tachyzoites were grown within monolayer human foreskin fibroblasts (HFF – ATCC, CRL 1634) in culture media composed of DMEM-10 (Thermo Fisher, Cat# 10313039) supplemented with 5% fetal calf serum, 2 mM glutamine, and a cocktail of penicillin–streptomycin. Extracellular parasites freshly egressed were isolated and concentrated in culture media before freezing 4 μl of this suspension (~ 4 × 10^6 tachyzoites) in each EM grid. For the purpose of tomogram reconstruction, the cell suspension was pre-mixed with 10 nm colloidal gold fiducials (Ted Pella, Cat# 15703) prior to freezing. The CRMPb_iKD parasites were pretreated with 1 μM ATc (Sigma-Aldrich, Cat# 37919) for 48 h before freezing.

Cryo-electron tomography (cryo-ET) and subtomogram averaging

Cryo-ET and subtomogram averaging were performed as previously described (Mageswaran *et al*, 2021). Briefly, projection images were recorded on a Thermo Fisher Titan Krios G3i 300 keV field-emission gun cryogenic electron microscope equipped with a K3 direct electron detector (Gatan Inc., Pleasanton, CA, USA) using SerialEM software (Mastronarde, 2005). The camera was operated in the electron-counted mode and images were dose fractionated at 10 frames per second. Images were motion corrected using the AlignFrames function in IMOD software package (Kremer *et al*, 1996). Volta phase plate (Danev *et al*, 2014; Fukuda *et al*, 2015) and Gatan Imaging Filter (Gatan Inc., Pleasanton, CA, USA; Krivanek *et al*, 1995) with a slit width of 20 eV were used to increase the contrast. The imaging workflow is as follows: cells were initially assessed at lower magnifications for ice thickness and plasma membrane integrity, following which tilt series were collected with a span of 120° (−60° to +60°; bi-directional or dose-symmetric scheme) with 2° increments accounting for a total dosage of 120–140 e−/Å^2 per tilt series. Tilt series were collected at 33,000× magnification with a corresponding pixel size of 2.65 Å (it is noteworthy that a part of the CRMPb_iKD dataset was collected on a replacement K3 camera that reported a slightly increased pixel size of 2.72 Å). Each tilt series had a fixed defocus value between 1 and 3 μm under focus. Our in-house automated computation pipeline (built on functions from the IMOD software package) was used to align tilt series and reconstruct tomograms; the 10 nm colloidal gold served as fiducials for the alignment procedure. IMOD's slicer program was used to visualize tomograms. After orienting the 3D volume and sectioning through the desired location, we generally averaged a few slices above and below to enhance contrast. Subtomogram averaging was performed on the RSA (including the AV) of *CRMPb_iKD* cells using PEET (Nicastro *et al*, 2006). These features were first located in several parasite tomograms by manually inspecting their apical region, and subtomograms (a.k.a. particles) were extracted from each in a manually pre-oriented fashion. Subsequently, these particles were computationally aligned over four iterations, each one using reduced angular and translational search parameters compared to the previous. A template was generated for the first alignment iteration by computing an initial average from the manually pre-oriented particles; the template for each of the subsequent iterations was computed by averaging the aligned particles from the corresponding previous iteration. An alignment mask encompassing the RSA (along with the plasma membrane) and the anterior region of the AV was used. After noticing a conserved eightfold rotational symmetry for the RSA along the longitudinal axis (one that is roughly perpendicular to the patch of plasma membrane where the RSA is anchored), we generated eightfold more particles by iteratively rotating each particle and repeated our alignment and averaging procedure. We thus enhanced the signal-to-noise ratio, which allowed us to resolve the finer details of the RSA ultrastructure in *CRMPb_iKD*. In total, we used 41 unique particles, which contributed 328 particles while exploiting the eightfold symmetry, to generate the final average.

Quantifications of Cryo-ET data, statistics, and reproducibility

We obtained a total of 100 tomograms (over 7 days spanning two independent imaging sessions) for wild-type *T. gondii* and 59 tomograms (over 7 days spanning three independent imaging sessions) for *CRMPb_iKD T. gondii*, each dataset from multiple frozen grids. The wild-type dataset is the same as the one previously published (Mageswaran *et al*, 2021). Each of the quantifications (described below) was performed on a randomly chosen subset of these tomograms that resolved the feature of interest. In the case of the wild-type, quantifications were performed again independent of the previous quantifications in Mageswaran *et al* (2021) to control for small discrepancies in measurements that could arise from different users or different attempts at the same analysis. Parasites showed some flattening on the EM grid, likely due to blotting. However, this flattening did not reflect the shape of the AV or the RSA. Flattening could have caused relatively small displacements of these features but their organizational patterns in the wild-type and CRMPb_iKD cells were evident despite the presence of such potential noise.

AV measurements

AV_{dist} (AV anchoring distance): the shortest distance measured from the parasite apex to the AV membrane. The apex is defined as the central position on the PPM where the RSA is anchored.

Ψ' (a measure of AV offset under the RSA): the angle formed between the orthogonal from the apex and the line connecting the AV centroid to the apex.

AV dimensions: Each AV was described by approximating it to a 2D ellipse using only two axes for simplicity (instead of describing it in 3D using three axes). The longest axis for each vesicle in 3D was marked as the major axis (labeled as AV_{maj}) while the shortest axis orthogonal to the major axis and intersecting it at the centroid was marked as the minor axis (labeled as AV_{min}). In other words, one of the central slices of the AV (representing an ellipse approximation) was used to describe the vesicle. Eccentricity (or Ecc) is calculated as (1-b2/a2)1/2, where "a" is the semi-major axis and "b" is the semi-minor axis.

Sample size: 22 tomograms each of wild-type and *CRMPb_iKD* cells were used for the above quantifications, except for Ψ', which used 37 tomograms for each sample.

Quantifications were performed using models generated in IMOD slicer. Models were exported as text files, parsed, and analyzed using Python 3.8 or 3.9. Numpy, Matplotlib, and Seaborn libraries were used for plotting. Boxplots show the distribution of measurements; in each dataset, the lower and upper boundaries of the box represent the first and third quartiles (Q1 and Q3), whiskers extend to 1.5 times the interquartile range (Q3–Q1) below and above Q1

© 2022 The Authors

and Q3, and points outside (diamonds) are regarded as outliers (NOTE: the whiskers on either side are shortened if there are no data points spanning the previously calculated whisker length). The horizontal divider within the box represents the median. The boxplots are overlaid with swarmplots, each data point representing a measurement from a tomogram. Jointplots are a combination of a bivariate scatterplot and two marginal univariate kernel density estimate plots (a.k.a. probability density plots). Mann–Whitney U test, which is a non-parametric alternative for unpaired Student's t-test (available within Python's Scipy package), was used to calculate the P-values. Actual P-values are presented in the plots for values < 0.1. For values > 0.1, they are replaced with n.s (not significant).

Toxoplasma conoid extrusion assay

To induce conoid extrusion in triple HA-tagged TgCRMPa and TgCRMPb lines, 150–300 µl of freshly egressed parasites were added to poly-D-lysine-coated coverslips pre-heated at 37°C in a 24-well plate. The plate was centrifuged at 400 g for 1 min to attach the parasites to the coverslips, and the medium was carefully removed. Three hundred microliters of pre-heated HEPES buffer (274 mM NaCl, 10 mM KCl, 2 mM Na_2HPO_4, 11 mM glucose, and 42 mM HEPES, pH 7.05) supplemented with 5 mM $CaCl_2$ and 5 µM A23187 ionophore were added to each coverslip. HEPES buffer without A23187 was added to control coverslips where spontaneous conoid extrusion may occur. The plate was incubated at 37°C for 8 min to stimulate A23187-dependent conoid extrusion.

Parasites were fixed with 4% PFA/PBS for 30 min at room temperature upon removal of the buffer, quenched with 100 mM glycine/PBS for 10 min, washed with PBS, permeabilized with 0.1% Triton X-100/PBS, and incubated with 10% FBS/PBS blocking solution for 1 h. Parasites staining was performed with rat anti-HA 3F10 (1:1,000; Roche; 11867460001) and AlexaFluor488-conjugated goat anti-rat (1:2,000; Invitrogen) antibodies. DNA was stained with 16 µM Hoechst, and coverslips were mounted onto microscope slides using Immunomount (Calbiochem). Imaging was performed with a Leica Thunder microscope, with a 100× oil objective NA = 1.4, equipped with the sCMOS 4.2MP camera, using Leica Application Suite X (LAS X) software (Leica Biosystems). Z-stacks were denoised, adjusted in brightness and contrast, and colored with the program Fiji (Schindelin *et al*, 2012).

Ultrastructure expansion microscopy (U-ExM) of *Toxoplasma* tachyzoites

This technique allows a near-native expansion of cell structures, enabling parasites to stretch up to four times their initial size. Freshly egressed tachyzoites, co-expressing either TgCRMPa-HA_3 and TgCRMPb-HA_3 with TgNd6-TY_2, or TgCRMPa-TY_2 with TgCRMPb-HA_3, were treated with A23187 as described earlier, to induce extrusion of the conoid. The untagged line was processed in parallel. Upon fixation with 4% PFA/PBS and quenching with 100 mM glycine/PBS, PBS-washed coverslips were transferred to a 12-well plate and protein cross-linking was allowed for 5 h at 37°C with 0.7% formaldehyde and 2% acrylamide diluted in PBS. Parasites were then embedded in a gel made of a monomer solution (19% sodium acrylate, 10% acrylamide, and 0.1% N, N′-methylenebisacrylamide in PBS) supplemented with 10% TEMED and 10% APS; gelation proceeded for 1 h at 37°C. Gels containing the parasites were detached from coverslips while dipped in a denaturation buffer (200 mM SDS, 200 mM NaCl, and 50 mM Tris, pH 9), and heated at 70°C for 90 min to denature proteins. Gels were expanded in ddH_2O overnight at room temperature and then shrank in PBS for antibody incubation. Staining of TgCRMPa-HA_3 and TgCRMPb-HA_3 with TgNd6-TY_2 was performed with rabbit anti-HA (1:2,500; Abcam; ab9110) and mouse anti-TY hybridoma (1:50; Bastin *et al*, 1996) primary antibodies, together with guinea pig anti-αtubulin (1:200; AA345; University of Geneva) and guinea pig anti-βtubulin (1:200; AA344; University of Geneva) antibodies, used to label subpellicular microtubules. Secondary antibody staining was performed with AlexaFluor647 goat anti-rabbit (1:1,000), AlexaFluor594 goat anti-mouse (1:2,000), and AlexaFluor488 goat anti-guinea pig HCA (1:1,500) secondary antibodies (Invitrogen). Gels were incubated with primary and secondary antibodies for 3 h at 37°C each time. Antibodies were diluted in 2% BSA/PBS and washed after each antibody incubation was performed with PBS-containing 0.1% Tween20. Gels were subjected to the second round of expansion in ddH_2O overnight prior to microscopy imaging. Expanded parasites were imaged with Zeiss LSM880 confocal microscope equipped with Airyscan detector, with a 63× oil objective NA = 1.4, using Zen Black software (Zeiss, Intelligent Imaging Innovations). Z-stacks were denoised, adjusted in brightness and contrast, colored, and processed to obtain maximum intensity projections, with the program Fiji (Schindelin *et al*, 2012).

Data availability

The mass spectrometry proteomics data have been deposited to the ProteomeXchange Consortium via the PRIDE (Perez-Riverol *et al*, 2019) partner repository with the dataset identifiers PXD031161 (http://www.ebi.ac.uk/pride/archive/projects/PXD031161) and PXD031164 (http://www.ebi.ac.uk/pride/archive/projects/PXD031164).

Expanded View for this article is available online.

Acknowledgments
We thank Sebastian Lourido for the pU6-Universal plasmid, Dominique Soldati-Favre for providing the anti-ARM (ARO) antibodies and pLinker-2xTy-DHFR plasmid, Nicolas Dos Santos Pacheco for helping in setting up the Ultrastructure Expansion Microscopy, Anita Koshy for the toxofilin-Cre plasmid, and Helen Blau's lab for the Cre reporter DSred cell line. We thank Veronique Richard and Frank Godiard of the MEA platform, University of Montpellier, for their assistance with electron microscopy and Pilar Ruga Fahy of the Pôle Facultaire de Microscopie Ultrastructurale, in Geneva for the preparation of freeze-fracture replicas. We are also grateful to Elodie Jublanc, Vicky Diakou, and the imaging facility MRI at the University of Montpellier, part of the national infrastructure France-BioImaging supported by the French National Research Agency (ANR-10-INBS-04, «Investments for the future»), and Christophe Duperray of the MRI-Cytometry at the Institute for Regenerative Medicine and Biotherapy for their assistance and technical support. Mass spectrometry experiments were carried out using the facilities of the Montpellier Proteomics Platform (PPM, BioCampus Montpellier). We thank Stefan Steimle for his technical assistance with the Krios G3i cryogenic electron microscope; the Singh Center for Nanotechnology and the Beckman Center for Cryogenic Electron Microscopy at the University of Pennsylvania for hosting and supporting the use of the Titan Krios. Dr Maryse Lebrun is an INSERM

© 2022 The Authors

researcher. This work was supported by the Laboratoire d'Excellence (LabEx) (ParaFrap ANR-11-LABX-0024), and European Research Council (ERC advanced grant number 833309 KissAndSpitRhoptry) to M.L.; by the FACCTS (France and Chicago Collaborating in the Sciences) to A.P.T. and M.L.; by NIH GM105783 to A.P.T.; by a David and Lucile Packard Fellowship for Science and Engineering (2019-69645) and a Pennsylvania Department of Health FY19 Health Research Formula Fund to Y.-W.C; and by NIH R01 AI112427 to B.S. D.S. and M.M.C. are supported by the European Research Council (ERC) under the European Union's Horizon 2020 research and innovation program under Grant agreement no. 833309 to M.L.

Author contributions

Maryse Lebrun: Conceptualization; supervision; funding acquisition; visualization; writing – original draft; writing – review and editing. **Daniela Sparvoli:** Conceptualization; investigation; visualization; writing – original draft; writing – review and editing. **Jason Delabre:** Investigation; visualization; writing – review and editing. **Diana Marcela Penarete-Vargas:** Investigation. **Shrawan Kumar Mageswaran:** Investigation; writing – review and editing. **Lev M Tsypin:** Investigation. **Justine Heckendorn:** Investigation. **Liam Theveny:** Investigation. **Marjorie Maynadier:** Investigation. **Marta Mendonça Cova:** Investigation. **Laurence Berry-Sterkers:** Investigation. **Amandine Guérin:** Investigation. **Jean-Francois Dubremetz:** Investigation. **Serge Urbach:** Investigation. **Boris Striepen:** Funding acquisition; writing – review and editing. **Aaron P Turkewitz:** Supervision; funding acquisition; writing – review and editing. **Yi-Wei Chang:** Supervision; funding acquisition.

Disclosure and competing interest statement

The authors declare that they have no conflict of interest.

References

Achbarou A, Mercereau-Puijalon O, Autheman JM, Fortier B, Camus D, Dubremetz JF (1991a) Characterization of microneme proteins of *Toxoplasma gondii*. *Mol Biochem Parasitol* 47: 223–233

Achbarou A, Mercereau-Puijalon O, Sadak A, Fortier B, Leriche MA, Camus D, Dubremetz JF (1991b) Differential targeting of dense granule proteins in the parasitophorous vacuole of *Toxoplasma gondii*. *Parasitology* 3: 321–329

Adams JC, Tucker RP (2000) The thrombospondin type 1 repeat (TSR) superfamily: diverse proteins with related roles in neuronal development. *Dev Dyn* 218: 280–299

Aquilini E, Cova MM, Mageswaran SK, Dos Santos Pacheco N, Sparvoli D, Penarete-Vargas DM, Najm R, Graindorge A, Suarez C, Maynadier M *et al* (2021) An Alveolata secretory machinery adapted to parasite host cell invasion. *Nat Microbiol* 6: 425–434

Barylyuk K, Koreny L, Ke H, Butterworth S, Crook OM, Lassadi I, Gupta V, Tromer E, Mourier T, Stevens TJ *et al* (2020) A comprehensive subcellular atlas of the toxoplasma proteome via hyperLOPIT provides spatial context for protein functions. *Cell Host Microbe* 28: 752–766.e9

Bastin P, Bagherzadeh Z, Matthews KR, Gull K (1996) A novel epitope tag system to study protein targeting and organelle biogenesis in *Trypanosoma brucei*. *Mol Biochem Parasitol* 77: 235–239

Beisson J, Lefort-Tran M, Pouphile M, Rossignol M, Satir B (1976) Genetic analysis of membrane differentiation in paramecium. Freeze-fracture study of the trichocyst cycle in wild-type and mutant strains. *J Cell Biol* 69: 126–143

Besteiro S, Michelin A, Poncet J, Dubremetz J-F, Lebrun M (2009) Export of a *Toxoplasma gondii* rhoptry neck protein complex at the host cell membrane to form the moving junction during invasion. *PLoS Pathog* 5: e1000309

Besteiro S, Dubremetz JF, Lebrun M (2011) The moving junction of apicomplexan parasites: a key structure for invasion. *Cell Microbiol* 13: 797–805

Blumenschein TM, Friedrich N, Childs RA, Saouros S, Carpenter EP, Campanero-Rhodes MA, Simpson P, Chai W, Koutroukides T, Blackman MJ *et al* (2007) Atomic resolution insight into host cell recognition by *Toxoplasma gondii*. *EMBO J* 26: 2808–2820

Bolte S, Cordelieres FP (2006) A guided tour into subcellular colocalization analysis in light microscopy. *J Microsc* 224: 213–232

Briguglio JS, Kumar S, Turkewitz AP (2013) Lysosomal sorting receptors are essential for secretory granule biogenesis in Tetrahymena. *J Cell Biol* 203: 537–550

Brown KM, Long S, Sibley LD (2017) Plasma membrane association by N-acylation governs PKG function in *Toxoplasma gondii*. *mBio* 8: e00375-17

Brown KM, Long S, Sibley LD (2018) Conditional knockdown of proteins using auxin-inducible degron (AID) fusions in *Toxoplasma gondii*. *Bio Protoc* 8: e2728

Burrell A, Marugan-Hernandez V, Moreira-Leite F, Ferguson DJP, Tomley F, Vaughan S (2021) Cellular electron tomography of the apical complex 1 in the apicomplexan 2 parasite *Eimeria tenella* shows a highly organised gateway for regulated secretion. *PLoS Pathog* 18: e1010666

Carruthers VB, Sibley LD (1997) Sequential protein secretion from three distinct organelles of *Toxoplasma gondii* accompanies invasion of human fibroblasts. *Eur J Cell Biol* 73: 114–123

Carruthers VB, Sibley LD (1999) Mobilization of intracellular calcium stimulates microneme discharge in *Toxoplasma gondii*. *Mol Microbiol* 31: 421–428

Cassidy-Hanley D, Bowen J, Lee JH, Cole E, VerPlank LA, Gaertig J, Gorovsky MA, Bruns PJ (1997) Germline and somatic transformation of mating *Tetrahymena thermophila* by particle bombardment. *Genetics* 146: 135–147

Cerede O, Dubremetz JF, Soete M, Deslee D, Vial H, Bout D, Lebrun M (2005) Synergistic role of micronemal proteins in *Toxoplasma gondii* virulence. *J Exp Med* 201: 453–463

Chilcoat ND, Melia SM, Haddad A, Turkewitz AP (1996) Granule lattice protein 1 (Grl1p), an acidic, calcium-binding protein in *Tetrahymena thermophila* dense-core secretory granules, influences granule size, shape, content organization, and release but not protein sorting or condensation. *J Cell Biol* 135: 1775–1787

Coleman BI, Saha S, Sato S, Engelberg K, Ferguson DJP, Coppens I, Lodoen MB, Gubbels MJ (2018) A member of the ferlin calcium sensor family is essential for *Toxoplasma gondii* rhoptry secretion. *mBio* 9: e01510-18

Couvreur G, Sadak A, Fortier B & Dubremetz JF (1988) Surface antigens of *Toxoplasma gondii*. *Parasitology* 97 (Pt 1): 1–10

Cowan AT, Bowman GR, Edwards KF, Emerson JJ, Turkewitz AP (2005) Genetic, genomic, and functional analysis of the granule lattice proteins in *Tetrahymena secretory* granules. *Mol Biol Cell* 16: 4046–4060

Cox J, Mann M (2008) MaxQuant enables high peptide identification rates, individualized p.p.b.-range mass accuracies and proteome-wide protein quantification. *Nat Biotechnol* 26: 1367–1372

Cox J, Neuhauser N, Michalski A, Scheltema RA, Olsen JV, Mann M (2011) Andromeda: a peptide search engine integrated into the MaxQuant environment. *J Proteome Res* 10: 1794–1805

Danev R, Buijsse B, Khoshouei M, Plitzko JM, Baumeister W (2014) Volta potential phase plate for in-focus phase contrast transmission electron microscopy. *Proc Natl Acad Sci U S A* 111: 15635–15640

 © 2022 The Authors

Douradinha B, Augustijn KD, Moore SG, Ramesar J, Mota MM, Waters AP, Janse CJ, Thompson J (2011) Plasmodium cysteine repeat modular proteins 3 and 4 are essential for malaria parasite transmission from the mosquito to the host. *Malar J* 10: 71

Dubremetz JF (1998) Host cell invasion by *Toxoplasma gondii*. *Trends Microbiol* 6: 27 – 30

El Hajj H, Lebrun M, Fourmaux MN, Vial H, Dubremetz JF (2007) Inverted topology of the *Toxoplasma gondii* ROP5 rhoptry protein provides new insights into the association of the ROP2 protein family with the parasitophorous vacuole membrane. *Cell Microbiol* 9: 54 – 64

El Hajj H, Papoin J, Cérède O, Garcia-Réguet N, Soête M, Dubremetz J-F, Lebrun M (2008) Molecular signals in the trafficking of *Toxoplasma gondii* protein MIC3 to the micronemes. *Eukaryot Cell* 7: 1019 – 1028

Fernandes P, Loubens M, Le Borgne R, Marinach C, Ardin B, Briquet S, Vincensini L, Hamada S, Hoareau-Coudert B, Verbavatz J-M *et al* (2022) The AMA1-RON complex drives *Plasmodium sporozoite* invasion in the mosquito and mammalian hosts. *PLoS Pathog* 18: e1010643

Fredriksson R, Schiöth HB (2005) The repertoire of G-protein-coupled receptors in fully sequenced genomes. *Mol Pharmacol* 67: 1414 – 1425

Frenal K, Marq JB, Jacot D, Polonais V, Soldati-Favre D (2014) Plasticity between MyoC- and MyoA-glideosomes: an example of functional compensation in *Toxoplasma gondii* invasion. *PLoS Pathog* 10: e1004504

Friedrich N, Santos JM, Liu Y, Palma AS, Leon E, Saouros S, Kiso M, Blackman MJ, Matthews S, Feizi T *et al* (2010) Members of a novel protein family containing microneme adhesive repeat domains act as sialic acid-binding lectins during host cell invasion by apicomplexan parasites. *J Biol Chem* 285: 2064 – 2076

Froissard M, Keller AM, Cohen J (2001) ND9P, a novel protein with armadillo-like repeats involved in exocytosis: physiological studies using allelic mutants in paramecium. *Genetics* 157: 611 – 620

Fukuda Y, Laugks U, Lučić V, Baumeister W, Danev R (2015) Electron cryotomography of vitrified cells with a Volta phase plate. *J Struct Biol* 190: 143 – 154

Ghosh S, Kennedy K, Sanders P, Matthews K, Ralph SA, Counihan NA, de Koning-Ward TF (2017) The Plasmodium rhoptry associated protein complex is important for parasitophorous vacuole membrane structure and intraerythrocytic parasite growth. *Cell Microbiol* 19: 1 – 16

Gilbert LA, Ravindran S, Turetzky JM, Boothroyd JC, Bradley PJ (2007) *Toxoplasma gondii* targets a protein phosphatase 2C to the nuclei of infected host cells. *Eukaryot Cell* 6: 73 – 83

Gogendeau D, Keller AM, Yanagi A, Cohen J, Koll F (2005) Nd6p, a novel protein with RCC1-like domains involved in exocytosis in *Paramecium tetraurelia*. *Eukaryot Cell* 4: 2129 – 2139

Guerin A, Corrales RM, Parker ML, Lamarque MH, Jacot D, El Hajj H, Soldati-Favre D, Boulanger MJ, Lebrun M (2017) Efficient invasion by Toxoplasma depends on the subversion of host protein networks. *Nat Microbiol* 2: 1358 – 1366

Haddad A, Turkewitz AP (1997) Analysis of exocytosis mutants indicates close coupling between regulated secretion and transcription activation in Tetrahymena. *Proc Natl Acad Sci U S A* 94: 10675 – 10680

Hakimi M-A, Olias P, Sibley LD (2017) Toxoplasma effectors targeting host signaling and transcription. *Clin Microbiol Rev* 30: 615 – 645

Hanssen E, Dekiwadia C, Riglar DT, Rug M, Lemgruber L, Cowman AF, Cyrklaff M, Kudryashev M, Frischknecht F, Baum J *et al* (2013) Electron tomography of *Plasmodium falciparum* merozoites reveals core cellular events that underpin erythrocyte invasion. *Cell Microbiol* 15: 1457 – 1472

Hauser AS, Chavali S, Masuho I, Jahn LJ, Martemyanov KA, Gloriam DE, Babu MM (2018) Pharmacogenomics of GPCR drug targets. *Cell* 172: 41 – 54.e19

Huynh M-H, Carruthers VB (2009) Tagging of endogenous genes in a *Toxoplasma gondii* strain lacking Ku80. *Eukaryot Cell* 8: 530 – 539

Huynh M-H, Carruthers VB (2016) A *Toxoplasma gondii* ortholog of plasmodium GAMA contributes to parasite attachment and cell invasion. *mSaphere* 1: e00012-16

Ishino T, Murata E, Tokunaga N, Baba M, Tachibana M, Thongkukiatkul A, Tsuboi T, Torii M (2019) Rhoptry neck protein 2 expressed in *Plasmodium sporozoites* plays a crucial role during invasion of mosquito salivary glands. *Cell Microbiol* 21: e12964

Kemp LE, Yamamoto M, Soldati-Favre D (2012) Subversion of host cellular functions by the apicomplexan parasites. *FEMS Microbiol Rev* 37: 607 – 631

Kessler H, Herm-Gotz A, Hegge S, Rauch M, Soldati-Favre D, Frischknecht F, Meissner M (2008) Microneme protein 8: a new essential invasion factor in *Toxoplasma gondii*. *J Cell Sci* 121: 947 – 956

Kim K, Soldati D, Boothroyd JC (1993) Gene replacement in *Toxoplasma gondii* with chloramphenicol acetyltransferase as selectable marker. *Science* 262: 911 – 914

Koshy AA, Fouts AE, Lodoen MB, Alkan O, Blau HM, Boothroyd JC (2010) Toxoplasma secreting Cre recombinase for analysis of host-parasite interactions. *Nat Methods* 7: 307 – 309

Kremer JR, Mastronarde DN, McIntosh JR (1996) Computer visualization of three-dimensional image data using IMOD. *J Struct Biol* 116: 71 – 76

Kremer K, Kamin D, Rittweger E, Wilkes J, Flammer H, Mahler S, Heng J, Tonkin CJ, Langsley G, Hell SW *et al* (2013) An overexpression screen of *Toxoplasma gondii* Rab-GTPases reveals distinct transport routes to the micronemes. *PLoS Pathog* 9: e1003213

Krivanek OL, Friedman SL, Gubbens AJ, Kraus B (1995) An imaging filter for biological applications. *Ultramicroscopy* 59: 267 – 282

Kumar S, Briguglio JS, Turkewitz AP (2014) An aspartyl cathepsin, CTH3, is essential for proprotein processing during secretory granule maturation in Tetrahymena thermophila. *Mol Biol Cell* 25: 2444 – 2460

Lamarque MH, Roques M, Kong-Hap M, Tonkin ML, Rugarabamu G, Marq JB, Penarete-Vargas DM, Boulanger MJ, Soldati-Favre D, Lebrun M (2014) Plasticity and redundancy among AMA-RON pairs ensure host cell entry of Toxoplasma parasites. *Nat Commun* 5: 4098

Lebrun M, Carrthers VB, Cesbron-Delauw M-F (2020) Toxoplasma secretory proteins and their roles in parasite cell cycle and infection. In *Toxoplasma gondii: The Model Apicomplexan; Perspectives and Methods*, Weiss LM, Kim K (eds), pp 607 – 704. Amsterdam: Elsevier

Liebscher I, Cevheroğlu O, Hsiao C-C, Maia AF, Schihada H, Scholz N, Soave M, Spiess K, Trajković K, Kosloff M *et al* (2021) A guide to adhesion GPCR research. *FEBS J* https://doi.org/10.1111/febs.16258

Madeira L, Galante PAF, Budu A, Azevedo MF, Malnic B, Garcia CRS (2008) Genome-wide detection of serpentine receptor-like proteins in malaria parasites. *PLoS One* 3: e1889

Mageswaran SK, Guérin A, Theveny LM, Chen WD, Martinez M, Lebrun M, Striepen B, Chang Y-W (2021) *In situ* ultrastructures of two evolutionarily distant apicomplexan rhoptry secretion systems. *Nat Commun* 12: 4983

Martinez M, Chen WD, Cova MM, Molnár P, Mageswaran SK, Guérin A, John ARO, Lebrun M, Chang Y-W (2022) Rhoptry secretion system structure and priming in plasmodium falciparum revealed using *in situ* cryo-electron tomography. *Nat Microbiol* 7: 1230 – 1238

Mastronarde DN (2005) Automated electron microscope tomography using robust prediction of specimen movements. *J Struct Biol* 152: 36 – 51

Meissner M, Schluter D, Soldati D (2002) Role of toxoplasma gondii myosin a in powering parasite gliding and host cell invasion. *Science* 298: 837 – 840

Mi H, Ebert D, Muruganujan A, Mills C, Albou L-P, Mushayamaha T, Thomas PD (2021) PANTHER version 16: a revised family classification, tree-based

classification tool, enhancer regions and extensive API. *Nucleic Acids Res* 49: D394 – D403

Mochizuki K (2008) High efficiency transformation of Tetrahymena using a codon-optimized neomycin resistance gene. *Gene* 425: 79 – 83

Mondragon R, Frixione E (1996) Ca(2+)-dependence of conoid extrusion in toxoplasma gondii tachyzoites. *J Eukaryot Microbiol* 43: 120 – 127

Mueller C, Klages N, Jacot D, Santos JM, Cabrera A, Gilberger TW, Dubremetz J-F, Soldati-Favre D (2013) The Toxoplasma protein ARO mediates the apical positioning of rhoptry organelles, a prerequisite for host cell invasion. *Cell Host Microbe* 13: 289 – 301

Nicastro D, Schwartz C, Pierson J, Gaudette R, Porter ME, McIntosh JR (2006) The molecular architecture of axonemes revealed by cryoelectron tomography. *Science* 313: 944 – 948

Nichols BA, Chiappino ML, O'Connor GR (1983) Secretion from the rhoptries of *Toxoplasma gondii* during host-cell invasion. *J Ultrastruct Res* 83: 85 – 98

Nishimura K, Fukagawa T, Takisawa H, Kakimoto T, Kanemaki M (2009) An auxin-based degron system for the rapid depletion of proteins in nonplant cells. *Nat Methods* 6: 917 – 922

Patthy L, Trexler M, Váli Z, Bányai L, Váradi A (1984) Kringles: modules specialized for protein binding. Homology of the gelatin-binding region of fibronectin with the kringle structures of proteases. *FEBS Lett* 171: 131 – 136

Perez-Riverol Y, Csordas A, Bai J, Bernal-Llinares M, Hewapathirana S, Kundu DJ, Inuganti A, Griss J, Mayer G, Eisenacher M *et al* (2019) The PRIDE database and related tools and resources in 2019: improving support for quantification data. *Nucleic Acids Res* 47: D442 – D450

Pietrzyk-Brzezinska AJ, Bujacz A (2020) H-type lectins – structural characteristics and their applications in diagnostics, analytics and drug delivery. *Int J Biol Macromol* 152: 735 – 747

Piro F, Carruthers VB, Di Cristina M (2020) PCR screening of *Toxoplasma gondii* single clones directly from 96-well plates without DNA purification. *Methods Mol Biol* 2071: 117 – 123

Plattner H, Miller F, Bachmann L (1973) Membrane specializations in the form of regular membrane-to-membrane attachment sites in Paramecium. A correlated freeze-etching and ultrathin-sectioning analysis. *J Cell Sci* 13: 687 – 719

Possenti A, Di Cristina M, Nicastro C, Lunghi M, Messina V, Piro F, Tramontana L, Cherchi S, Falchi M, Bertuccini L *et al* (2022) Functional characterization of the thrombospondin-related paralogous proteins rhoptry discharge factors 1 and 2 unveils phenotypic plasticity in toxoplasma gondii rhoptry exocytosis. *Front Microbiol* 13: 899243

Riglar DT, Richard D, Wilson DW, Boyle MJ, Dekiwadia C, Turnbull L, Angrisano F, Marapana DS, Rogers KL, Whitchurch CB *et al* (2011) Super-resolution dissection of coordinated events during malaria parasite invasion of the human erythrocyte. *Cell Host Microbe* 9: 9 – 20

Roiko MS, Carruthers VB (2013) Functional dissection of *Toxoplasma gondii* perforin-like protein 1 reveals a dual domain mode of membrane binding for cytolysis and parasite egress. *J Biol Chem* 288: 8712 – 8725

Satir B (1977) Dibucaine-induced synchronous mucocyst secretion in Tetrahymena. *Cell Biol Int Rep* 1: 69 – 73

Satir B, Schooley C, Satir P (1972) Membrane reorganization during secretion in Tetrahymena. *Nature* 235: 53 – 54

Schindelin J, Arganda-Carreras I, Frise E, Kaynig V, Longair M, Pietzsch T, Preibisch S, Rueden C, Saalfeld S, Schmid B *et al* (2012) Fiji: an open-source platform for biological-image analysis. *Nat Methods* 9: 676 – 682

Schneider CA, Rasband WS, Eliceiri KW (2012) NIH Image to ImageJ: 25 years of image analysis. *Nat Methods* 9: 671 – 675

Sheiner L, Demerly JL, Poulsen N, Beatty WL, Lucas O, Behnke MS, White MW, Striepen B (2011) A systematic screen to discover and analyze apicoplast proteins identifies a conserved and essential protein import factor. *PLoS Pathog* 7: e1002392

Sidik SM, Huet D, Ganesan SM, Huynh MH, Wang T, Nasamu AS, Thiru P, Saeij JP, Carruthers VB, Niles JC *et al* (2016) A genome-wide CRISPR screen in Toxoplasma identifies essential apicomplexan genes. *Cell* 166: 1423 – 1435.e12

Singer M, Simon K, Forné I, Meissner M (2022) A central protein complex essential for invasion in *Toxoplasma gondii*. *bioRxiv* https://doi.org/10.1101/2022.02.24.481622 [PREPRINT]

Singh S, Alam MM, Pal-Bhowmick I, Brzostowski JA, Chitnis CE (2010) Distinct external signals trigger sequential release of apical organelles during erythrocyte invasion by malaria parasites. *PLoS Pathog* 6: e1000746

Skorupa A, Urbach S, Vigy O, King MA, Chaumont-Dubel S, Prehn JH, Marin P (2013) Angiogenin induces modifications in the astrocyte secretome: relevance to amyotrophic lateral sclerosis. *J Proteomics* 91: 274 – 285

Sparvoli D, Lebrun M (2021) Unraveling the elusive rhoptry exocytic mechanism of Apicomplexa. *Trends Parasitol* 37: 622 – 637

Sparvoli D, Zoltner M, Cheng C-Y, Field MC, Turkewitz AP (2020) Diversification of CORVET tethers facilitates transport complexity in *Tetrahymena thermophila*. *J Cell Sci* 133: jcs238659

Suarez C, Lentini G, Ramaswamy R, Maynadier M, Aquilini E, Berry-Sterkers L, Cipriano M, Chen AL, Bradley P, Striepen B *et al* (2019) A lipid-binding protein mediates rhoptry discharge and invasion in *Plasmodium falciparum* and *Toxoplasma gondii* parasites. *Nat Commun* 10: 4041

Suss-Toby E, Zimmerberg J, Ward GE (1996) Toxoplasma invasion: the parasitophorous vacuole is formed from host cell plasma membrane and pinches off via a fission pore. *Proc Natl Acad Sci U S A* 93: 8413 – 8418

Tamura K, Stecher G, Kumar S (2021) MEGA11: molecular evolutionary genetics analysis version 11. *Mol Biol Evol* 38: 3022 – 3027

Thompson J, Fernandez-Reyes D, Sharling L, Moore SG, Eling WM, Kyes SA, Newbold CI, Kafatos FC, Janse CJ, Waters AP (2007) Plasmodium cysteine repeat modular proteins 1-4: Complex proteins with roles throughout the malaria parasite life cycle. *Cell Microbiol* 9: 1466 – 1480

Tsypin LM, Turkewitz AP (2017) The Co-regulation data harvester: automating gene annotation starting from a transcriptome database. *SoftwareX* 6: 165 – 171

Turkewitz AP, Madeddu L, Kelly RB (1991) Maturation of dense core granules in wild type and mutant *Tetrahymena thermophila*. *EMBO J* 10: 1979 – 1987

Tyanova S, Temu T, Sinitcyn P, Carlson A, Hein MY, Geiger T, Mann M, Cox J (2016) The perseus computational platform for comprehensive analysis of (prote)omics data. *Nat Methods* 13: 731 – 740

Yona S, Lin H-H, Siu WO, Gordon S, Stacey M (2008) Adhesion-GPCRs: emerging roles for novel receptors. *Trends Biochem Sci* 33: 491 – 500

Zimmermann L, Stephens A, Nam S-Z, Rau D, Kübler J, Lozajic M, Gabler F, Söding J, Lupas AN, Alva V (2018) A completely reimplemented MPI bioinformatics toolkit with a new HHpred server at its core. *J Mol Biol* 430: 2237 – 2243

License: This is an open access article under the terms of the Creative Commons Attribution License, which permits use, distribution and reproduction in any medium, provided the original work is properly cited.

Expanded View Figures

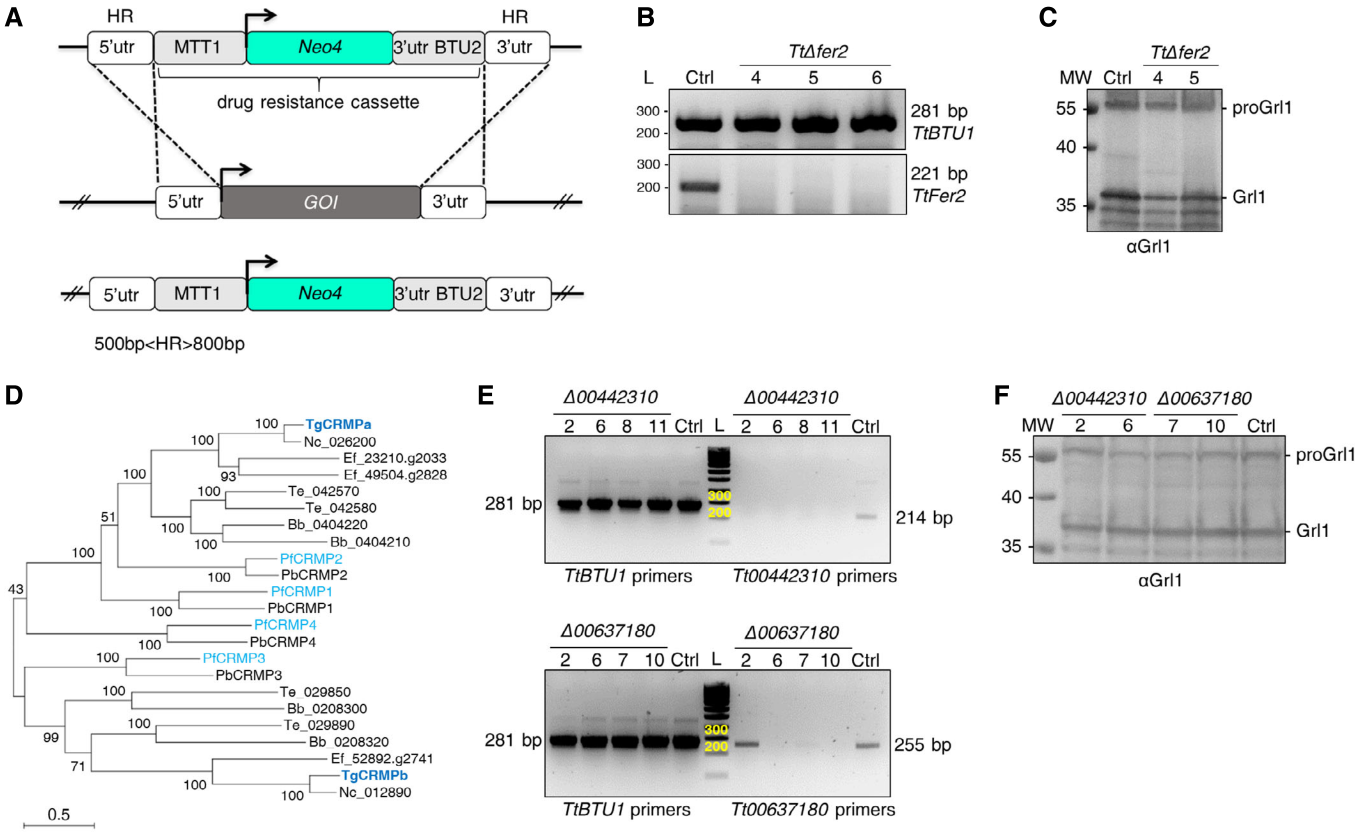

Figure EV1. *Tetrahymena* TtFer2, Tt00442310, and Tt00637180 are essential for mucocyst secretion (related to Fig 1).

A Strategy for the macronuclear knockout of *Tetrahymena thermophila* genes of interest (GOI). A linearized construct carrying fragments (HR) homologous to the 5′ and 3′-untranslated regions (UTR) of the GOI and flanking the drug resistance cassette were used to replace the GOI at the endogenous locus. The $CdCl_2$-inducible MTT1 promoter drives the expression of a paromomycin resistance gene (Neo4) used for selecting positive transformants.

B Disruption of the macronuclear copies of *TtFer2* (ferlin 2; TTHERM_00886960) was assessed by RT–PCR. cDNA from wild-type (Ctrl) and three clones of putative knockout cells (*Δfer2)* were PCR amplified with primers specific for *TtBTU1* (β-tubulin 1; upper panel) and *TtFer2* (lower panel). The 221 bp products corresponding to transcripts from *Fer2* are absent in the *Δfer2* clones, indicating that all the wild-type copies of *TtFer2* were efficiently replaced with the Neo4 cassette. All samples showed wild-type levels of *BTU1* transcripts. L: DNA ladder (bp). Primers are listed in Table EV1.

C Western blot of whole-cell lysates from wild-type (Ctrl) and *Δfer2* cells. In both, wild-type and mutant extracts, anti-Grl1 antibodies recognized the ~ 60 kDa precursor of the granule protein 1, proGrl1, and the processed form of Grl1, between 35 and 40 kDa, indicating non-significant defects in proteolytic maturation. MW: molecular weight standards.

D Phylogeny depicting the relationships between Apicomplexa CRMPs. The maximum-likelihood phylogenetic tree was obtained with the protein sequences of *CRMP* genes retrieved for the apicomplexans *Toxoplasma gondii* (TgCRMP), *Plasmodium falciparum* (PfCRMP), *Plasmodium berghei* (PbCRMP), *Neospora caninum* (Nc), *Eimeria falciformis* (Ef), *Theileria equi* (Te), and *Babesia bigemina* (Bb). *Toxoplasma* and *P. falciparum* CRMPs are highlighted in bold blue and light blue, respectively. Numbers at each node correspond to the bootstrap values. The scale bar represents the branch length.

E Disruption of the macronuclear copies of TTHERM_00442310 and TTHERM_00637180 was assessed by RT–PCR as in (B). Four clones for each putative knockout cell were tested. The 214 and 255 bp fragments corresponding to transcripts for TTHERM_00442310 and TTHERM_00637180, respectively, are absent in all *Δ00442310* clones, and nearly undetectable in clones 6, 7, and 10 for *Δ00637180*, indicating the achievement of full knockout. Clones 2 and 6 for *Δ00442310* and clones 7 and 10 for *Δ00637180* were selected for further analysis. All samples show wild-type levels of *BTU1* transcripts. L: DNA ladder (bp). Primers are listed in Table EV1.

F Western blot of whole-cell lysates from wild-type (Ctrl), *Δ00442310*, and *Δ00637180* cells. In both wild-type and mutant extracts, anti-Grl1 antibodies recognized processed Grl1 between 35 and 40 kDa and the precursor proGrl1 at ~ 60 kDa, indicating non-significant defects in proteolytic maturation. MW: molecular weight standards.

Source data are available online for this figure.

© 2022 The Authors

Figure EV2. TgCRMPa- and TgCRMPb-depleted tachyzoites have normal rhoptries, and show no defects in replication, stimulated egress, and attachment (related to Fig 2).

A Strategy for tagging genes of interest (GOI) in *Toxoplasma*. To generate C-terminal HA_3-fusions of TgCRMPa and TgCRMPb, a DNA fragment was amplified from a donor vector containing the HA_3 tag and the drug resistance cassette (CAT). Primers to amplify the DNA fragment were designed to contain ~ 30-bp-long stretches (HR) homologous to the GOI regions flanking the insertion site for the epitope tag. Upon CRISPR-cas9 cut (scissors), the PCR-amplified DNA fragment efficiently recombines into the targeted endogenous locus. The arrows indicate the binding sites of the primers used in (B).

B Integration of the HA_3 tag and CAT cassette at the C-terminus of TgCRMPa (upper panel) and TgCRMPb (lower panel) was tested by PCR. Genomic DNA from the untagged line and a clonal population for each of the putative HA_3-tagged lines was amplified with primers binding to the 3′ C-terminus and 3′UTR of each TgCRMP gene, in pairwise combination with primers binding the HA_3 and CAT sequences, respectively. The fragments corresponding to the HA_3 tag (5′) and the resistance cassette (3′) were correctly amplified in the putative tagged lines, indicating that they were efficiently integrated at the TgCRMPs loci. As expected, the wild-type fragment of each gene (wt) was detected only in the untagged line. L: DNA ladder (bp). Primers are listed in Table EV1.

C Strategies for the inducible depletion (iKD) of genes of interest (GOI) in *Toxoplasma*. The iKD lines for TgCRMPs were generated starting from the HA_3-tagged lines previously produced. In order to conditionally deplete the proteins, the endogenous promoter of each gene was replaced with an ATc-regulatable TetOSag4 promoter, preceded by the DHFR resistance cassette. The DNA fragment containing the cassette and the promoter was PCR amplified from a donor vector with primers containing ~ 30-bp-long homology regions (HR) specific for each gene and introduced upstream the starting codon via CRISPR-cas9 technology (scissors) and homologous recombination. The arrows indicate the binding sites of the primers used in (D) and Fig EV3G.

D Integration of the TetOSag4 promoter in TgCRMPa-HA_3 (upper panel) and TgCRMPb-HA_3 (lower panel) lines were tested by PCR. Integration of the DHFR resistance cassette was successfully PCR-amplified only for TgCRMPb-HA_3 (lower panel) line. Genomic DNA from untagged parasites and putative TgCRMPa_iKD and TgCRMPb_iKD clonal populations was amplified with primers binding to the 5′UTR and 5′ N-terminus of the GOI, flanking the DHFR-TetOSag4 insert, and used also in pairwise combination with primers binding the DHFR cassette and the TetOSag4 promoter, respectively. The fragments corresponding to the DHFR integration (5′) and TetOSag4 integration (3′) were detected exclusively in the putative iKD lines, while the wild-type fragment (wt) was amplified only in the untagged line. L: DNA ladder (bp). Primers are listed in Table EV1.

E Immunofluorescence images of untagged, TgCRMPa-HA_3, and TgCRMPb-HA_3 lines and TgCRMPs-depleted (iKD) intracellular tachyzoites. Parasites were stained with anti-HA and anti-ARM (ARO) Abs to visualize TgCRMPs and rhoptries, respectively. The nuclei (DNA) are stained with Hoechst. TgCRMPs pattern mirrors that of Fig 2B. Rhoptries show a wild-type appearance in the TgCRMPs-depleted parasites. Shown are single focal planes.

F Confocal immunofluorescence images of TgCRMPb-depleted (iKD) intracellular tachyzoites. Parasites were stained with anti-HA and with anti-GAMA and anti-PLP1 Abs to visualize TgCRMPb and micronemes, respectively. The nuclei (DNA) are stained with Hoechst. Shown are single focal planes.

G Extent of co-localization between TgCRMPb-HA_3 (light red) and microneme proteins AMA1, MIC2, GAMA, and PLP1 shown in (F) and Fig 2D. Untagged parasites were analyzed in parallel to estimate the background noise (light gray), and the extent of overlap between the microneme proteins AMA1 and MIC2 (dark gray). Pearson's correlation coefficient was measured using the Fiji-JACoP plugin. Values are expressed as mean ± SD; *n*, number of parasites analyzed.

H Replication measured for TgCRMPa- and TgCRMPb-depleted parasites. The percentage of vacuoles with 2, 4, 8, 16, and 32 parasites was calculated for control (Ctrl), and TgCRMPa_iKD and TgCRMPb_iKD lines, in the absence of ATc and upon 48 and 24 h ATc treatment, respectively. Both iKD mutants (+ATc) are capable of efficient replication. Data are reported as mean ± SD ($n = 2$ biological replicates, each with three technical replicates).

I Stimulated egress was quantified for TgCRMPa- and TgCRMPb-depleted parasites. Infected cells with intact vacuoles were treated with A23187 to induce parasite egress, measured as a number of burst vacuoles over the total number of vacuoles. Egress was tested for control (Ctrl), TgCRMPa_iKD and TgCRMPb_iKD lines, in the absence of ATc and upon 48 and 24 h ATc treatment, respectively. Values are reported as mean ± SD ($n = 3$ biological replicates, each with three technical replicates). The biological replicates are represented by different symbols. *P*-values are non-significant for all datasets (two-tailed *t*-test).

J Attachment measured for TgCRMPa- and TgCRMPb-depleted parasites. The number of parasites attached to the host cell was counted for control (Ctrl), TgCRMPa_iKD and TgCRMPb_iKD lines, in the absence of ATc and upon 48 and 24 h ATc treatment, respectively. BAPTA treatment was used as a control since it prevents attachment. TgCRMPa- and TgCRMPb-depleted parasites were able to attach to host cells. Values are reported as in (I; $n = 3$ biological replicates, each with three technical replicates). The biological replicates are represented by different symbols. *P*-values were measured by a two-tailed *t*-test.

Source data are available online for this figure.

© 2022 The Authors

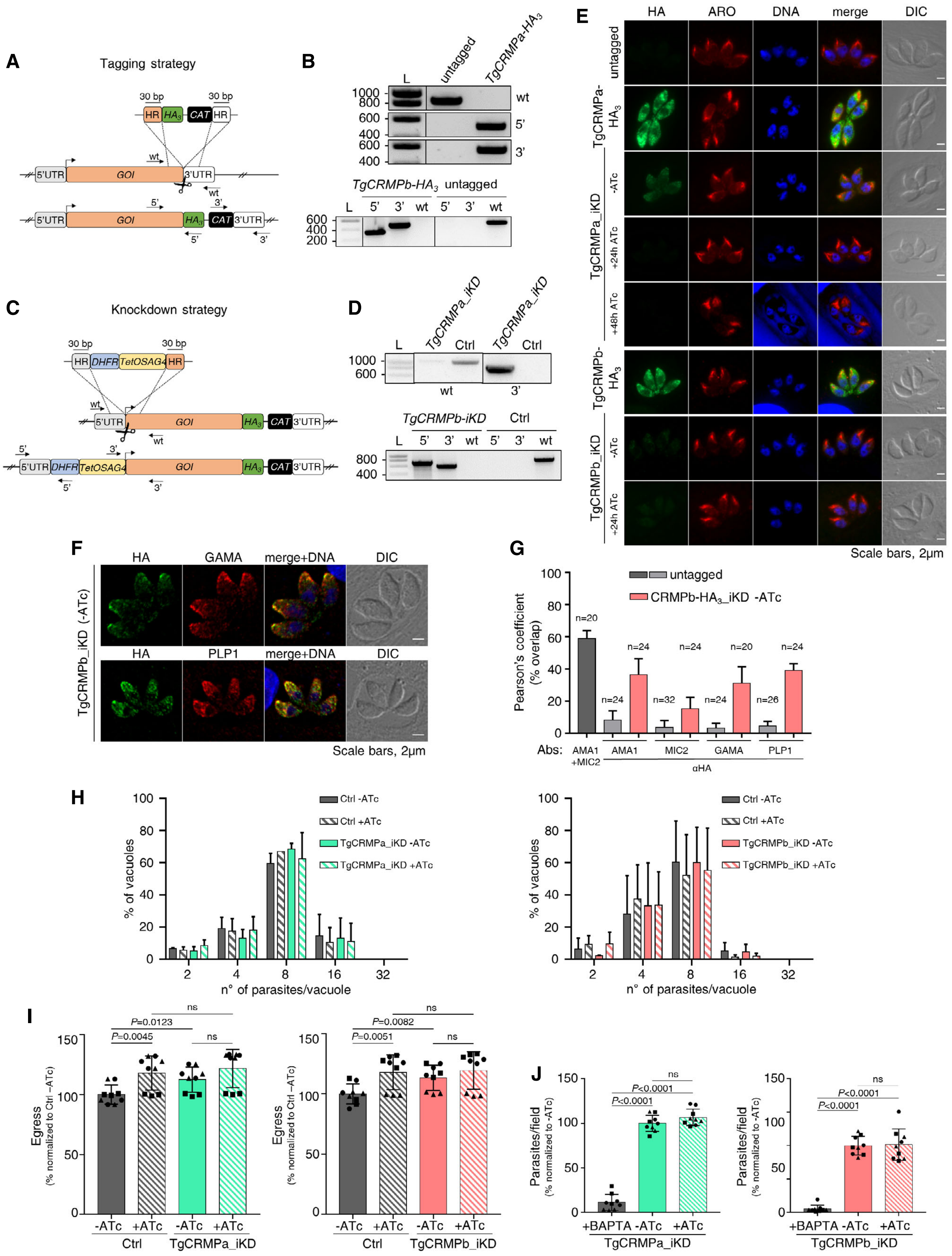
A
Tagging strategy
B
untagged
TgCRMPa-HA3
TgCRMPb-HA3 untagged
C
Knockdown strategy
D
TgCRMPa_iKD
Ctrl
TgCRMPb-iKD
E
HA ARO DNA merge DIC
untagged
TgCRMPa-HA3
TgCRMPa_iKD
-ATc
+24h ATc
+48h ATc
TgCRMPb-HA3
TgCRMPb_iKD
Scale bars, 2µm
F
HA GAMA merge+DNA DIC
HA PLP1 merge+DNA DIC
TgCRMPb_iKD (-ATc)
Scale bars, 2µm
G
untagged
CRMPb-HA3_iKD -ATc
Pearson's coefficient (% overlap)
Abs: AMA1 +MIC2 AMA1 MIC2 GAMA PLP1
αHA
H
Ctrl -ATc
Ctrl +ATc
TgCRMPa_iKD -ATc
TgCRMPa_iKD +ATc
TgCRMPb_iKD -ATc
TgCRMPb_iKD +ATc
% of vacuoles
n° of parasites/vacuole
I
Egress (% normalized to Ctrl -ATc)
Ctrl TgCRMPa_iKD
Ctrl TgCRMPb_iKD
J
Parasites/field (% normalized to -ATc)
+BAPTA -ATc +ATc
TgCRMPa_iKD
TgCRMPb_iKD

Figure EV2.

Figure EV3. Tg277910-depleted tachyzoites with a disrupted lytic cycle show no defects in microneme staining, replication, stimulated egress, and attachment (related to Fig 3).

A Coomassie Blue staining of eluted proteins (1, 3, 5) immunoprecipitated (IP) with anti-HA beads, and protein fractions of the corresponding clear lysates (CL; 2, 4, 6) prior to beads incubation, from TgCRMPa-HA_3, TgCRMPb-HA_3, and untagged lines. The TgCRMP protein used as bait in each IP lane is indicated by the asterisk. Samples in lanes 1, 3, and 5 were analyzed by mass spectrometry. MW: molecular weight standards.

B Marker-free strategy for $FLAG_3$ tagging of TgCRMPa. To generate a C-terminal $FLAG_3$ fusion of TgCRMPa, a gBlock containing the $FLAG_3$ tag flanked by ~ 30-bp-long TgCRMPa homology regions (HR) was amplified and integrated into the TgCRMPa endogenous locus via CRISPR-cas9 technology (scissors). The $FLAG_3$-tagged TgCRMPa was generated also in the TgCRMPb-HA_3 line. The arrows indicate the binding sites of the primers used in (C).

C Integration of the $FLAG_3$ tag was tested by PCR in putative TgCRMPa-$FLAG_3$ and TgCRMPa-$FLAG_3$ + TgCRMPb-HA_3 lines. The addition of the tag at the C-terminus of the TgCRMPa gene corresponds to the insertion of an additional 74 bp to the wild-type sequence. A higher band was observed in the putative tagged lines compared to the untagged ones. DNA ladder (L) is shown on the left of each panel. Primers are listed in Table EV1.

D Eluates from Fig 3B and 1/20 of the clear lysates (before beads incubation) were also immunoblotted with anti-ROP5 antibodies to confirm the specificity of the immunoprecipitation experiments. The red arrowhead indicates TgROP5 protein, and the asterisk indicates unspecific bands detected in the eluates, likely corresponding to the light chain of the beads-conjugated antibody. MW: molecular weight standards.

E Strategy based on the pLIC system (Huynh & Carruthers, 2009) for tagging Tg277910 with triple HA. The arrows indicate the binding sites of the primers used in (F).

F Integration of the HA_3 tag and CAT cassette at the C-terminus of TGGT1_277910 was tested by PCR. Genomic DNA from an untagged line and a clonal population for the putative HA_3-tagged line were amplified with primers binding to the 3′ C-terminus of TGGT1_277910 and HA_3 sequence. The HA_3 tag (5′) was correctly amplified indicating that it was efficiently integrated at the TGGT1_277910 locus. L: DNA ladder (bp). Primers are listed in Table EV1.

G Integration of the DHFR cassette followed by the TetOSag4 promoter in TGGT1_277910 line was tested by PCR as in Fig EV2C and D. Genomic DNA from untagged parasites and putative Tg277910_iKD clonal population was amplified with primers binding the gene's 5′UTR and 5′ N-terminus, flanking the DHFR-TetOSag4 insert, and used also in pairwise combination with primers binding the DHFR cassette and the Sag4 promoter, respectively. The wild-type fragment (wt) was amplified only in the control line (Ctrl), while the fragments corresponding to the DHFR integration (5′) and TetOSag4 integration (3′) were detected exclusively in the putative iKD line. A low-abundant unspecific band of similar size to the 3′ fragments was observed in the untagged line. L: DNA ladder (bp). Primers are listed in Table EV1.

H Whole-cell lysates were collected from Tg277910-HA_3 parasites (HA_3) and from the line generated for the inducible knockdown (iKD) treated with ATc for 24, 48, and 72 h and untreated. The samples were immunoblotted with anti-HA Abs (upper panel) to visualize Tg277910 protein under all mentioned conditions. TgROP5 was used as a loading control (lower panel). A band corresponding to the predicted size for Tg277910 (~ 138 kDa) was detected in the untreated samples (−) and decreased overtime in the ATc-treated ones (+) to completely disappear upon 72 h of ATc treatment. Protein molecular weight standards (MW) are shown on the left of each panel.

I Immunofluorescence images of untagged and Tg277910-HA_3- and Tg277910-depleted (iKD) intracellular tachyzoites. Parasites were stained with anti-HA and anti-AMA1 Abs to label Tg277910 and micronemes, respectively. The nuclei (DNA) are stained with Hoechst. Tg277910-HA_3 pattern mirrors that of Fig 3D. Micronemes show a wild-type appearance in the Tg277910-depleted parasites. Shown are single focal planes.

J Quantification of plaques for Tg277910-depleted parasites. Lysis plaque areas were measured for untreated and 72 h ATc-treated control and iKD lines. Values are reported as mean ± SD ($n = 3$ biological replicates, each with three technical replicates). The biological replicates are represented by different symbols.

K–M Quantification of replication (K), stimulated egress (L), and attachment (M) for control (Ctrl) and Tg277910-depleted (iKD) lines were performed as in Fig EV2H–J, respectively, with 72 h ATc-treated and untreated parasites. Tg277910-depleted parasites replicate, egress, and attach normally. Values are reported as in (J; replication and egress: $n = 2$ biological replicates, each with three technical replicates; attachment: $n = 3$ biological replicates, each with three technical replicates). The biological replicates are represented by different symbols.

Data information: *P*-values in (J and M) were measured by a two-tailed *t*-test.
Source data are available online for this figure.

 © 2022 The Authors

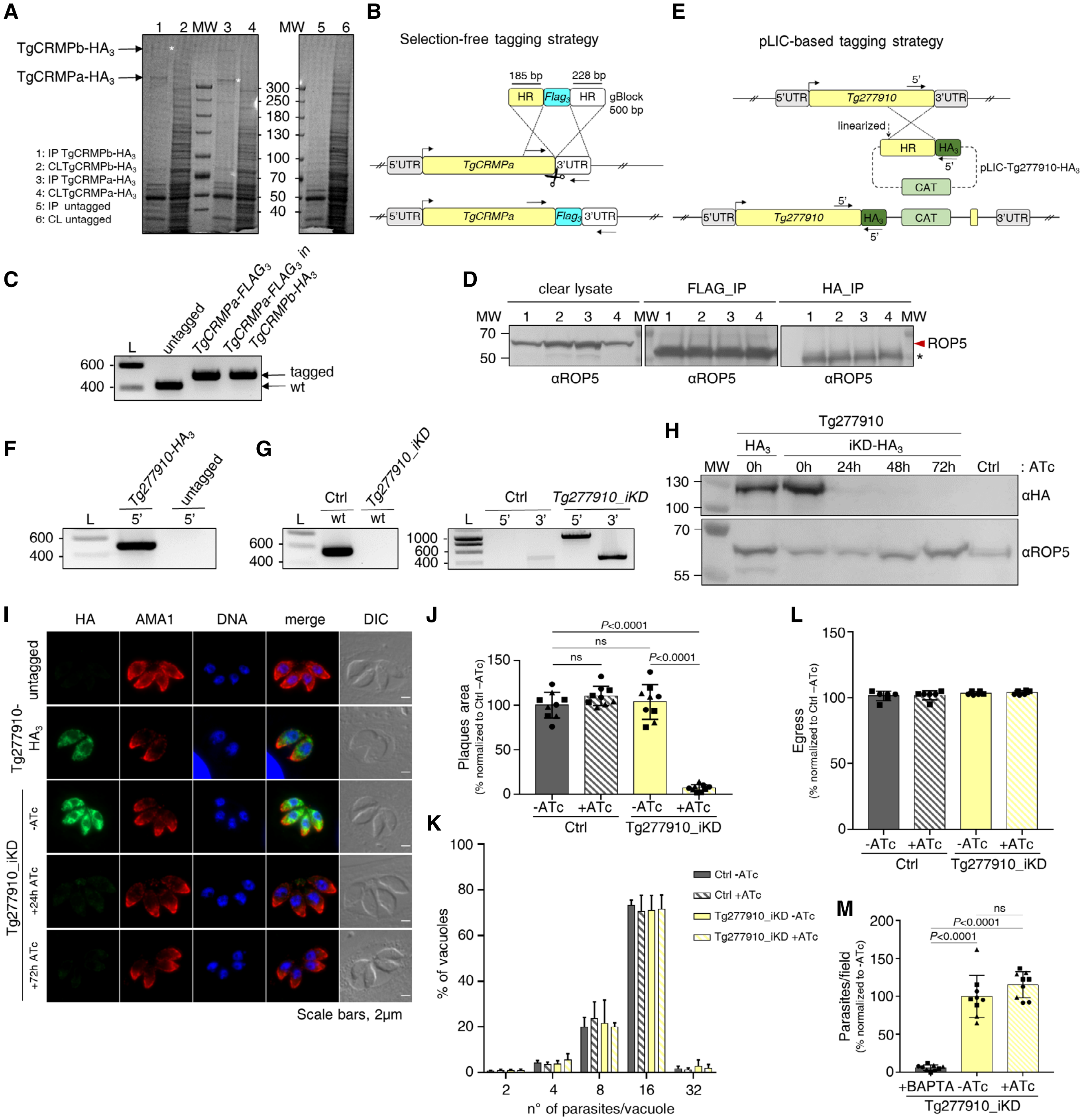
A
TgCRMPb-HA3
TgCRMPa-HA3
1 2 MW 3 4 MW 5 6
1: IP TgCRMPb-HA3
2: CLTgCRMPb-HA3
3: IP TgCRMPa-HA3
4: CLTgCRMPa-HA3
5: IP untagged
6: CL untagged
B
Selection-free tagging strategy
185 bp 228 bp
HR Flag3 HR gBlock 500 bp
5'UTR TgCRMPa 3'UTR
E
pLIC-based tagging strategy
5'UTR Tg277910 3'UTR
linearized
pLIC-Tg277910-HA3
CAT
C
untagged
TgCRMPa-FLAG3
TgCRMPa-FLAG3 in TgCRMPb-HA3
tagged
wt
D
clear lysate
FLAG_IP
HA_IP
ROP5
αROP5
F
Tg277910-HA3
untagged
G
Tg277910_iKD
Ctrl
H
Tg277910
HA3
iKD-HA3
0h 24h 48h 72h Ctrl : ATc
αHA
αROP5
I
HA AMA1 DNA merge DIC
untagged
Tg277910-HA3
Tg277910_iKD
-ATc
+24h ATc
+72h ATc
Scale bars, 2μm
J
Plaques area (% normalized to Ctrl –ATc)
-ATc +ATc -ATc +ATc
Ctrl Tg277910_iKD
P<0.0001
ns
K
% of vacuoles
n° of parasites/vacuole
Ctrl -ATc
Ctrl +ATc
Tg277910_iKD -ATc
Tg277910_iKD +ATc
L
Egress (% normalized to Ctrl –ATc)
M
Parasites/field (% normalized to -ATc)
+BAPTA -ATc +ATc

Figure EV3.

Figure EV4. TgCRMPa and TgCRMPb accumulate at the tip of the extruded conoid (related to Fig 5).

A Quantification of the dot pattern for TgCRMPa-HA_3 in TgCRMPa-depleted (iKD) tachyzoites. TgCRMPa accumulation at the apical tip of extracellular parasites was measured upon incubation with host cell monolayers for 2 min to stimulate natural conoid extrusion. CRMPa signal at the apical dot disappeared after 48 h ATc treatment, indicating that the association with the tip of the extruded conoid was specific. No significant apical signal was detected for the control line (Ctrl), as in Fig 5B. Numbers are expressed as a percentage of parasites showing (dot) or lacking (no dot) the tip accumulation of TgCRMPa. The number of parasites (*n*) analyzed for each line is reported on the column tops.

B Strategy for the inducible depletion (iKD) of TgCRMPa-$FLAG_3$. The iKD lines were generated starting from the $FLAG_3$-tagged lines previously produced. In order to conditionally deplete the protein, the endogenous promoter of the TgCRMPa-$FLAG_3$ gene was replaced with an ATc-regulableTetOSag4 promoter, preceded by the DHFR resistance cassette. The DNA fragment containing the cassette and promoter was PCR-amplified from a donor vector with primers containing ~ 30-bp-long homology regions (HR) specific for *TgCRMPa* gene, and introduced upstream to the starting codon via CRISPR-cas9 technology (scissors) and homologous recombination. The arrows indicate the binding sites of the primers used in (C).

C Integration of the DHFR cassette followed by the TetOSag4 promoter in the putative TgCRMPa-$FLAG_3$ and TgCRMPa-$FLAG_3$ + TgCRMPb-$FLAG_3$ iKD lines was tested by PCR as in Fig EV2C and D (upper panel). The fragments corresponding to the DHFR integration (5′) and TetOSag4 integration (3′) were detected exclusively in the putative iKD lines, while the wild-type fragment (wt) was amplified only in the control line (Ctrl). L: DNA ladder (bp). Primers are listed in Table EV1.

D Whole-cell lysates from untagged and TgCRMPa-$FLAG_3$_iKD and TgCRMPa-$FLAG_3$_iKD + TgCRMPb-HA_3 lines were immunoblotted with anti-FLAG Abs to visualize tagged CRMPa in ATc-treated and untreated samples. CRMPa disappeared upon 48 h ATc incubation in both lines. A ~ 300 kDa unspecific cross-reactive band was observed in all samples. MW: molecular weight standards.

E Auxin-degron strategy used for generating TgCRMPa-miniAID-HA_3 strain. The integration of the tag and drug resistance cassette into the *TgCRMPa* locus is ensured by ~ 30-bp-long homology regions (HR) upon CRISPR-Cas9 activity (scissors). The arrows indicate the binding sites of the primers used in (F).

F Integration of the miniAID-HA_3 and HXGPRT cassette at the *TgCRMPa* locus in the Tir-1 line was tested by PCR as in Fig EV2A and B (upper panel). The fragments corresponding to the miniAID-HA_3 (5′) and HXGPRT cassette (3′) integration were detected exclusively in the putative iKD line, while the wild-type fragment (wt) was amplified only in the untagged line. A ~ 4,000 bp fragment corresponding to the miniAID-HA_3 + HXGPRT cassette, and amplified with primers binding the wild-type sequence, was detected in the iKD line. L: DNA ladder (bp). Primers are listed in Table EV1.

G Marker-free strategy used for generating HA_3-miniAID-TgCRMPa strain. The integration of the tag at the N-terminus between residues Val69 and Leu70 (before the MAR/Kringle domain; Fig 5E) into the *TgCRMPa* locus is ensured by 207- and 265-bp-long homology regions (HR) flanking the tag in the synthetic gBlock, upon CRISPR-Cas9 activity (scissors). The arrows indicate the binding sites of the primers used in (H).

H Integration of the miniAID-HA_3 at the N-terminus of the *TgCRMPa* locus in the Tir-1 parental line was tested by PCR. The fragments corresponding to the HA_3 (5′) and the HA_3-miniAID (3′) integration were detected exclusively in the putative iKD line; the wild-type fragment (wt) was amplified in the untagged line (~ 1,556 bp) and iKD line (~ 1,026 bp, tag minus introns). L: DNA ladder (bp). Primers are listed in Table EV1.

I Immunofluorescence images of untagged and N-terminal and C-terminal miniAID-HA_3-TgCRMPa (iKD) intracellular tachyzoites. Parasites treated 24 h with IAA, as well as untreated (−IAA), were stained with anti-HA Abs. The nuclei (DNA) are stained with Hoechst. Shown are single focal planes.

J Representative images of lytic plaques formation in HFF monolayers infected with IAA-treated and untreated Tir-1 control and N-terminal and C-terminal miniAID-HA_3-TgCRMPa (iKD) lines.

K Immunofluorescence images of extracellular N-terminal and C-terminal miniAID-HA_3-TgCRMPa tachyzoites. Parasites were incubated with ionophore A23187 to induce artificial conoid extrusion, and stained with anti-HA Abs. TgCRMPa localization at the tip of the extruded conoid (arrow) is visible only in the C-terminally miniAID-HA_3-tagged TgCRMPa (lower panel). DNA is labeled by Hoechst. Single focal planes are shown. DIC: differential interference contrast.

L Quantification of the dot pattern for HA_3-miniAID-TgCRMPa (N-term) and TgCRMPa-miniAID-HA_3 (C-term) tachyzoites. TgCRMPa accumulation at the apical tip of extracellular parasites was measured upon incubation with ionophore A23187 to induce artificial conoid extrusion. Parasites were fixed and stained with anti-HA Abs and with (+ triton) or without (− triton) permeabilization. CRMPa signal at the apical dot is absent in non-permeabilized parasites, and it is robustly detected only in permeabilized parasites expressing C-terminally miniAID-HA_3-tagged TgCRMPa. No significant apical signal was detected for the control (untagged) or the N-terminally miniAID-HA_3-tagged TgCRMPa lines. Numbers are expressed as a percentage of parasites showing (dot) or lacking (no dot) the tip accumulation of TgCRMPa. The number of parasites (*n*) analyzed for each line is reported on the column tops.

M Marker-free strategy used for generating HA_3-TgCRMPa strain. The integration of the tag at the N-terminus between residues Thr600 and Asn601 (after the MAR/Kringle domain; Fig 5H) into the *TgCRMPa* locus is ensured by 200-bp-long homology regions (HR), flanking the tag in the synthetic gBlock upon CRISPR-Cas9 activity (scissors). The arrows indicate the binding sites of the primers used in (N).

N Integration of the triple HA at the N-terminus of the *TgCRMPa* locus was tested by PCR. The fragments corresponding to the 5′ and 3′ integration were detected exclusively in the putative HA_3-tagged line; and the wild-type fragment (wt) was amplified in the untagged line (~ 1,424 bp) and tagged line (~ 1,550 bp, containing linker+HA_3). L: DNA ladder (bp). Primers are listed in Table EV1.

O Immunofluorescence image of intracellular HA_3-TgCRMPa tachyzoites. Parasites were stained with anti-HA Abs and DNA is labeled by Hoechst. Single focal planes are shown. DIC, differential interference contrast.

Source data are available online for this figure.

 © 2022 The Authors

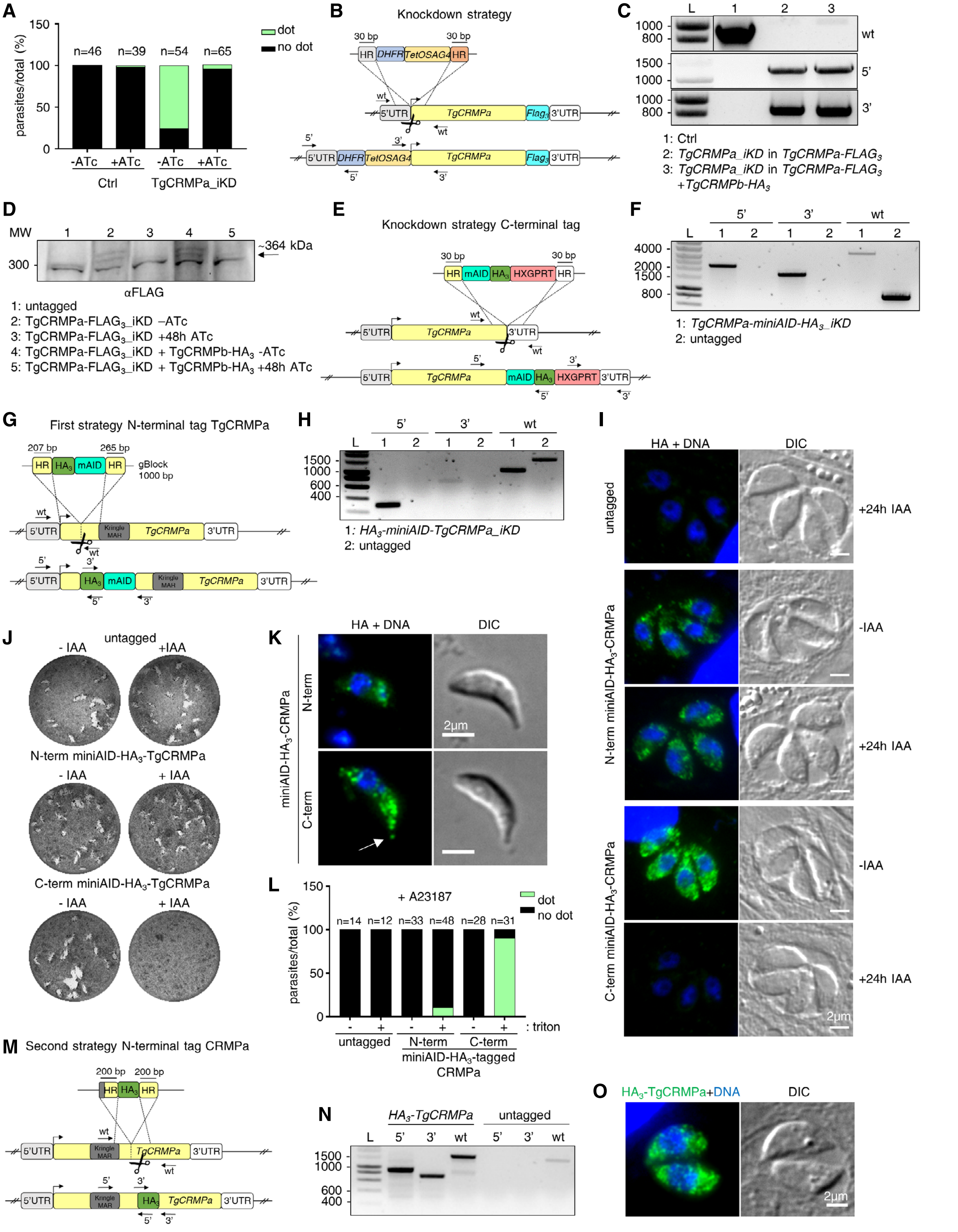
A
dot
no dot
parasites/total (%)
n=46 n=39 n=54 n=65
-ATc +ATc -ATc +ATc
Ctrl TgCRMPa_iKD
B Knockdown strategy
C
1: Ctrl
2: TgCRMPa_iKD in TgCRMPa-FLAG3
3: TgCRMPa_iKD in TgCRMPa-FLAG3 +TgCRMPb-HA3
D
~364 kDa
αFLAG
1: untagged
2: TgCRMPa-FLAG3_iKD –ATc
3: TgCRMPa-FLAG3_iKD +48h ATc
4: TgCRMPa-FLAG3_iKD + TgCRMPb-HA3 -ATc
5: TgCRMPa-FLAG3_iKD + TgCRMPb-HA3 +48h ATc
E Knockdown strategy C-terminal tag
F
1: TgCRMPa-miniAID-HA3_iKD
2: untagged
G First strategy N-terminal tag TgCRMPa
gBlock 1000 bp
H
1: HA3-miniAID-TgCRMPa_iKD
2: untagged
I
HA + DNA
DIC
untagged
N-term miniAID-HA3-CRMPa
C-term miniAID-HA3-CRMPa
+24h IAA
-IAA
J
untagged
N-term miniAID-HA3-TgCRMPa
C-term miniAID-HA3-TgCRMPa
- IAA
+ IAA
K
miniAID-HA3-CRMPa
N-term
C-term
L
+ A23187
n=14 n=12 n=33 n=48 n=28 n=31
: triton
untagged
N-term C-term
miniAID-HA3-tagged CRMPa
M Second strategy N-terminal tag CRMPa
N
HA3-TgCRMPa
untagged
O
HA3-TgCRMPa+DNA
DIC

Figure EV4.

Figure EV5. CRMPs and Nd6 co-localize at the exocytic site in extracellular *Toxoplasma gondii* (related to Fig 6).

A Strategy for TY_2 tagging of TgNd6 in TgCRMPa-HA_3 and TgCRMPb-HA_3 lines. To generate a C-terminal TY_2-fusion of TgNd6, a DNA fragment was amplified from a donor vector containing the TY_2 tag and the drug resistance cassette (DHFR). Primers to amplify the DNA fragment were designed to contain 30-bp-long stretches (HR) homologous to TgND6 regions flanking the insertion site for the epitope tag. Upon CRISPR-cas9 cut (scissors), the PCR-amplified DNA fragment efficiently recombines into the targeted endogenous locus. The arrows indicate the binding sites of the primers used in (B).

B Integration of the TY_2 tag and DHFR cassette at the C-terminus of *TgND6* locus was tested by PCR. Genomic DNAs from an untagged line and clonal populations for TgNd6-TY_2 + TgCRMPa-HA_3 and TgNd6-TY_2 + TgCRMPb-HA_3 lines were amplified with primers binding to the 3′ C-terminus and 3′UTR of *TgNd6*, and also in pairwise combination with primers binding the TY_2 and DHFR sequences, respectively. The fragments corresponding to the TY_2 tag (5′) and the resistance cassette (3′) were correctly amplified in the putative tagged lines, indicating that they were efficiently integrated at the *TgNd6* locus. As expected, the wild-type fragment for *TgNd6* (wt) was detected only in the untagged line. L: DNA ladder (bp). Primers are listed in Table EV1.

C Whole-cell lysates from untagged and TgNd6-TY_2 + TgCRMPa-HA_3 and TgNd6-TY_2 + TgCRMPb-HA_3 parasites were immunoblotted with anti-TY Abs to detect tagged Nd6. A band around the expected size (~ 195 kDa) for TgNd6-TY_2 was observed exclusively for the tagged lines. MW: molecular weight standards.

D Immunofluorescence images of intracellular (upper and middle panels) and extracellular (lower panel) tachyzoites from untagged and TgCRMPb-HA_3 + TgNd6-TY_2 lines. Extracellular parasites were incubated with host cell monolayers for 2 min prior to fixation. Parasites were stained with anti-HA and anti-TY Abs to label CRMPb and Nd6, respectively. Nd6, but not CRMPb, accumulates at the tachyzoite apex in intracellular parasites (arrowheads), while both proteins localize at the tip of the extruded conoid in extracellular parasites (arrows). DNA is labeled by Hoechst. Single focal planes are shown. DIC, differential interference contrast.

E Integration of the HA_3 tag and CAT cassette at the C-terminus of *TgCRMPa* and *TgCRMPb* genes in TgNd9_iKD line was tested by PCR as in Fig EV2A and B. The fragments corresponding to the HA_3 tag (5′) and the resistance cassette (3′) were correctly amplified in the putative tagged lines, indicating that they were efficiently integrated at the TgCRMPs loci. As expected, the wild-type fragment of each gene (wt) was detected only in the untagged line. L: DNA ladder. Primers are listed in Table EV1.

F Depletion of *TgNd9* transcripts was assessed by RT–PCR for the experiment shown in Fig 6D. Total RNAs from TgCRMPa-HA_3 and TgCRMPb-HA_3 expressed in TgNd9_iKD (minus epitope tag) parasites and parental line were subjected to reverse transcription and PCR amplified with primers binding *TgNd9* transcripts. *TgGAPDH* was used as housekeeping gene. *TgNd9* transcripts strongly decreased upon 72 h ATc treatment (+ATc). L: DNA ladder (L). Primers are listed in Table EV1.

G Depletion of TgNd9 proteins in the lines used for the experiment in Fig 6D was also assessed by quantifying the defect in the invasion of ATc-treated TgNd9_iKD parasites expressing TgCRMPa-HA_3 and TgCRMPb-HA_3 versus untreated. The values are reported as percentages of the number of invading/intracellular and extracellular parasites over the total number of parasites. The number of fields (f) analyzed for each line is reported on the column tops.

H Immunofluorescence images of intracellular and extracellular tachyzoites from TgCRMPb-HA_3 + TgCRMPa-TY_2 line. Extracellular parasites were incubated with host cell monolayers for 2 min prior to fixation. Parasites were stained with anti-HA and anti-TY Abs to label CRMPb and CRMPa, respectively. Both proteins localize at the tip of the extruded conoid in extracellular parasites (arrows) and show partial overlap within the parasite cytosol. An untagged line was used to estimate the background noise. DNA is labeled by Hoechst. Single focal planes are shown. DIC, differential interference contrast.

I Integration of the TY_2 tag and DHFR cassette at the C-terminus of the *TgCRMPa* locus in TgCRMPb-HA_3 line was tested by PCR as described in (B) for TgNd6-TY_2. The fragments corresponding to the TY_2 tag (5′) and the resistance cassette (3′) were correctly amplified in the putative tagged line, indicating that they were efficiently integrated at the *TgCRMPa* locus. As expected, the wild-type fragment for *TgCRMPa* (wt) was detected only in the untagged line. L: DNA ladder (bp). Primers are listed in Table EV1.

J Whole-cell lysates from TgCRMPb-HA_3 and TgCRMPb-HA_3 + TgCRMPa-TY_2 parasites were immunoblotted with anti-TY Abs to detect tagged CRMPa. A band around the expected size (~ 348 kDa) for TgCRMPa-TY_2 together with the processed form were observed exclusively for the tagged line. MW: molecular weight standards.

Source data are available online for this figure.

© 2022 The Authors

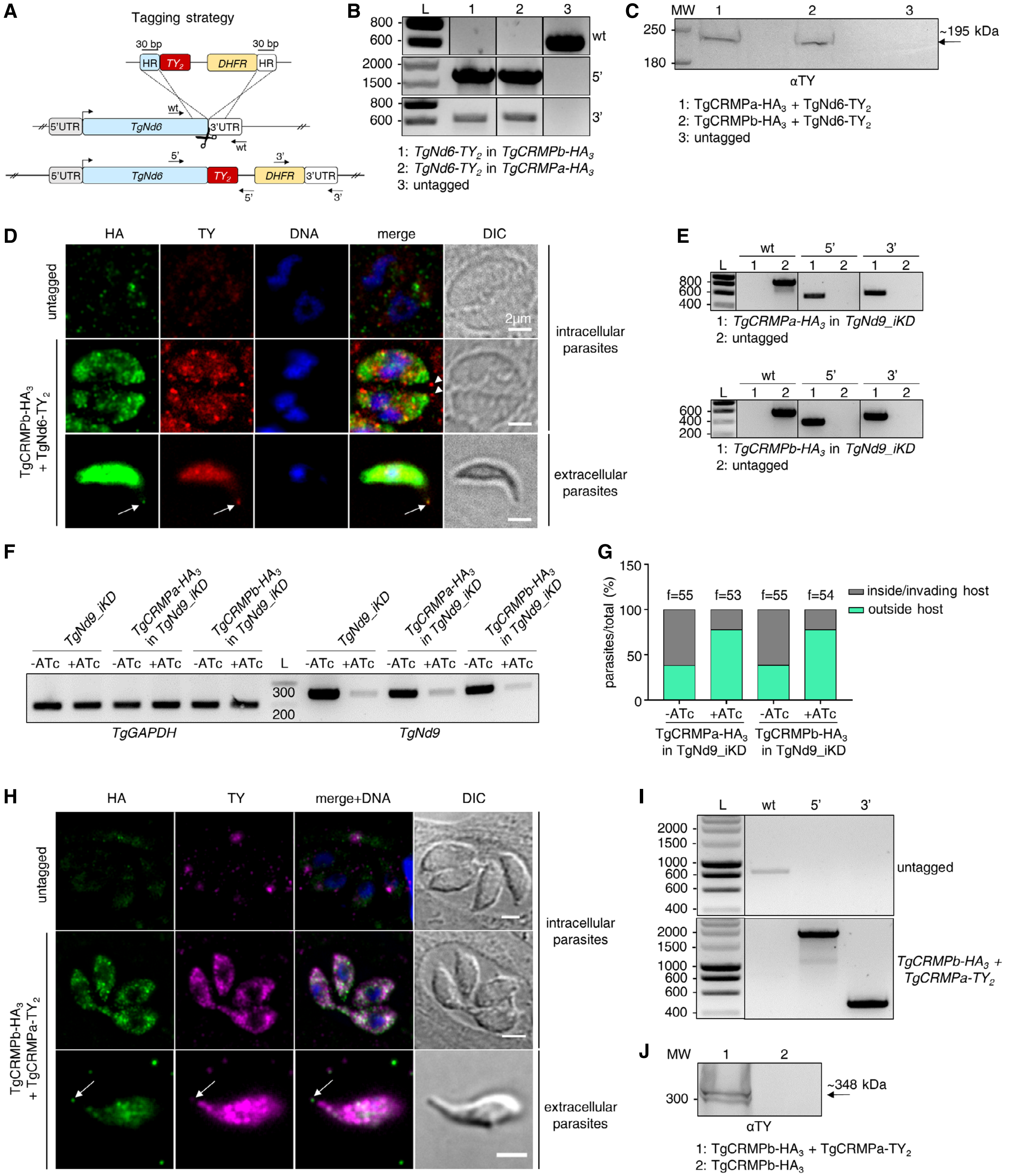
A
Tagging strategy
30 bp
HR
TY2
DHFR
HR
5'UTR
TgNd6
3'UTR
wt
5'
3'
B
L 1 2 3
800
600
2000
1500
wt
5'
3'
1: TgNd6-TY2 in TgCRMPb-HA3
2: TgNd6-TY2 in TgCRMPa-HA3
3: untagged
C
MW 1 2 3
250
180
~195 kDa
αTY
1: TgCRMPa-HA3 + TgNd6-TY2
2: TgCRMPb-HA3 + TgNd6-TY2
3: untagged
D
HA TY DNA merge DIC
untagged
TgCRMPb-HA3 + TgNd6-TY2
2µm
intracellular parasites
extracellular parasites
E
wt 5' 3'
L 1 2 1 2 1 2
800
600
400
1: TgCRMPa-HA3 in TgNd9_iKD
2: untagged
600
400
200
1: TgCRMPb-HA3 in TgNd9_iKD
2: untagged
F
TgNd9_iKD
TgCRMPa-HA3 in TgNd9_iKD
TgCRMPb-HA3 in TgNd9_iKD
-ATc +ATc L
300
200
TgGAPDH
TgNd9
G
parasites/total (%)
150
100
50
0
f=55 f=53 f=55 f=54
inside/invading host
outside host
-ATc +ATc -ATc +ATc
TgCRMPa-HA3 in TgNd9_iKD
TgCRMPb-HA3 in TgNd9_iKD
H
HA TY merge+DNA DIC
untagged
TgCRMPb-HA3 + TgCRMPa-TY2
intracellular parasites
extracellular parasites
I
L wt 5' 3'
2000
1500
1000
600
400
untagged
TgCRMPb-HA3 + TgCRMPa-TY2
J
MW 1 2
300
~348 kDa
αTY
1: TgCRMPb-HA3 + TgCRMPa-TY2
2: TgCRMPb-HA3

Figure EV5.

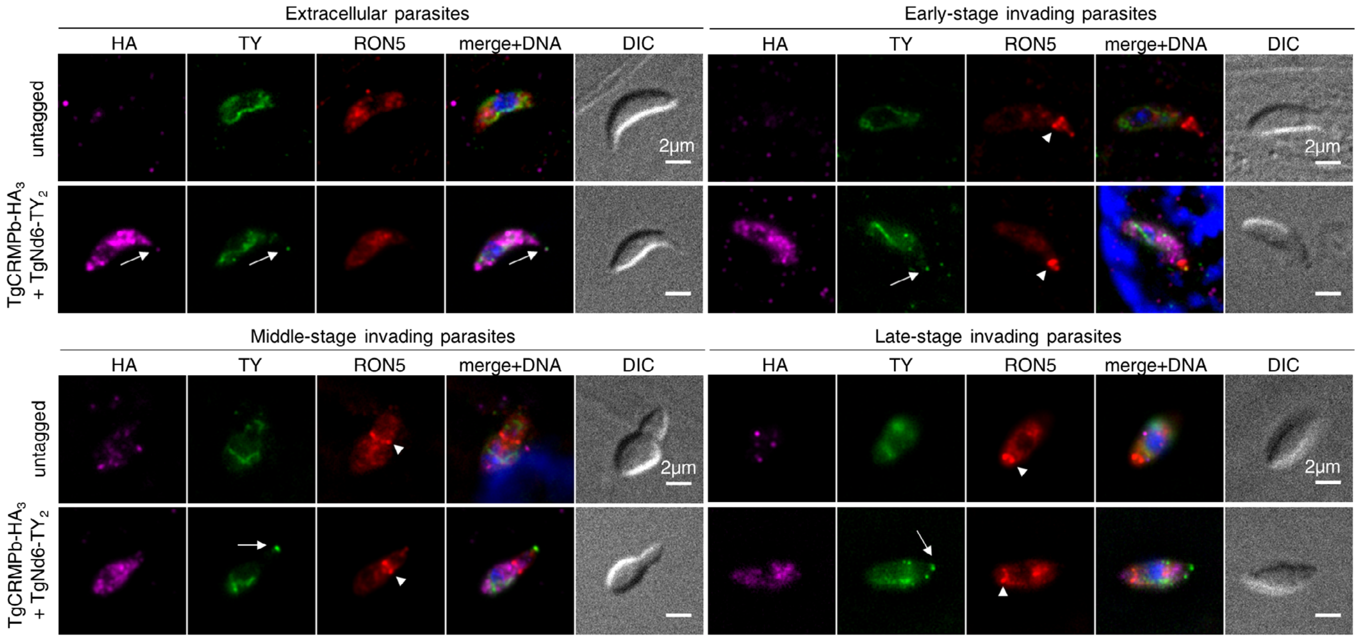

Figure EV6. CRMP and Nd complexes show different dynamics at the exocytic site in *Toxoplasma gondii* (related to Fig 6).

Immunofluorescence images of extracellular parasites and parasites in early, middle, and late stages of host cell invasion. Parasites co-expressing TgCRMPb-HA_3 with TgNd6-TY_2 were incubated with host cell monolayers and stained as in Fig 6E. Untagged parasites were treated in parallel. In contrast with TgNd6 (arrow), the apical accumulation of TgCRMPb observed in extracellular parasites disappears upon entering the host and remains undetected for the entire process. The moving junction is indicated by the arrowhead. Non-specific anti-TY labeling of mitochondria was detected for both untagged and tagged lines. DIC, differential interference contrast. Single focal planes are shown.

 © 2022 The Authors
