# Supplementary material for: An apical membrane complex for triggering rhoptry exocytosis and invasion in Toxoplasma
Source: EMBO J. 2022 Oct 17;41(22):e111158. doi: 10.15252/embj.2022111158 (PMC9670195; doi:10.15252/embj.2022111158)
Supplement: Supplementary file 12 [file EMBJ-41-e111158-s009.pdf]

THE EMBO JOURNAL

# An apical membrane complex for triggering rhoptry exocytosis and invasion in Toxoplasma

Daniela Sparvoli, Jason Delabre, Diana Penarete-Vargas, Shrawan Kumar Mageswaran, Lev Tsypin, Justine Heckendorn, Liam Theveny, Marjorie Maynadier, Marta Mendonça Cova, Laurence BERRY, Amandine Guérin, Jean-Francois Dubremetz, Serge Urbach, Boris Striepen, Aaron Turkewitz, Yi-Wei Chang and Maryse Lebrun
**DOI: 10.15252/embj.2022111158**

*Corresponding author: Maryse Lebrun (maryse.lebrun@umontpellier.fr)*



## Review Timeline:

| | |
|---|---|
| Submission Date: | 14th Mar 22 |
| Editorial Decision: | 4th Apr 22 |
| Revision Received: | 25th Jul 22 |
| Editorial Decision: | 18th Aug 22 |
| Revision Received: | 13th Sep 22 |
| Accepted: | 19th Sep 22 |

*Editor: David del Alamo/Ieva Gailite*


## Transaction Report:

(Note: With the exception of the correction of typographical or spelling errors that could be a source of ambiguity, letters and reports are not edited. Depending on transfer agreements, referee reports obtained elsewhere may or may not be included in this compilation. Referee reports are anonymous unless the Referee chooses to sign their reports.)

Thank you for submitting your manuscript for consideration by the EMBO Journal. We have now received comments from three reviewers, which are included below for your information.

As you will see from the comments, all reviewers appreciate the study, while also indicating a number of concerns that would have to be addressed and clarified before they can support publication of the manuscript. In particular, they point out various issues regarding data presentation and interpretation that would have to be addressed prior to acceptance. Based on these positive assessments, I would like to invite you to address the concerns raised by the reviewers in a revised manuscript.

We generally allow three months as standard revision time. As a matter of policy, competing manuscripts published during this period will not negatively impact on our assessment of the conceptual advance presented by your study. However, please contact me as soon as possible upon publication of any related work to discuss the appropriate course of action. Should you foresee a problem in meeting this three-month deadline, please let us know in advance and we may be able to grant an extension.

When preparing your letter of response to the referees' comments, please bear in mind that this will form part of the Review Process File and will therefore be available online to the community. For more details on our Transparent Editorial Process, please visit our website: https://www.embopress.org/transparent-process

Please feel free to contact me if have any further questions regarding the revision. Thank you for the opportunity to consider your work for publication. I look forward to receiving your revision.

------------------------------------------------

Referee #1:

Summary:

This study tackles a difficult and long-standing question of how apicomplexans recognise when to secrete their rhoptry organelle contents that comprise the molecules necessary for establishing the invasion structures in the host cell. The authors use some creative strategies drawing on their previous discoveries that elements of this process are conserved in ciliates, and I commend them for their creativity and willingness to span such wide organism systems. The fruits of these efforts are successful in that they identify molecules (CRMPs) that are required for the regulated secretory events but not otherwise necessary for the

assembly of either the rhoptry organelles or known structures at the plasma membrane that are known to mediate secretion. These discoveries represent key missing parts of this puzzle, and will usher in further mechanistic insights to this process, I'm sure. It is compelling that the molecules that have been discovered have domains that are consistent with interactions with host extracellular molecules. They propose a model where a pool of these proteins are presented to the extracellular milieu and mediate signalling events for rhoptry secretion upon contact with the cognate host cell molecules. The significance of this report both includes all apicomplexan invasion processes, but also the success of the evolutionary cell biological approach where important insights can be leveraged from relevant, but still somewhat distantly related, non(less)-canonical models.

Major concerns:

This study is thorough and generally executed very well. However, there are some elements of the strength or reach of the conclusions that go a little too far and I recommend that these are modified. Importantly, any exposure of the CRMPs to the exterior of the cell have not been demonstrated. While it is speculated that they are, and this is the linchpin of their model, it should be clearly acknowledged that this is currently unknown. Also, the argument that the CRMPs form a separate complex to Nd6 and other elements of the rhoptry secretion apparatus should be toned down. Absence of evidence of course is not evidence of absence, and while their tests for experimentally recoverable interactions yielded no clear result, it should be acknowledged that complex formation can by dynamic and/or labile (expanded on further in specific comments below). Similarly, the authors imply the speculation that the CRMPs are loaded into vesicles that ultimately might dock at the apex in a conoid-extrusion-dependent manner. This is an attractive model, but it should again be made clear that this is without concrete support at this stage. These concerns don't undermine the significance of the study's findings, but the reader should be given a more objective view of what is and is not clearly known.

Specific comments regarding text statements or figures that I should be addressed in the revision. Some concern the clarity of presentation, some the validity of the conclusions that are made.

Line 122: The phylogeny shown in Figure EV1A groups TgFer2 with this ciliate protein with branch support of only 55. The legend needs to explain what metric this is (I'd guess boostraps), but by any measure, this indicates that the data does not robustly support these clade associations, and this tree can't be used to make the stated conclusions. That the other three Tetrahymen proteins are not the Tg one's orthologues has not been excluded from this phylogenetic analysis.

Perhaps a comment for the editor. The first body of data is all in the 'Expanded View' section. This disrupts the continuity of the reading experience to not have relevant data directly presented with the narrative.

Figure 1A purports to show co-regulation of the genes of interest. I've no real reason not to believe in the outcome, but this figure alone doesn't show this without any data from other genes. Perhaps all genes show similar abundance changes? No metrics are given for coregulation. While I appreciate the motivation for showing this, it is not really a scientifically sound presentation or analysis of data. I think it is simply unnecessary here, but, if not, some indication of rest of the transcriptome is required.

Figure 1B: I don't find this workflow well explained. Abbreviations TGD/FGD need explaining in the legend. The text implies this is to find new exocytic factors in apicomplexans, yet the output of the diagram are Tet proteins. Similarly, the text from Lines 136-144 don't make the aims of this search very clear to me.

Line 208: I don't think the resolution of microscopy allows a conclusion of 'vesicle-like' staining, vesicles certainly can't be seen, and suggest a better descriptor. 'Dispersed in the cytosol', perhaps?

I'm dubious of the statistical analyses if they are performed on the normalised values, which the figures (e.g. Fig 2) suggest. If the control is the normalised reference for each replicate, and thus has no variance (100 +/- 0) then the variance of the other measures cannot be compared to it. The statistics all need to be performed against the raw measurements to be valid.

Line 292: replace 'and the protein completely disappeared' with 'and the protein was undetectable', because the former statement is almost impossible to verify.

Line 295: to test egress with a strong stimulant does not assess natural egress. It would be better to stated that stimulated egress was not affected. Also, the figure legend for FIgure EV3M is not present, it only goes to K.

Line 427: the hypothesis that the adhesion domain is on the extracellular side of the parasite membrane is attractive, but there has been no evidence presented at this point that delivery into the PM has occurred, nor even explicitly mentioned here. There is a leap of logic and support that hasn't been presented yet. The null hypothesis at this stage is that the apical dot is just within the lumen of the conoid space at this stage.

Figure EV4D: the plaque assay results are not very compelling with such a small field of view shown. A larger area of this tissue image would be advised.

Line 487: Is this remarkable given that it was already known that both occur as apical dots when conoids are excluded? This seems to be confirming something you already knew, or might have beenpredicted. Or was it expected that they would be apical but at different positions within the conoid space? Perhaps some more straightforward descriptors of the observations would be better.

Line 490: A disadvantage of such high resolution is that assumptions about how well the location of a terminal marker represent the whole protein can be challenged. What is the possible length (in nm) of these proteins, are how does this compare to the resolvable distances? This correlation is only considering where the individual reporter-tagged ends of these two proteins are, but the rest of the protein might not be spatially well represented. The extended conclusions of if Nd6 is or isn't part of the same complex based on the pulldown results and dependency of location of the other proteins should thus be tempered. They could all be part of the same complex still despite the data presented.

Line 502: An apical signal for TgCRMPa is not visible in the first panel representing the initial stage of invasion, so these data don't seem to report reliably on this dot signal. It would be useful to have an image of the cell from this experiment where the dot is still visible to demonstrate that it was detectable in these experimental conditions.

Line 569: I don't believe any experimental data showed protein exposure to the extracellular milieu, only that the other end of the protein was in the cytosol. So, interaction with the host cell membrane is speculation.

Line 571: the dependence of one protein on another for its location is not quite the same as evidence of formation of 'the productive complex'.

Line 572: was the co-location of CRIMPa and b assessed in the same was as that with Nd6? I don't think it was, and yet conclusions of complex association are made without such a control. It would be useful to explain why different methods were applied to different associations.

Line 638: yes, but perhaps you could point out that this is a system that looks specific to this group (Alveolata). There is no question of conserved secretion systems in eukaryotes, but your result is more interesting than that.

Additional comments:

A curious observation that is not discussed is how apparently abundant the CRMPs are. The fraction of protein that is presented in the apical dot during invasion, and potentially the critical protein, appears to be a tiny fraction of the total cell pool. Is this something that surprises the authors or not? Some comment would perhaps let the reader know if this observation could be considered significant or not. Could the CRMPs play any other roles that require some much protein?

Referee #2:

This is a nicely written manuscript that continues a theme linking the apical specialization of apicomplexan parasites with the secretory apparatus of ciliates. The studies compare Cysteine Rich Modular Proteins (CRMPs) that are orthologues between Toxoplasma/Plasmodium and Tetrahymena. The findings indicate an essential role for CRMPs in rhoptry discharge, but not in the formation of docking structures at the apical surface. The results are convincing and should be of general interest. I have a few comments and suggestions for improvements below.

Major points:

The distribution of CRMPS in the cytosol of tachyzoites is difficult to rationalize with their reported function. The functional pool of CRMPs is proposed to reside at the apical tip during conoid extension, and yet the vast majority of the proteins are found elsewhere in cytoplasmic granules. Do the authors have a plausible explanation for how CRMPs are recruited to the apical pole and why so much excess protein is retained in the cell, presumably in an non functional state. Or do CRMPs also function at other steps in membrane trafficking and fusion?

The authors suggest that CRMPs are transmembrane and extend the N terminus across the parasite plasma membrane to interact with the host cell. This raises some issues with topology. How are the CRMPs inserted into the membrane in this configuration? Does this occur by fusion of secretory vesicles that harbor CRMPs in their membrane, such that they are now externally exposed? More convincing data for the topology would be useful for the model. For example, the investigators could consider a N terminal tag that should be exposed on the external surface, and hence detected by IFA in the absence of detergent, or perform surface biotinylation on conoid extruded parasites.

Minor points:

Mutants in T gondii should be listed in lower case Δcrmp1 for example

Referee #3:

The manuscript "An apical membrane complex for triggering rhoptry exocytosis and invasion in Toxoplasma" by Sparvoli et al. reports on the functional characterisation of cysteine repeat modular proteins (CRMPs) in Tetrahymena and then in T. gondii, including the identification of two more CRMPs in T. gondii. These protein localise, at least in part, with the organisms exocytosis machinery and disruption of their function prevents exocytosis of Tetrahymena mucocysts and Toxoplasma rhoptries. The authors conclude that these proteins are involved in a new complex at the apical tip of the Toxoplasma tachyzoite that potentially interacts with the host cell and then signals for rhoptry secretion. The work is high-quality, the experiments well designed, the data is well presented and is mostly done with repeat and consistent measures. This is a very nice body of work and a pleasure to read. The study will be of broad interest due to the shared phylogeny of these exocytosis systems and their functional novelty.

Major comments:
Both of my major comments relate to the interpretation of the data. I would like to point out that I don't disagree with the interpretation, just that other possible roles for the proteins exists that are not being discussed to the same extent.
1) The HA tagged T. gondii CRMPs seem to localise throughout the parasite with the authors stating
'We observed diffuse vesicle-like staining of TgCRMPa-HA3 and TgCRMPb-HA3 throughout the entire parasite that disappears upon ATc incubation.'
The authors provide a model where CRMPs function is only at the apical tip. Yet, this amount of protein at this complex seems to be a small sum of the whole in many IFA images and is only there for a short time compared to other comparator proteins (Nd6). My question is, what could then be the function of the bulk of the CRMPs in the parasite cytoplasm? Do the authors have evidence for any other function or can they speculate on what the function of this excess protein is.
2) Cryo-EM measurements revealed that 'removal of CRMPb induces slight changes in the shape and anchoring angle of the AV.' And that this infers that 'there could be a direct or indirect connection of CRMPb to the AV which in turn could potentially regulate the rhoptry fusion apparatus.' This raises the possibility that it is the changes in shape that prevent rhoptry exocytosis and not signalling. As indicated by the authors, the loss of CRMPb could directly or indirectly impact on AV shape etc which in turn could impact on normal function of the exocytosis machinery. Is there any evidence that this is not the case or that signalling is definitely involved? Could this possibility be discussed more clearly?

Minor points:
-Line 826: 'Inducible auxin-inducible knockdown'. Perhaps just 'Auxin-inducible knockdown'?
Line 920: 'prior to be transferred'. To 'prior to being transferred'.

**Response to the reviewers**

We sincerely thank the reviewers for their thorough reading of the manuscript, for the constructive remarks and for raising key points of discussion important for improving the clarity of the presented findings.
Our answers to the reviewers' comments are indicated in **bold** and the changes added to the original text (black and *italic*) are reported in blue.

Referee#1

Major concerns:

This study is thorough and generally executed very well. However, there are some elements of the strength or reach of the conclusions that go a little too far and I recommend that these are modified. Importantly, any exposure of the CRMPs to the exterior of the cell have not been demonstrated. While it is speculated that they are, and this is the linchpin of their model, it should be clearly acknowledged that this is currently unknown. Also, the argument that the CRMPs form a separate complex to Nd6 and other elements of the rhoptry secretion apparatus should be toned down. Absence of evidence of course is not evidence of absence, and while their tests for experimentally recoverable interactions yielded no clear result, it should be acknowledged that complex formation can by dynamic and/or labile (expanded on further in specific comments below). Similarly, the authors imply the speculation that the CRMPs are loaded into vesicles that ultimately might dock at the apex in a conoid-extrusion-dependent manner. This is an attractive model, but it should again be made clear that this is without concrete support at this stage. These concerns don't undermine the significance of the study's findings, but the reader should be given a more objective view of what is and is not clearly known.

**We sincerely appreciate the reviewer's positive evaluation of our work. We agree that some conclusions were overstated. We generated new data to validate the topology of CRMPs and to demonstrate that the putative host binding domains are exposed extracellularly. In light of these new results, we have now revised the text to better clarify what has been formally demonstrated and what remains hypothetical.**

Specific comments regarding text statements or figures that I should be addressed in the revision. Some concern the clarity of presentation, some the validity of the conclusions that are made.

**1.** Line 122: The phylogeny shown in Figure EV1A groups TgFer2 with this ciliate protein with branch support of only 55. The legend needs to explain what metric this is (I'd guess boostraps), but by any measure, this indicates that the data does not robustly support these clade associations, and this tree can't be used to make the stated conclusions. That the other three Tetrahymen proteins are not the Tg one's orthologues has not been excluded from this phylogenetic analysis.

**We thank the reviewer for spotting this mistake, the numbers correspond indeed to the bootstrap values at each node and we added this information to the legend of the corresponding figure (now Fig 1A):**

**Lines 1526-1528:** *"The Tetrahymena homolog of the rhoptry-related TgFer2 (asterisk) is indicated by the red arrow.* Numbers at each node correspond to the bootstrap values.*"*

**We agree that the conclusion on the homology between *Tetrahymena* TTHERM_00886960 and TgFer2 is weakened by the bootstrap value 55, however a topology with low support values might still be closer to the real evolutionary relationship between the proteins involved. Moreover, the other three *Tetrahymena* ferlin proteins clearly clustered together in a separate well-supported clade. However, we understand the reviewer's concern on the phylogeny thus, we created a new phylogenetic tree by removing the ferlin sequences of the apicomplexan *Cryptosporidium parvum* (CPATCC) and the ciliate *Ichthyophthirius multifiliis* (IMG5), in order to obtain a more robust tree with well-supported clades. The new tree (now in Fig 1A) still reflects our conclusions and it substitutes the previous one in Fig. EV1A. We also updated the text accordingly:**

**Lines 122-124: "***Phylogenetic analysis of the four Tetrahymena ferlin genes revealed TTHERM_00886960 as the* putative *homolog of TgFer2* (Fig. 1A), while the other *Tetrahymena* ferlins appear to cluster together in a separate clade.*"*
**Lines 1521-1525:** *"The Maximum likelihood phylogenetic tree was obtained with the protein sequences of ferlin genes* retrieved for the ciliates *Tetrahymena thermophila* (TTHERM) and *Paramecium tetraurelia* (GSPATP), and for the apicomplexans *Toxoplasma gondii* (TGME49) and *Plasmodium falciparum* (PF3D7).*"*

**2.** Perhaps a comment for the editor. The first body of data is all in the 'Expanded View' section. This disrupts the continuity of the reading experience to not have relevant data directly presented with the narrative.

**We appreciate the reviewer's suggestion to the editor and follow his recommendation. We moved panels EV1A, D, and E to main figure 1 (now Fig. 1A, B, C), and left panels EV1B, C, F, G and H to figure EV1 (now Fig. EV1A, B, C, E, F).**

**3.** Figure 1A purports to show co-regulation of the genes of interest. I've no real reason not to believe in the outcome, but this figure alone doesn't show this without any data from other genes. Perhaps all genes show similar abundance changes? No metrics are given for coregulation. While I appreciate the motivation for showing this, it is not really a scientifically sound presentation or analysis of data. I think it is simply unnecessary here, but, if not, some indication of rest of the transcriptome is required.

**Since the rational of the screen is based on this co-regulation, we prefer to keep the graph in the main figure, and to make this data clearer, we added to the figure (now Fig 1D) another graph with the transcriptional profiles of *Tetrahymena* genes involved in different biological pathways for comparison. We also added the Y axis definition as "mRNA abundance" to clarify the metric used. We updated the text accordingly:**

**Lines 132-135:** *"Genes involved in mucocyst exocytosis are tightly co-regulated, as shown by the transcriptional profiles of Tetrahymena Nd6, Nd9, NdP1, NdP2 and Fer2 genes in different life stages* (Fig 1D, left), while genes involved in different pathways have non-matching profiles (Fig 1D, right)*."*

**Lines 1535-1539:**"*Expression profiles of Tetrahymena Nd genes involved in mucocysts exocytosis* (left graph), compared to those of genes functioning in different pathways (right graph: Cv, contractile vacuole, TTHERM_00532700; L, lysosomes, TTHERM_00716100; E, endosomes, TTHERM_00384890; E/Fv, endosomes/food vacuoles, TTHERM_00691590; Fv, food vacuoles, TTHERM_00393150) (Sparvoli et al., 2020)*."*

**4.** Figure 1B: I don't find this workflow well explained. Abbreviations TGD/FGD need explaining in the legend. The text implies this is to find new exocytic factors in apicomplexans, yet the output of the diagram are Tet proteins. Similarly, the text from Lines 136-144 don't make the aims of this search very clear to me.

**We rephrased the description of the transcriptomic approach to guarantee a better understanding of the strategy used, please see below:**

**Lines 137-149:** *"We took advantage of a bioinformatic tool specifically developed for Tetrahymena, called the Coregulation Data Harvester (CDH) (Tsypin & Turkewitz, 2017), to automate the search for* such *co-regulated genes* in the *Tetrahymena* databases (TGD, http://ciliate.org; FGD, http://tfgd.ihb.ac.cn). Since we were interested in genes with a conserved function in exocytosis in Alveolata, with a particular focus to rhoptry exocytosis in *Toxoplasma* and *Plasmodium*, we refined our analysis and *set up the CDH search to look for* *Tetrahymena* *genes conserved* specifically *in T. gondii and P. falciparum* (Fig 1E)*. We performed the CDH analysis using Tetrahymena Nd6, NdP1, NdP2 and Fer2 as separate queries, but excluded TtNd9 due to its very low expression level.* The CDH program *identified those Tetrahymena genes* co-regulated with each selected query, and *with homologs in T. gondii and P. falciparum,* by BLAST and reciprocal BLAST. We then *prioritized a list of candidates shared by at least three of the four queries (*Fig 1E *and* Dataset *EV1). Among the 37* *Tetrahymena* *candidates identified…"*

**We also added the description of the abbreviations TGD/FGD in the figure legend 1B (now Fig. 1E) as requested.**

**5.** Line 208: I don't think the resolution of microscopy allows a conclusion of 'vesicle-like' staining, vesicles certainly can't be seen, and suggest a better descriptor. 'Dispersed in the cytosol', perhaps?

**We agree and we changed the text accordingly:**

**Lines 190-191:** *"We observed a diffuse* punctate *staining of TgCRMPa-HA$_3$ and TgCRMPb-HA$_3$* dispersed in the *parasite* cytosol *that disappears upon ATc incubation."*

**6.** I'm dubious of the statistical analyses if they are performed on the normalised values, which

the figures (e.g. Fig 2) suggest. If the control is the normalised reference for each replicate, and thus has no variance (100 +/- 0) then the variance of the other measures cannot be compared to it. The statistics all need to be performed against the raw measurements to be valid.

**We repeated the statistical analyses, again on the normalized values, but taking in consideration the variance among the control replicates. We could not perform the analysis with the raw measurements because some of the assays are subjected to inevitable numeric variability among experiments performed in different days (biological replicates), although the ratio between the values measured for the control and the samples of interest during each experiment, is consistent across experiments. We updated the graphs for the egress, attachment, rhoptry secretion, invasion and plaque assays for TgCRMPs and Tg277910, by adding all the data points for all the technical replicates of the independent experiments. The values are still shown as mean ± SD and in the figure legends we wrote:** *"Values are reported as mean ± SD (n=x biological replicates, each with y technical replicates).* The biological replicates are represented by different symbols*."*

**7.** Line 292: replace 'and the protein completely disappeared' with 'and the protein was undetectable', because the former statement is almost impossible to verify.

**We changed the text, thank you:**

**Lines 226-228:** *"A single Tg277910-HA$_3$ band was detected by western blot in the absence of ATc, and the protein* was undetectable *after ATc treatment in both western blot (Fig EV3H) and IFA (Fig 3D and Fig EV3I)."*

**8.** Line 295: to test egress with a strong stimulant does not assess natural egress. It would be better to stated that stimulated egress was not affected. Also, the figure legend for FIgure EV3M is not present, it only goes to K.

**We now specify in the text that the step analyzed it was "stimulated egress", see below. The legend in figure EV3 has been corrected.**

**Lines 230-231:** *"…that was not related to the disruption of parasite replication,* stimulated *egress or attachment..."*
**Lines 1819-1820:** *"Fig EV2. TgCRMPa- and TgCRMPb-depleted tachyzoites have normal rhoptries, and show no defects in replication,* stimulated *egress and attachment. Related to Fig 2."*
**Line 1877:** *"*Stimulated *egress was quantified for…"*
**Lines 1892-1893:** *"Fig EV3. Tg277910-depleted tachyzoites with a disrupted lytic cycle, show no defects in microneme staining, replication,* stimulated *egress and attachment. Related to Fig 3."*
**Line 1950:** *"*K–M *Quantification of replication (K),* stimulated *egress (L) and attachment (M)…"*

**9.** Line 427: the hypothesis that the adhesion domain is on the extracellular side of the parasite

membrane is attractive, but there has been no evidence presented at this point that delivery into the PM has occurred, nor even explicitly mentioned here. There is a leap of logic and support that hasn't been presented yet. The null hypothesis at this stage is that the apical dot is just within the lumen of the conoid space at this stage.

**We agree that in the original manuscript we did not have formally demonstrated that the N-termini of CRMPs are exposed outside. Thus, as specifically requested by reviewer 2 (see below), we consolidated the data on the topology of CRMPs by performing new analyses on TgCRMPa. We experimentally validated that CRMPa is a transmembrane protein, exposing its putative binding domain outside and the C-terminal end toward the parasite cytosol. We added the new data on the revised version of the work as a separate section in the text (lines 286-331), and updated figures 5 and EV4 with panels E-K and G-O, respectively. A more detailed explanation of these experiments can be found below on the response to point 14.**

**10.** Figure EV4D: the plaque assay results are not very compelling with such a small field of view shown. A larger area of this tissue image would be advised.

**We replaced the image of the plaque assay in figure EV4D (now EV4J) with an updated one including the result of the N-terminally $HA_3$-miniAID-tagged CRMPa (see point 14).**

**11.** Line 487: Is this remarkable given that it was already known that both occur as apical dots when conoids are excluded? This seems to be confirming something you already knew, or might have beenpredicted. Or was it expected that they would be apical but at different positions within the conoid space? Perhaps some more straightforward descriptors of the observations would be better.

**This comment made us realizing that the aim of this experiment was not properly introduced. Here we wanted to know if CRMPs and Nd6 have a dynamic distribution during invasion.**
**We modified the text to better explain the reason for such experiment:**

**Lines 356-358:** "We next wondered whether CRMPs and Nd6 have a dynamic location during invasion and checked if *the apical dot labeled by CRMPs and Nd6 was maintained throughout the entire invasion process or limited to the pre-entry step.*"
**Lines 408-411:** *"Once the parasite breaches the host membrane the apical TgCRMPs staining disappears.* In contrast, TgNd6 signal persists, suggesting that the factors regulating the RSA machinery and the process of membrane fusion might still be present at the parasite apex upon rhoptry secretion. *Whether* CRMPs loss *is due to …"*

**12.** Line 490: A disadvantage of such high resolution is that assumptions about how well the location of a terminal marker represent the whole protein can be challenged. What is the possible length (in nm) of these proteins, are how does this compare to the resolvable distances? This correlation is only considering where the individual reporter-tagged ends of these two proteins are, but the rest of the protein might not be spatially well represented. The extended conclusions

of if Nd6 is or isn't part of the same complex based on the pulldown results and dependency of location of the other proteins should thus be tempered. They could all be part of the same complex still despite the data presented.

**We agree with the reviewer that the spatial organization of the entire protein might be underrepresented by the C-terminal epitope tagging when using this high resolution technique. In the revised manuscript we take into account the possibility of a labile complex, although we still favor our conclusions based on the reciprocal mass spectrometry analyses, Cryo-ET and immunofluorescence microscopy data, and also on the new data measuring the overlap between CRMPa and CRMPb co-expressed in the same cell line. Here the changes introduced in the text:**

**Lines 345-347:** *"Upon parasite expansion, we could measure a ~40% overlap between* C-terminally tagged *TgCRMPs*-$HA_3$ *and TgNd6*-$TY_2$ *at the tip of the extruded conoid (Fig 6C)…"*
**Lines 350-355:** *"…Nd9 mutant defective in RSA assembly (Fig 6D and Fig EV5E-G).* Nevertheless, this correlation is based on the detection of C-terminal markers which, in such high-resolution images, might not comprehensively reflect the spatial organization of the whole proteins. However, the colocalization analysis of co-expressed TgCRMPa-$TY_2$ and TgCRMPb-$HA_3$ (Fig. EV5H-J) provided, as expected for members of the same complex, a more robust overlap than the one between TgCRMPs and TgNd6 (Fig. 6B and C)*."*
**Lines 415-418:** *"CRMPs and their partners Tg247195 and Tg277910* seem to not be *part of the previously described Nd/NdP exocytic complex, also confirmed by a parallel study (Singer et al, 2022).* However, we cannot exclude the existence of a dynamic/transient complex formed by CRMPs and Nd proteins at the time of rhoptry exocytosis*."*

**We added the data on CRMPs overlap in figure 6 and EV5 and their corresponding legends, as well as in Material & Methods.**

**13.** Line 502: An apical signal for TgCRMPa is not visible in the first panel representing the initial stage of invasion, so these data don't seem to report reliably on this dot signal. It would be useful to have an image of the cell from this experiment where the dot is still visible to demonstrate that it was detectable in these experimental conditions.

**The image requested by the reviewer was in supplementary figure EV5H (now EV6) so, we moved it in main figure 6E.**

**14.** Line 569: I don't believe any experimental data showed protein exposure to the extracellular milieu, only that the other end of the protein was in the cytosol. So, interaction with the host cell membrane is speculation.

**In order to provide more convincing data on the topology of CRMPs and the exposure of the putative host cell binding domains to the extracellular media, we now added the results obtained by N-terminally tagging CRMPa. First, we introduced a triple HA followed by the auxin degron (miniAID) sequence after the signal peptide and before the MAR/Kringle domain (see updated figures 5 and EV4, and corresponding legends). In contrast with the**

**C-terminally miniAID-HA$_3$ tagged CRMPa, this fusion protein is not degraded upon auxin treatment, suggesting that the N-terminus of the protein likely faces the lumen of the putative transport vesicle. Of notice, the characterization of this N-terminally tagged version, revealed that CRMPa is proteolytically cleaved at the N-terminal end in an undefined site located downstream the tag, and that the immature form of CRMPa is not at the exocytic site in extracellular parasites. We then generated another cell line where we added a triple HA tag at the N-terminus but this time after the predicted Kringle domain. We detected the apical localization of this new fusion protein in extracellular parasites by immunofluorescence, in both permeabilizing and non-permeabilizing conditions, suggesting that the N-terminus of CRMPa is indeed oriented towards the extracellular space. We added these results to figure 5 and EV4, and updated the text by adding a new paragraph describing the data (Lines 286-331). We also highlighted these important new results at the end of the introduction section and in discussion:**
**Lines 106-110:** *"Sequence analyses of TgCRMPs showed that they are multipass-transmembrane proteins containing putative host cell binding domains.* Moreover, TgCRMPa is related to G protein-coupled receptor (GPCR) and exposes its host-cell binding domain towards the extracellular milieu upon egress.*"*
**Lines 388-390:** "Protein structure *predictions indicate that these domains* might be *exposed to the extracellular milieu,* thus likely capable of interacting with host cell membranes, and we experimentally validated this topology in the case of TgCRMPa.*"*
**Lines 422-424:** *"…and their topology at the membrane* with the putative host-binding domains exposed extracellularly, *all support a model where CRMPs and their associated factors interact with* surface *ligands presented by the host cell."*
**Lines 1684-1685:** *"Fig 5. TgCRMPa and TgCRMPb accumulate at the apical tip of extracellular tachyzoites,* with TgCRMPa N-terminal end oriented towards the outside space.*"*

**15.** Line 571: the dependence of one protein on another for its location is not quite the same as evidence of formation of 'the productive complex'.

**Agreed, we rephrased the corresponding sentences as follow:**

**Lines 280-282:** "…suggesting that the localization of TgCRMPb-HA$_3$ at the tip of extracellular parasites is dependent on the interaction with TgCRMPa."

**Lines 390-391:** *"This apical localization relies on the productive assembly of* CRMPs.*"*

**16.** Line 572: was the co-location of CRIMPa and b assessed in the same was as that with Nd6? I don't think it was, and yet conclusions of complex association are made without such a control. It would be useful to explain why different methods were applied to different associations.

**We added the colocalization analysis of CRMPs in the revised manuscript, as mentioned above. Please see our response at point 12.**

**17.** Line 638: yes, but perhaps you could point out that this is a system that looks specific to this

group (Alveolata). There is no question of conserved secretion systems in eukaryotes, but your result is more interesting than that.

**Thank you, we borrowed the suggested term "specific" and revised the text accordingly:**

**Lines 479-480:** *"…providing further support to the existence of a conserved machinery for secretion* specific to *Alveolata."*

Additional comments:

**18.** A curious observation that is not discussed is how apparently abundant the CRMPs are. The fraction of protein that is presented in the apical dot during invasion, and potentially the critical protein, appears to be a tiny fraction of the total cell pool. Is this something that surprises the authors or not? Some comment would perhaps let the reader know if this observation could be considered significant or not. Could the CRMPs play any other roles that require some much protein?

**We agree that this is an interesting point that we did not expand further in the text. We now added a comment in the discussion section about this localization:**

**Lines 390-405:** *"This apical localization relies on the productive assembly of* CRMPs. In addition, TgCMRPa apical signal is evident in both N- and C-terminally $HA_3$-tagged CRMPa lines, where two high-molecular-weight bands are detected by western blot, but not for the $HA_3$-miniAID-TgCRMPa (N-terminal tag) line, for which only the full-length protein seems to be present. These data suggest that it is mainly the processed form of TgCRMPa the one accumulating at the apical dot in extracellular parasites. We also observed an abundant dispersed localization of CRMPs within the parasite cytosol, similar to what previously seen for all the Nd proteins (Aquilini et al., 2021), although in the case of CRMPs the signal appears more apical, and partially overlapping with microneme proteins. CRMPs appears associated with vesicles since, by using the auxin degron system, the protein is degraded via proteasome only when the C-terminus, but not the N-terminus, is fused with the HA3-miniAID tag. This indicates that the N-terminal end of TgCRMPa is within the lumen of the transport vesicle and as such, protected from the effect of auxin. This suggests that CRMPs might be delivered to the apical end of the parasite upon vesicular trafficking and secretion, a scenario deserving further investigation, as well as whether the vast majority of the proteins found in the cytosolic fraction plays any role in addition to rhoptry exocytosis.*"*

Referee #2

This is a nicely written manuscript that continues a theme linking the apical specialization of apicomplexan parasites with the secretory apparatus of ciliates. The studies compare Cysteine Rich Modular Proteins (CRMPs) that are orthologues between Toxoplasma/Plasmodium and Tetrahymena. The findings indicate an essential role for CRMPs in rhoptry discharge, but not in the formation of docking structures at the apical surface. The results are convincing and should be of general interest. I have a few comments and suggestions for improvements below.

Major points:

**19.** The distribution of CRMPS in the cytosol of tachyzoites is difficult to rationalize with their reported function. The functional pool of CRMPs is proposed to reside at the apical tip during conoid extension, and yet the vast majority of the proteins are found elsewhere in cytoplasmic granules. Do the authors have a plausible explanation for how CRMPs are recruited to the apical pole and why so much excess protein is retained in the cell, presumably in an non functional state. Or do CRMPs also function at other steps in membrane trafficking and fusion?

**We agree with the reviewer that the abundance of the proteins within the cytosol it is an intriguing point. We now experimentally validated the topology of CRMPs (see the next point) and showed that inside the parasite, the C-terminus is facing the cytosol and the N-terminus is inside vesicles of unknown identity. Our data suggest that they are micronemes or microneme-like vesicles. This was discussed in the original manuscript (now lines 466 to 477). We added a comment in the discussion section to highlight the significance of this localization and to consider the possibility that this cytosolic fraction of CRMPs might be functional (lines 396-405). Please see our response to reviewer 1 at point 18.**

**19.** The authors suggest that CRMPs are transmembrane and extend the N terminus across the parasite plasma membrane to interact with the host cell. This raises some issues with topology. How are the CRMPs inserted into the membrane in this configuration? Does this occur by fusion of secretory vesicles that harbor CRMPs in their membrane, such that they are now externally exposed? More convincing data for the topology would be useful for the model. For example, the investigators could consider a N terminal tag that should be exposed on the external surface, and hence detected by IFA in the absence of detergent, or perform surface biotinylation on conoid extruded parasites.

**We thank the reviewer and following her/his suggestion, we generated two new constructs. In the first one, we added a $HA_3$-miniAID at the N-terminus of CRMPa (before the MAR/Kringle domain) and showed that the protein is not targeted to the proteasome for degradation (in contrast to the fusion at the C-terminus). This supports the fact that CRMPa is an integral membrane protein associated with intracellular vesicles. Secondly, we added a triple HA again at the N-terminus (after the MAR/Kringle), and showed that this tag is exposed extracellularly, since the apical signal persists in absence of parasite permeabilization. These results support a model in which CRMPs are translocated to the**

**apical end of the parasite upon fusion of vesicles with the parasite plasma membrane. These data are presented in figures 5 and EV4. We invite the reviewer to check also our reply to reviewer 1 about the CRMPs topology (point 14).**

Minor points:
Mutants in T gondii should be listed in lower case Δcrmp1 for example

**Thank you, the mutants' names have been changed throughout the text and figures as suggested.**

Referee #3

The manuscript "An apical membrane complex for triggering rhoptry exocytosis and invasion in Toxoplasma" by Sparvoli et al. reports on the functional characterisation of cysteine repeat modular proteins (CRMPs) in Tetrahymena and then in T. gondii, including the identification of two more CRMPs in T. gondii. These protein localise, at least in part, with the organisms exocytosis machinery and disruption of their function prevents exocytosis of Tetrahymena mucocysts and Toxoplasma rhoptries. The authors conclude that these proteins are involved in a new complex at the apical tip of the Toxoplasma tachyzoite that potentially interacts with the host cell and then signals for rhoptry secretion. The work is high-quality, the experiments well designed, the data is well presented and is mostly done with repeat and consistent measures. This is a very nice body of work and a pleasure to read. The study will be of broad interest due to the shared phylogeny of these exocytosis systems and their functional novelty.

Major comments:

**21.** Both of my major comments relate to the interpretation of the data. I would like to point out that I don't disagree with the interpretation, just that other possible roles for the proteins exists that are not being discussed to the same extent.

1) The HA tagged T. gondii CRMPs seem to localise throughout the parasite with the authors stating

'We observed diffuse vesicle-like staining of TgCRMPa-HA3 and TgCRMPb-HA3 throughout the entire parasite that disappears upon ATc incubation.'

The authors provide a model where CRMPs function is only at the apical tip. Yet, this amount of protein at this complex seems to be a small sum of the whole in many IFA images and is only there for a short time compared to other comparator proteins (Nd6). My question is, what could then be the function of the bulk of the CRMPs in the parasite cytoplasm? Do the authors have evidence for any other function or can they speculate on what the function of this excess protein is.

**We agree and we invite this reviewer to check our reply to reviewer 1 and 2, which can be found above at points 18 and 19.**

2) Cryo-EM measurements revealed that 'removal of CRMPb induces slight changes in the shape and anchoring angle of the AV.' And that this infers that 'there could be a direct or indirect connection of CRMPb to the AV which in turn could potentially regulate the rhoptry fusion apparatus.' This raises the possibility that it is the changes in shape that prevent rhoptry exocytosis and not signalling. As indicated by the authors, the loss of CRMPb could directly or indirectly impact on AV shape etc which in turn could impact on normal function of the exocytosis machinery. Is there any evidence that this is not the case or that signalling is definitely involved? Could this possibility be discussed more clearly?

**Thank you for this comment, in the manuscript we favor the signaling-based model because of the structural features of CRMPs and their dynamic localization. The changes in the shape of the AV are relatively minor compared to the wildtype condition, and do not seem to affect the correct assembly of the RSA at the PPM, which is an essential pre-requisite for efficient rhoptry secretion. At the moment we do not have evidences to affirm that the AV change is a consequence of the signaling function of CRMPb, or if the loss of**

**CRMPb locally affects the AV by, for example, impeding the recruitment of other factors essential for maintaining the vesicle well-shaped, and for rhoptry fusion. We are opened to this second possibility and stated this concept in the text:**

**Lines 425-434:** *"Intriguingly, removal of CRMPb induces slight changes in the shape and anchoring angle of the AV,* while the RSA at the PPM is correctly assembled, which is an essential pre-requisite for efficient rhoptry secretion. *Albeit the changes* of the AV *are relatively minor, they infer that there could be a direct or indirect connection of CRMPb to the AV which in turn could potentially regulate the rhoptry fusion apparatus.* What remains unknown is whether these changes are a consequence of the signaling function of CRMPb, or if the loss of CRMPb locally affects the AV by, for example, impeding the recruitment of other factors essential for maintaining the vesicle well-shaped and fit for fusion.
*Our* "signaling-based" *model is also supported by…"*

Minor points:
-Line 826: 'Inducible auxin-inducible knockdown'. Perhaps just 'Auxin-inducible knockdown'?
Line 920: 'prior to be transferred'. To 'prior to being transferred'.

**Thank you for spotting these mistakes, they have been corrected.**

## 1st Revision - Editorial Decision 18th Aug 2022

Thank you for submitting a revised version of your manuscript. Your study has now been seen by two of the original referees, who find that most of their major concerns have been addressed. There remain only a couple of mainly editorial issues that have to be solved before I can extend formal acceptance of the manuscript:

1. Please address the final two points from reviewer #1 by adjusting the statements on TTHERM_00886960 evolutionary conservation and co-regulation of mucocyst exocytosis genes.
2. Our publisher has done their pre-publication check on your manuscript. I have attached the file below. Please take a look at the Word file and the comments in the figure legends and respond to the issues. Please also use this version when you resubmit the revised version.
3. Please make sure that the funding information in the manuscript and in our online submission system is complete. Currently information on FACCTS, European Union's Horizon 2020 research and innovation program under Grant agreement no. 833309 is missing in our online submission system.
4. We generally encourage publication of source data for electrophoretic gels and blots, with the aim of making primary data more accessible and transparent to the reader. We would need one file per figure (which can be a composite of source data from several panels) in jpg, gif or PDF format, uploaded as "Source data files". The gels should be labeled with the appropriate figure/panel number, and should have molecular weight markers; further annotation would clearly be useful but is not essential. These files will be published online with the article as supplementary "Source Data". Please let me know if you have any questions about this policy.
5. Papers published in The EMBO Journal are accompanied online by a 'Synopsis' to enhance discoverability of the manuscript. Please submit a brief textual summary of your study consisting of a short (1-2 sentences) summary of the findings and their significance and 3-4 bullet points highlighting the key results.

Please let me know if you have any further questions regarding any of these points. You can use the link below to upload the revised files.

Thank you again for giving us the chance to consider your manuscript for The EMBO Journal. I look forward to receiving the final version.

------------------------------------------------

Referee #1:

Most of my queries have been very well dealt with, and the authors have done an excellent job with further work, particularly showing the surface exposure at the apex of CRMPa. I still have concerns with two, however.

1) Phylogenetic analysis: I'm sorry but I am not satisfied with the revision with respect to identifying the likely orthologue of

TgFer2. There is already a language problem. All genes derived from a common ancestral molecular are homologues. It is an assumption of any phyologeny that all molecular are homologous. What the authors are aim to identity is if there is an orthologue of TgFer2 in ciliates over possible paralogues. All phylogeny topologies are simply hypotheses for possible relationships, and the output of most phylogenetic analyses is the best scoring topology given the data. However, there might be many alternative topologies that receive very nearly as good support from the data. Bootstrapping is one way to test how well a given relationship in the best topology is supported by the data. The initial evaluation was that TgFer2 and TTHERM_00886960 are ortholgues to the exclusion of other ciliate sequences was revealed to have negligible support - this relationship in the 'best tree' was little more than chance. The authors argue that 55 is better than nothing. But if you flip a coin once you are unlikely to conclude that it will always land heads-up from there on for all subsequent flips. The authors' response to this indication of poor support is to remove sequences until they achieve the support that they apparently sought. I find this troubling. Phylogenies will collapse to a stronger simpler topology with data removed. But no justification was given as to why these sequences were identified as likely causing false signal in the phylogeny, which would be the only justification for their removal. On the contrary, any hypothesis should be able to be better tested by adding more sequences as the source of phylogenetic signal. The reality is that some molecules, after sufficient evolutionary time, will not have sufficient signal of their past to enable supported phylogenetic reconstruction. This is almost certainly the case here. This doesn't mean that the authors' hypothesis that TTHERM_00886960 is not the orthologue of TgFer2, only that this is a question that cannot be tested with these data.

2). Coregulation of genes: I appreciate the extra figure, but there is still no robust analysis or metric presented that supports coregulation. Select single gene examples are given for different biological pathways, but it is still not evident if these are representative either. For the conclusion that "Genes involved in mucocyst exocytosis are tightly co-regulated" to be made there simply must be a quantitative analysis. If, on the other hand, the authors wanted to say something like "that we anecdotally observed similar patterns of expression of known genes involved in mycocyst exocytosis, therefore we employed . . . . to screen for other proteins sharing similar patterns" this would be fine. But conclusions of tight coregulation require a sound method to test for this over alternative explanations of the patterns chosen for display in the figures.

Referee #2:

All of my concerns have been addressed by the inclusion of new data and revision of the text.

**Response to the reviewers**

Once more, we sincerely thank the reviewers for their appreciation of our work and additional comments.
Our answers to the reviewer 1's points are indicated in **bold** and the changes added to the original/already-revised text (black/blue and *italic*) are reported in green.

Referee #1:

Most of my queries have been very well dealt with, and the authors have done an excellent job with further work, particularly showing the surface exposure at the apex of CRMPa. I still have concerns with two, however.

1) Phylogenetic analysis: I'm sorry but I am not satisfied with the revision with respect to identifying the likely orthologue of TgFer2. There is already a language problem. All genes derived from a common ancestral molecular are homologues. It is an assumption of any phyologeny that all molecular are homologous. What the authors are aim to identity is if there is an orthologue of TgFer2 in ciliates over possible paralogues. All phylogeny topologies are simply hypotheses for possible relationships, and the output of most phylogenetic analyses is the best scoring topology given the data. However, there might be many alternative topologies that receive very nearly as good support from the data. Bootstrapping is one way to test how well a given relationship in the best topology is supported by the data. The initial evaluation was that TgFer2 and TTHERM_00886960 are ortholgues to the exclusion of other ciliate sequences was revealed to have negligible support – this relationship in the 'best tree' was little more than chance. The authors argue that 55 is better than nothing. But if you flip a coin once you are unlikely to conclude that it will always land heads-up from there on for all subsequent flips. The authors' response to this indication of poor support is to remove sequences until they achieve the support that they apparently sought. I find this troubling. Phylogenies will collapse to a stronger simpler topology with data removed. But no justification was given as to why these sequences were identified as likely causing false signal in the phylogeny, which would be the only justification for their removal. On the contrary, any hypothesis should be able to be better tested by adding more sequences as the source of phylogenetic signal. The reality is that some molecules, after sufficient evolutionary time, will not have sufficient signal of their past to enable supported phylogenetic reconstruction. This is almost certainly the case here. This doesn't mean that the authors' hypothesis that THERM_00886960 is not the orthologue of TgFer2, only that this is a question that cannot be tested with these data.

**We thank the reviewer for the insightful comment on the ferlin phylogeny and especially for "pushing" us to repeat the analysis. In fact, we added more sequences from other apicomplexans to the initial dataset, and we obtained a better branch support (75) for the relationship between the *Tetrahymena* protein TTHERM_00886960 and TgFer2. However, this phylogeny alone does not formally conclude that they are true orthologs since, as mentioned by the reviewer, they likely lost sufficient signal to enable supported phylogenetic reconstruction due to their long evolutionary time. In the revision, we took more caution in referring to the *Tetrahymena* TTHERM_00886960 as TgFer2 ortholog.**

**We replaced the previous phylogeny in figure 1A with the new one and updated the corresponding legend, we added the new apicomplexan ferlin sequences in Dataset EV4, and updated the text emphasizing that the phylogeny output is indeed a prediction and not an absolute answer:**
**Lines 121-129:** *"To test a conserved role of Fer2 in the two systems, we searched for the Tetrahymena* ortholog *of TgFer2 and verified its role in exocytosis.* Our *phylogenetic analysis of the four Tetrahymena ferlin genes* predicted *TTHERM_00886960 as the putative* ortholog *of TgFer2 (Fig 1A), while the other Tetrahymena ferlins* belong to a separate subgroup. However, this phylogeny alone does not formally conclude that they are true orthologs. To support such evolutionary relationship, we investigated this prediction experimentally by deleting *the expressed (macronuclear) copies of the TtFer2* candidate in *Tetrahymena* cells *(Fig EV1A and B). We found that the Δ00886960* (*Δfer2*) *mutant cells have a defect in mucocyst release when stimulated with the secretagogue dibucaine (Fig 1B),* although *the organelles appeared properly formed and docked at the plasma membrane (Fig 1C)."*
**Lines 131-134:** *"These results demonstrate a role for* TTHERM_00886960 *in exocytosis,* and support TTHERM_00886960 as the ortholog of apicomplexans Fer2, further highlighting *the conservation of exocytic mechanisms in Alveolata."*

2). Coregulation of genes: I appreciate the extra figure, but there is still no robust analysis or metric presented that supports coregulation. Select single gene examples are given for different biological pathways, but it is still not evident if these are representative either. For the conclusion that "Genes involved in mucocyst exocytosis are tightly co-regulated" to be made there simply must be a quantitative analysis. If, on the other hand, the authors wanted to say something like "that we anecdotally observed similar patterns of expression of known genes involved in mycocyst exocytosis, therefore we employed . . . . to screen for other proteins sharing similar patterns" this would be fine. But conclusions of tight coregulation require a sound method to test for this over alternative explanations of the patterns chosen for display in the figures.

**We understand the reviewer's concern in fact, what we meant in the manuscript for "co-regulation" is indeed "co-expression", and we changed the text following the reviewer's advice:**
**Lines 93-96:** "*To further exploit this phenomenon, we used the Coregulation Data Harvester (CDH) tool (Tsypin & Turkewitz, 2017) to automate the search of genes* with expression patterns similar to those of the *Tetrahymena Nd genes and also conserved in Apicomplexa*."
**Lines 135-141:** *"Genes involved in mucocyst exocytosis* share similar patterns of expression*, as shown by the transcriptional profiles of Tetrahymena Nd6, Nd9, NdP1, NdP2 and Fer2 genes in different life stages (Fig 1D, left), while genes involved in different pathways have non-matching profiles (Fig 1D, right).* We therefore employed a bioinformatic tool specifically developed for *Tetrahymena*, called the Coregulation Data Harvester (CDH) (Tsypin & Turkewitz, 2017), to screen for other proteins with comparable patterns *in the Tetrahymena databases (TGD, http://ciliate.org; FGD, http://tfgd.ihb.ac.cn)."*

**In addition, all the "co-regulated" terms have been replaced with "co-expressed" including those in the Dataset EV1.**

## 2nd Revision - Editorial Decision 19th Sep 2022

Thank you for addressing the final minor issues in a revised version of your manuscript. I am now pleased to inform you that your manuscript has been accepted for publication in The EMBO Journal.

## EMBO Press Author Checklist

| |
|---|
| Corresponding Author Name: Maryse Lebrun |
| Journal Submitted to: The EMBO Journal |
| Manuscript Number: EMBOJ-2022-111158 |

**USEFUL LINKS FOR COMPLETING THIS FORM**
The EMBO Journal - Author Guidelines
EMBO Reports - Author Guidelines
Molecular Systems Biology - Author Guidelines
EMBO Molecular Medicine - Author Guidelines

**Reporting Checklist for Life Science Articles (updated January 2022)**
This checklist is adapted from Materials Design Analysis Reporting (MDAR) Checklist for Authors. MDAR establishes a minimum set of requirements in transparent reporting in the life sciences (see Statement of Task: 10.31222/osf.io/9sm4x). Please follow the journal's guidelines in preparing your manuscript.
**Please note that a copy of this checklist will be published alongside your article.**

**Abridged guidelines for figures**
**1. Data**
The data shown in figures should satisfy the following conditions:
- the data were obtained and processed according to the field's best practice and are presented to reflect the results of the experiments in an accurate and unbiased manner.
- ideally, figure panels should include only measurements that are directly comparable to each other and obtained with the same assay.
- plots include clearly labeled error bars for independent experiments and sample sizes. Unless justified, error bars should not be shown for technical replicates.
- if n<5, the individual data points from each experiment should be plotted. Any statistical test employed should be justified.
- Source Data should be included to report the data underlying figures according to the guidelines set out in the authorship guidelines on Data Presentation.

**2. Captions**
Each figure caption should contain the following information, for each panel where they are relevant:
- a specification of the experimental system investigated (eg cell line, species name).
- the assay(s) and method(s) used to carry out the reported observations and measurements.
- an explicit mention of the biological and chemical entity(ies) that are being measured.
- an explicit mention of the biological and chemical entity(ies) that are altered/varied/perturbed in a controlled manner.
- the exact sample size (n) for each experimental group/condition, given as a number, not a range;
- a description of the sample collection allowing the reader to understand whether the samples represent technical or biological replicates (including how many animals, litters, cultures, etc.).
- a statement of how many times the experiment shown was independently replicated in the laboratory.
- definitions of statistical methods and measures:
  - common tests, such as t-test (please specify whether paired vs. unpaired), simple χ2 tests, Wilcoxon and Mann-Whitney tests, can be unambiguously identified by name only, but more complex techniques should be described in the methods section;
  - are tests one-sided or two-sided?
  - are there adjustments for multiple comparisons?
  - exact statistical test results, e.g., P values = x but not P values < x;
  - definition of 'center values' as median or average;
  - definition of error bars as s.d. or s.e.m.

**Please complete ALL of the questions below.**
**Select "Not Applicable" only when the requested information is not relevant for your study.**

**Materials**

| Newly Created Materials | Information included in the manuscript? | In which section is the information available? (Reagents and Tools Table, Materials and Methods, Figures, Data Availability Section) |
|---|---|---|
| New materials and reagents need to be available; do any restrictions apply? | Yes | Materials and Methods |

| Antibodies | Information included in the manuscript? | In which section is the information available? (Reagents and Tools Table, Materials and Methods, Figures, Data Availability Section) |
|---|---|---|
| For **antibodies** provide the following information:<br>- Commercial antibodies: RRID (if possible) or supplier name, catalogue number and or/clone number<br>- Non-commercial: RRID or citation | Yes | Materials and Methods |

| DNA and RNA sequences | Information included in the manuscript? | In which section is the information available? (Reagents and Tools Table, Materials and Methods, Figures, Data Availability Section) |
|---|---|---|
| **Short novel DNA or RNA including primers, probes**: provide the sequences. | Yes | Materials and Methods/Table EV5 |

| Cell materials | Information included in the manuscript? | In which section is the information available? (Reagents and Tools Table, Materials and Methods, Figures, Data Availability Section) |
|---|---|---|
| **Cell lines:** Provide species information, strain. Provide accession number in repository **OR** supplier name, catalog number, clone number, and/**OR** RRID. | Yes | Materials and Methods |
| **Primary cultures:** Provide species, strain, sex of origin, genetic modification status. | Not Applicable | |
| Report if the cell lines were recently **authenticated** (e.g., by STR profiling) and tested for mycoplasma contamination. | Not Applicable | |

| Experimental animals | Information included in the manuscript? | In which section is the information available? (Reagents and Tools Table, Materials and Methods, Figures, Data Availability Section) |
|---|---|---|
| **Laboratory animals or Model organisms:** Provide species, strain, sex, age, genetic modification status. Provide accession number in repository **OR** supplier name, catalog number, clone number, **OR** RRID. | Not Applicable | |
| **Animal observed in or captured from the field:** Provide species, sex, and age where possible. | Not Applicable | |
| Please detail **housing and husbandry conditions**. | Not Applicable | |

| Plants and microbes | Information included in the manuscript? | In which section is the information available? (Reagents and Tools Table, Materials and Methods, Figures, Data Availability Section) |
|---|---|---|
| **Plants:** provide species and strain, ecotype and cultivar where relevant, unique accession number if available, and source (including location for collected wild specimens). | Not Applicable | |
| **Microbes:** provide species and strain, unique accession number if available, and source. | Yes | Materials and Methods |

| Human research participants | Information included in the manuscript? | In which section is the information available? (Reagents and Tools Table, Materials and Methods, Figures, Data Availability Section) |
|---|---|---|
| If collected and within the bounds of privacy constraints report on age, sex and gender or ethnicity for all study participants. | Not Applicable | |

| Core facilities | Information included in the manuscript? | In which section is the information available? (Reagents and Tools Table, Materials and Methods, Figures, Data Availability Section) |
|---|---|---|
| If your work benefited from core facilities, was their service mentioned in the acknowledgments section? | Yes | Materials and Methods |

**Design**

| Study protocol | Information included in the manuscript? | In which section is the information available? (Reagents and Tools Table, Materials and Methods, Figures, Data Availability Section) |
|---|---|---|
| If study protocol has been **pre-registered, provide DOI in the manuscript**. For clinical trials, provide the trial registration number **OR** cite DOI. | Not Applicable | |
| Report the **clinical trial registration number** (at ClinicalTrials.gov or equivalent), where applicable. | Not Applicable | |

| Laboratory protocol | Information included in the manuscript? | In which section is the information available? (Reagents and Tools Table, Materials and Methods, Figures, Data Availability Section) |
|---|---|---|
| Provide DOI OR other citation details if **external detailed step-by-step protocols** are available. | Yes | Materials and Methods |

| Experimental study design and statistics | Information included in the manuscript? | In which section is the information available? (Reagents and Tools Table, Materials and Methods, Figures, Data Availability Section) |
|---|---|---|
| Include a statement about **sample size** estimate even if no statistical methods were used. | Yes | Materials and Methods/Figures/Tables |
| Were any steps taken to minimize the effects of subjective bias when allocating animals/samples to treatment (e.g. **randomization procedure**)? If yes, have they been described? | Not Applicable | |
| Include a statement about **blinding** even if no blinding was done. | Not Applicable | |
| Describe **inclusion/exclusion criteria** if samples or animals were excluded from the analysis. Were the criteria pre-established?<br><br>If sample or data points were omitted from analysis, report if this was due to attrition or intentional exclusion and provide justification. | Not Applicable | |
| For every figure, are **statistical tests** justified as appropriate? Do the data meet the assumptions of the tests (e.g., normal distribution)? Describe any methods used to assess it. Is there an estimate of variation within each group of data? Is the variance similar between the groups that are being statistically compared? | Yes | Materials and Methods/Figures |

| Sample definition and in-laboratory replication | Information included in the manuscript? | In which section is the information available? (Reagents and Tools Table, Materials and Methods, Figures, Data Availability Section) |
|---|---|---|
| In the figure legends: state number of times the experiment was **replicated** in laboratory. | Yes | Materials and Methods/Figures |
| In the figure legends: define whether data describe **technical or biological replicates**. | Yes | Materials and Methods/Figures |

## Ethics

| Ethics | Information included in the manuscript? | In which section is the information available? (Reagents and Tools Table, Materials and Methods, Figures, Data Availability Section) |
|---|---|---|
| Studies involving **human participants**: State details of **authority granting ethics approval** (IRB or equivalent committee(s), provide reference number for approval. | Not Applicable | |
| Studies involving **human participants**: Include a statement confirming that **informed consent** was obtained from all subjects and that the experiments conformed to the principles set out in the WMA Declaration of Helsinki and the Department of Health and Human Services Belmont Report. | Not Applicable | |
| Studies involving **human participants:** For publication of **patient photos**, include a statement confirming that consent to publish was obtained. | Not Applicable | |
| Studies involving experimental **animals**: State details of **authority granting ethics approva**l (IRB or equivalent committee(s), provide reference number for approval. Include a statement of compliance with ethical regulations. | Not Applicable | |
| Studies involving **specimen and field samples:** State if relevant **permits** obtained, provide details of authority approving study; if none were required, explain why. | Not Applicable | |

| Dual Use Research of Concern (DURC) | Information included in the manuscript? | In which section is the information available? (Reagents and Tools Table, Materials and Methods, Figures, Data Availability Section) |
|---|---|---|
| Could your study fall under dual use research restrictions? Please check biosecurity documents and list of **select agents and toxins** (CDC): https://www.selectagents.gov/sat/list.htm | Not Applicable | |
| If you used a select agent, is the security level of the lab appropriate and reported in the manuscript? | Not Applicable | |
| If a study is subject to dual use research of concern regulations, is the name of the **authority granting approval and reference number** for the regulatory approval provided in the manuscript? | Not Applicable | |

## Reporting

The MDAR framework recommends adoption of discipline-specific guidelines, established and endorsed through community initiatives. Journals have their own policy about requiring specific guidelines and recommendations to complement MDAR.

| Adherence to community standards | Information included in the manuscript? | In which section is the information available? (Reagents and Tools Table, Materials and Methods, Figures, Data Availability Section) |
|---|---|---|
| State if relevant guidelines or checklists (e.g., **ICMJE, MIBBI, ARRIVE, PRISMA**) have been followed or provided. | Not Applicable | |
| For **tumor marker prognostic studies,** we recommend that you follow the **REMARK** reporting guidelines (see link list at top right). See author guidelines, under 'Reporting Guidelines'. Please confirm you have followed these guidelines. | Not Applicable | |
| For **phase II and III randomized controlled trials**, please refer to the **CONSORT** flow diagram (see link list at top right) and submit the CONSORT checklist (see link list at top right) with your submission. See author guidelines, under 'Reporting Guidelines'. Please confirm you have submitted this list. | Not Applicable | |

## Data Availability

| Data availability | Information included in the manuscript? | In which section is the information available? (Reagents and Tools Table, Materials and Methods, Figures, Data Availability Section) |
|---|---|---|
| Have **primary datasets** been deposited according to the journal's guidelines (see 'Data Deposition' section) and the respective accession numbers provided in the Data Availability Section? | Yes | Data Availability Section |
| Were **human clinical and genomic datasets** deposited in a public access-controlled repository in accordance to ethical obligations to the patients and to the applicable consent agreement? | Not Applicable | |
| Are **computational models** that are central and integral to a study available without restrictions in a machine-readable form? Were the relevant accession numbers or links provided? | Not Applicable | |
| If publicly available data were reused, provide the respective **data citations in the reference list**. | Not Applicable | |
